# Bread or roses? Trade unions, female employment and the expansion of work-family policies

**DOI:** 10.1080/13501763.2023.2184414

**Published:** 2023-03-06

**Authors:** Luca Michele Cigna

**Affiliations:** European University Institute, Fiesole, Italy

**Keywords:** Gender, social policy, trade unions, welfare state

## Abstract

In the Fordist era, trade unions promoted welfare state expansion and coverage against risks for the broader workforce. With the shift to the post-industrial economy, however, new economic groups have been left without representation. This is particularly evident for women: despite a rapid increase in female employment since the 1980s, unions’ membership base remains anchored in the male, old and industrial working class. Without the crucial pressure of labour, welfare systems have failed to enhance the reconciliation of work and family life. Under which conditions do unions support the expansion of work-family policies? Marshalling evidence from 20 OECD countries in the 1980–2010 period, this paper investigates the role of political actors in family policy reform. Findings suggest that unions promote the expansion of work-family packages when they are gender-inclusive and have institutional access to policy-making.

## Introduction

New labour market groups such as women, young people and atypical workers are often seen as the losers of the post-industrial era (Emmenegger *et al*., 2012; Palier & Thelen, 2010; Rueda *et al*., 2015). The so-called ‘outsiders’ fail to enjoy the same levels of social services and benefits as the industrial working class (Schwander & Häusermann, 2013). One of the main reasons why they fall outside of the welfare state ‘paradise’ is that they confront tremendous collective action problems (Bonoli, 2005; Gumbrell-McCormick & Hyman, 2013). While in the Fordist era, the labour movement spoke on behalf of the disenfranchized to advance their social rights, since the 1980s the new workforce has been largely left without representation (Bonoli & Natali, 2012; Rehm, 2011). This is particularly evident for women: despite a rapid increase in female employment since the 1980s, welfare systems are path-dependent to a male breadwinner understanding of gender relations, which crystallizes existing disparities in work-family burdens within the household (Daly & Ferragina, 2018).

**Figure 1.**
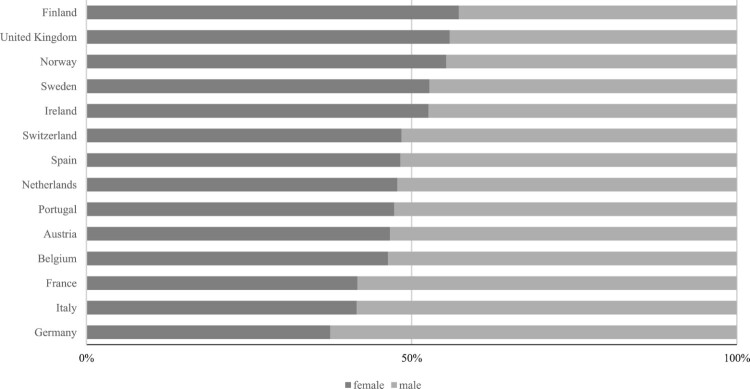
Relative union membership rates by gender in 14 countries. Own elaboration. Data: ESS (2018).

In the recent comparative political economy (CPE) literature, trade unions are often presented as particularistic interests that lobby against welfare state recalibration (Palier & Thelen, 2010; Rueda, 2007). As labour markets become more diverse, unions face conflicting interests between new groups and the old industrial and male workforce, resulting in membership gender gaps (Figure 1). According to dualization scholars, the disproportion of (male) ‘insiders’ vis à vis more peripheral segments is the reason why unions and social democratic parties neglect the functional readaptation of modern welfare states to families and children, and act as mere defenders of the status quo (Beramendi *et al*., 2015; Rueda, 2007). In recent decades, unions have generally pushed for maintaining traditional social protection (e.g., pensions and unemployment support) for core workers, while consenting to the reduction of benefits for atypical workers (Häusermann, 2010; Palier & Thelen, 2010). However, it is well documented that unions can also act as a ‘sword of justice’, pursuing long-term goals and normative ambitions (Gumbrell-McCormick & Hyman, 2013; Scharpf, 1997). Secondly, empirical evidence runs against the idea of unions as ‘institutional dinosaurs’ and hence opponents to reforms. In some instances, they have opened their ranks to collaborate with governments on the expansion of new social policies (Clegg & van Wijnbergen, 2011; Häusermann, 2018; Johnston *et al*., 2012; Ornston, 2013).

Countering the image of unions as clubs of ‘old white males’, labour market changes have a significant impact on unions and their composition (Visser, 2019). While unions lag behind in sectors where women are overrepresented, female membership is on the rise all around the world; in no less than eight advanced economies, more women than men are currently union members (Schnabel, 2013). Female-dominated industries such as the public and socio-cultural sector now display higher unionization rates than average (Hassel, 2015; Oesch, 2006). Changes in labour structures should motivate unions to promote female employment and work-family reconciliation (Bledow, 2021; Doellgast *et al*., 2018). Yet, in the face of rising female membership and the urge of institutional adaptation, union generally fail to champion these demands, settling for the narrow defence of core agenda items. Current research has not yet definitively assessed what institutional or political factors spur unions to embrace new social needs in the context of welfare reform. Does women’s involvement within trade unions affect the expansion of work-family policies (WFP)? Under which institutional and organizational conditions do unions support welfare recalibration in this field?

Marshalling evidence from 20 OECD countries in the 1980–2010 period, this paper investigates unions’ contributions to the development of work-family schemes in mature welfare states. Emphasis is put on the role of collective action and corporatist institutions. I argue that two factors increase unions’ likelihood to promote the expansion of work-family policies: the relative gender composition of their membership, and institutional access to social policy-making. Using descriptive statistics and time-series cross-sectional (TSCS) designs, I find that unions promote the expansion of work-family policies when they are gender-inclusive and when female workers are able to influence public policies via corporatist institutions. In other words, both a logic of membership (female vs. male members) and a logic of influence (centralization and routine involvement in reform processes) are conducive to unions’ constructive attitude in the process of welfare recalibration. To assess unions’ role in family policy reform, I adopt the analytical lenses of Social Investment (SI), a relatively recent policy paradigm aimed at reinforcing human capital, increasing workers’ productivity and facilitating female employment through a better reconciliation of work and family life (Hemerijck, 2017; Plavgo & Hemerijck, 2021).

The mere association of these two sets of factors with work-family policy generosity does not however explain how dots are connected. Drawing from the literature and in light of new evidence, I posit that, in the context of rising female employment, trade unions provide a channel through which structural pressures affect WFP investments. This comes from both associational and institutional processes. On the one hand, decreasing gender gaps in union membership foster a slow but progressive transformation in unions’ structure and agenda. As the share of female membership increases along with labour market participation, these demands become more relevant within the organization. More women become politically active, favouring an agenda shift towards work-family reconciliation. As a result, unions feel compelled to promote diverse interests, adding gender equality to the more traditional dimension of economic solidarity (Bergholm & Sippola, 2022; Tsarouhas, 2011).

On the other hand, when available, new labour market groups within trade unions can use institutional resources (tripartite institutions, social security boards, bilateral agencies, etc.) and leverage on governments to favour social policy change in the desired direction (Skorge & Rasmussen, 2022). A higher degree of centralization and influence makes sure that demands from new social groups are incorporated in the interest formation process, and that unions shy away from the consolidation of particularistic rent-seeking strategies (Rathgeb, 2018). Far-sighted leaderships prioritize work-family policies to attract female workers and reach beyond traditional constituencies (Bledow, 2021). From this vantage point, trade unions appear not as ‘opponents’, but as ‘consenters’ if not tout-court ‘protagonists’ in the process of welfare state reconfiguration (Korpi, 2006). Reconciling the old working class with new interests, labour might form a central block in institutional coalitions that push for social investment reforms (Durazzi & Geyer, 2022; Häusermann & Palier, 2017).

In the past decades, various studies have put actors centre-stage in the process of WFP development. However, they rarely offer convincing accounts on unions’ role and their adaptation to new socio-economic circumstances. Studies centred in the influence of the labour movement overlook that other groups, such as employers’ associations and right-wing parties, might also be interested in supporting these schemes. By contrast, while correctly interpreting WFP as a salient issue all across the spectrum, the scholarship on electoral politics often dismisses unions’ role as merely obstructive, failing to detect slow but relentless changes in union structures. My analysis suggests that, due to these shortcomings, ‘left-partisanship’ and ‘electoral competition’ approaches find only partial confirmation in the empirical evidence at hand.

The article makes two contributions. First, I examine organizational change within unions in the post-industrial era, and its significance for social policy-making. The piece takes distance from narrow rational choice approaches, which tend to describe trade unions as rent-seekers opposing welfare recalibration. Rather, a dynamic perspective is adopted to show how unions adjust to socio-economic transformations. While structural factors such as rising female employment certainly encourage policy-makers to invest in WFP provisions, trade union inclusivity and institutionalization offer indirect channels for their extension. Secondly, I reassess common claims about the role of political actors in the expansion of work-family policies, pointing out that assumptions by left-partisanship and electoral competition theories are not necessarily in line with empirical applications. The article proceeds as follows: in the next section, I review recent studies on the drivers of family policy recalibration. Secondly, I propose a new theoretical framework on unions’ role in the expansion of work-family policies. After describing the data and methods used, I review the main findings and draw some conclusions.

## Women, collective action and the welfare state

In the industrial era, trade unions had a central role in welfare state expansion (Korpi, 1983). By means of a political exchange with governments, wage restraint and acquiescence were converted into protective measures against old-age poverty, sickness and unemployment (Pizzorno, 1978). Starting from the 1970s, however, industrial and socio-demographic changes have urged a restructuration of welfare systems (Hemerijck, 2013). The tremendous increase in female employment rates, service sector growth and skill-biased technological change were associated with the fragmentation of working profiles and their related demands (Bonoli & Natali, 2012). Whereas the ‘Golden age’ of the welfare state was centred in the (male) industrial working class, in the ‘Silver age’ countries have tried to recast welfare provision towards labour market newcomers, such as women, single parents, atypical workers, young people and migrants (Bonoli, 2005). The so-called new social risks (NSRs) to which these groups are exposed include work-family reconciliation issues, early-age poverty and long-term unemployment.

Work-family policies represent a central piece in the process of welfare state recalibration. This term usually refers to measures aimed at easing the reconciliation of work and family time, increasing fertility rates, boosting female participation, and not least encouraging a fair division of formal and informal care responsibilities within the household (Saraceno, 2018). Since young mothers tend to exit the labour market more easily than fathers, these measures serve the purpose of reducing care and work imbalances across gender lines, either via a more equitable distribution of caring time within the household (parental leaves) or by externalizing these tasks to third parties (childcare). In this paper, I focus on one type of work-family measures: leaves for young mothers and parents. Assessing unions’ impact on childcare systems is beyond the scope of this study, not only due to the limited availability of data, but also because unions arguably have a less influential role to play in this field (as the link with paid employment is less direct).

In the last four decades, women have rapidly entered the formal economy, concentrating especially in the public, socio-cultural and services sectors (Esping-Andersen, 1990; Oesch, 2006). As a result of the ‘second demographic transition’ (Lesthaeghe, 2014), rising divorce rates and changing perceptions on women’s role in society have spurred a substantial effort to break away from the traditional breadwinner or ‘family wage’ model, which underpins a sharp separation between the men’s function of main income recipient and women’s role as a manager of the private household sphere (Inglehart & Norris, 2000; Iversen & Rosenbluth, 2006). New attitudes to individual autonomy and family arrangements tend to be associated with declining fertility rates and rising female employment. However, the unfolding of family policy over time did not strictly follow structural changes such as low fertility and workforce diversification. Whereas different policy responses to female employment certainly mirror industrial and demographic trends, scholars have increasingly investigated other factors that explain WFP variation within and across countries.

A consistent strand of literature puts *political actors* centre-stage in process of family policy recalibration (Beramendi *et al*., 2015; Fleckenstein & Lee, 2020; Fleckenstein & Seeleib-Kaiser, 2011; Hemerijck, 2017; Morgan, 2013). Two main approaches can be distinguished. First, drawing from a power resource tradition, some scholars agree on the importance of left-wing coalitions in promoting women’s rights, thereby contributing to a ‘silent revolution’ in the welfare state (Ferragina & Seeleib-Kaiser, 2015; Fleckenstein & Lee, 2014). It is argued that women’s advocates within left-wing organizations have been crucial for the setup of WFP institutions. Women tend to vote for the left, cultivate egalitarian values, and support welfare state expansion. With a natural interest in social solidarity, labour organizations are thus expected to champion women’s claims (Morgan, 2013). Already in the 1960s, feminist movements sided with social-democratic parties to extend welfare state coverage to the emerging needs. Likewise, women’s associations acted as a ‘thorn in the flesh’ for trade unions, helping to restructure their identity and agenda towards employment and wage equality (Tsarouhas, 2011; Visser, 2019). This trend became even more prominent in the post-industrial phase, as unions were forced to go beyond traditional constituencies and recruit new members in atypical female-dominated sectors (Munro, 1999).

In line with this approach, some have shown how the presence of ‘encompassing’ and centralized producer groups leads to more egalitarian social provisions (Rathgeb, 2018; Thelen, 2014). Since Olson’s field-shaping contribution (1965), ‘encompassingness’ is seen as the ability to take up not only the interests of core workers (such as manual factory employees), but also broader societal goals. In history, unions have always acted not only as ‘vested interests’ but also as ‘swords of justice’, carrying out collective action beyond mere utilitarianism (Flanders, 1970). The concept of encompassingness is mirrored by that of ‘centralization’, that measures the extent to which interest groups are ‘vertically’ structured and aggregated. In centralized labour movements, so-called ‘peak level’ or national umbrella organizations retain most influence on strategic choices, such as wage bargaining, social concertation with governments and employers’ associations. The opposite is true for decentralized unions, where key decisions are usually taken by organizations at the sectoral or firm level (Gumbrell-McCormick & Hyman, 2013).

As unions federate new sectors to reverse their secular decline, labour’s agenda is revised to accommodate new and distinctive interests. For instance, a critical mass of women within unions, centralization and institutional influence were key factors behind Sweden’s shift to the double-earner model (Fleckenstein & Lee, 2014; Tsarouhas, 2011). Initially, the Swedish blue-collar organization LO framed the concept of solidarity largely along class lines. As more women entered the labour market, however, LO’s ideological repertoire was renewed to account for female employment. Emphasizing that women were ‘doubly disadvantaged’ by both class and gender, female policy entrepreneurs gradually incorporated gender equality demands into the union’s priorities. This commitment was framed as consistent with the emerging economic model and transition to a ‘social service state’ (Huber & Stephens, 2001). Close ties with the social-democratic party SAP and a legacy of concertation made it possible to spur change in the policy-making sphere. Likewise, Sweden saw the early rise of white collar, professional and academic sector unions (TCO and SACO) which have historically been vocal on issues such as equal pay, separate taxation and work-family policies (Qvist, 1980). Similar dynamics have been reported in Norwegian, Danish and to a lesser extent British unions (Fleckenstein & Seeleib-Kaiser, 2011; Skorge & Rasmussen, 2022).

In the last two decades, however, theoretical expectations derived by left-partisanship approaches have failed to match with empirical observations. Left-partisanship arguments have been challenged on two main grounds. First, a burgeoning scholarship emphasized that employers can also be *protagonists* in the development of welfare and family policy regimes (Swenson, 2003). Employers’ support for generous leaves and early childhood education and care (ECEC) policies has been justified with increasing women’s participation rates and labour productivity (Skorge & Rasmussen, 2022). In Germany, for instance, business associations have strongly advocated for a shift towards a dual-earner model of family provision via generous childcare and parental leaves, while unions have been at best lukewarm towards these attempts (Seeleib-Kaiser, 2016). The more women enter higher education and increase their skillsets, the more employers will try to make sure they can balance work and family commitments. This analytical perspective suggests that, rather than the result of labour’s struggle, ‘new’ social policies are in fact the fruit of cross-class compromise between moderate union confederations on the one hand, and pragmatic employers’ associations on the other, for the sake of adapting their countries to the knowledge economy (Durazzi & Geyer, 2022; Ornston, 2013).

However, where the left-partisanship-*cum-feminism* argument shows its most evident limitation is on the electoral competition side. Left-partisanship scholars assume that female voters have natural elective affinities with left-of-centre parties, which in turn find it convenient to embrace their demands. In the last decades, though, deindustrialization and cultural changes have radically altered party-vote dynamics (Beramendi *et al*., 2015; Gingrich & Häusermann, 2015). Whereas the left-partisanship argument is robust for a limited number of pioneering countries (Nordic *in primis*), in latecomer countries such as Continental and Mediterranean welfare states the root causes of WFP expansion are likely to be found elsewhere (Fleckenstein & Lee, 2020). A crucial finding of the literature is that both large-N and case study research fail to identify significant differences between left and right parties on the promotion of female employment policies (Ferragina & Seeleib-Kaiser, 2015; Lambert, 2008). Instead, Morgan (2013) explains WFP development with a peculiar sequence of events, including both structural and political factors: first, (male-dominated) parties recognize the electoral potential of women, whose preferences become salient as a result of low participation or fertility rates; consequently, male party authorities increase women’s role within their organizations; finally, female party leaders and MPs push WFP reforms based on both electoral *and* normative considerations.

Electoral competition scholars question the primacy of left parties and trade unions in defending women’s interests. The withering of partisan effects on family policy reforms since the 1980s left room for political entrepreneurs in search of new constituencies (Gingrich & Häusermann, 2015). Actors from all across the spectrum have tried to address new segments such as low-end service workers and socio-cultural professionals (Häusermann & Palier, 2017). As female employment rates and electoral participation increase, centre-right parties have used the rising popularity of WFP to craft novel political coalitions (Häusermann, 2018). In the UK, Germany and the Netherlands, moderate or right-wing parties have profited from the social-democrats’ delay on work-family issues to capture women’s votes (Morgan, 2013). Moreover, compared to the left-partisanship literature, the electoral competition approach provides radically different expectations on trade unions’ role in family policy development. Rather than a ‘sword of justice’ for female workers, labour is seen as anchored in the old, male industrial working class and thus uninterested in reforming welfare states in the advantage of young mothers (Häusermann, 2010). If anything, union influence is expected to be detrimental to women’s emancipation and the transition to social investment welfare states (Beramendi *et al*., 2015).

## Towards a synthetic understanding of unions’ role in WFP expansion

Studies on left-partisanship and electoral competition have placed substantive emphasis on the role of actors and their interest-based behaviour. Both approaches focus on how parties and producer groups respond to post-industrial challenges, either due to shifts in composition, attempts to recruit new members or the influence of feminist advocates. However, both perspectives fail to provide a comprehensive account on the politics of WFP reform. On the one hand, left-partisanship approaches are ill-suited to explain family policy recalibration where the left is not, or no longer, in power. On the other, electoral competition theories dedicate limited attention to actors besides political parties. When included in the analysis, unions’ preferences are deemed to be homogeneous, stable and stubbornly insider-biased (Bledow, 2021; Tassinari *et al*., 2022). This contrasts with empirical evidence outlining unions’ slow but relentless adaptation to labour market feminization, as well as a wide variety of positions towards social investment (Bledow & Busemeyer, 2021; Clegg & van Wijnbergen, 2011; Hassel, 2015; Naczyk & Seeleib-Kaiser, 2015). In what follows, I go beyond existing explanations by detailing the dynamics of organizational change, and notably how associational and institutional factors inform union strategies in the context of family policy recalibration.

A recent article by Skorge and Rasmussen (2022) tries to go beyond party-centred explanations and relocate the issue of WFP expansion in the broader transition to the knowledge economy. The authors hypothesize that women’s entrance in higher education, union inclusivity and corporatist institutions are conducive to investments in families and children. Using time-series fixed-effects models, they find that female educational attainment, centralized producer groups and female membership rates are positively associated with parental leave generosity. Whereas the study makes an important addition to the literature, different trends are juxtaposed without clearly detailing the intersection between actors’ power resources and social policy orientations. First, they use a synthetic index as a proxy for corporatist influence, which assimilates social partners centralization with their involvement in policy-making. While this is a good indicator of labour inclusivity and dialogue with business and the state, these two dimensions capture different dynamics and therefore could be analytically distinguished. In addition, the analysis starts from 1960, conflating the idiosyncratic logic of the Fordist political exchange with post-industrial and knowledge-economy strategies. Finally, they only use parental leave generosity as a dependent variable, overlooking maternal and childcare leave schemes as drivers of work-family reconciliation.

My article proposes a more fine-grained description of the shifts within labour, and how these are converted into social policy strategies. It argues that associational (‘membership’) and institutional (‘influence’) factors encourage unions to promote WFP reforms. I start by assuming that, akin to social-democratic parties (and political parties in general), unions adapt to the post-industrial age by renewing their membership pools. Since stronghold sectors are in decline, survival can be guaranteed only by recruiting in new service sectors where female workers are overrepresented (Pulignano *et al*., 2016). Higher female-to-male membership ratios are connected with shifting welfare preferences in two main ways. First, an increasing number of female against male members broadens the range of interests within labour. This dynamic rests on the traditional ‘logic of membership’ (Schmitter & Streeck, 1999). As more women join unions, the realization that this type of policies may be important is spread within the organization. Leaders have incentives to take up WFP demands, and couple them with more traditional issues such as social security. Second, as women and feminist activists gradually climb the organizational ladder, they become leaders themselves, thereby pushing for the inclusion of WFP into the agenda. These social policy preferences make it to the top of labour organizations, that devise new strategies in view of responding to a wider set of labour market needs (Morgan, 2013).

Besides such associational factors, unions’ institutional features may also provide key leverage to labour market newcomers, allowing them to convert their claims into legal prescriptions or reforms. Here comes into play the second logic of ‘influence’, that is, strategies aimed at prioritizing the organization and its survival (Schmitter & Streeck, 1999). In the context of WFP reform, institutional power resources serve a twofold role. First, union leaders generally need to account for future goals and take decisions that have long-term consequences (Berger, 1983; Jacobs, 2011). The extent to which union leaders are insulated from their members (i.e., do not need to respond to here-and-now material concerns) allows them to legitimately interpret what is *best* for the organization, that is, their ‘true’ long-term interests (Offe & Wiesenthal, 1985). Far-sighted leaders who enjoy some degree of autonomy from the grassroots base promote social investments with the aim to attract, and potentially benefit, female workers, redefining narrow material interests into more encompassing policy goals (Bledow, 2021). Leaders of centralized organizations with routine access to policy-making can exert pressure on governments in the area of WFP to enhance their legitimacy in front of, and possibly recruit, new female members. Second, union centralization forces different groups within the union movement to find a compromise, thereby creating opportunities for female workers to influence the overall strategy (Rathgeb, 2018).

Associational and institutional factors also reflect different forms of *strategic capacity* (Hyman, 2007). I draw from Hyman (2007, p. 198) to define strategies as ‘procedures and traditions which link knowledge to action through analysis of circumstances, evaluation of alternative options and planning of objectives and forms of intervention’. Strategic capacity, on the other hand, can be seen as the ‘attainability of union policies within the objective context’; that is, unions’ actual possibilities to push their strategies through, given political and institutional constraints. In the context of welfare recalibration, unions rely on two forms of strategic capacity. First, encompassing unions have *associational power* to mobilize popular consent in favour of certain policies. They can use the ‘strength in numbers’ (e.g., voicing their positions through the media, protests, industrial action) and legitimacy deriving from the larger participation of female members to encourage welfare innovation (Doellgast *et al*., 2018). Second, they count on *institutional power* via platforms such as tripartite boards, informal policy-making practices and ties with social-democratic parties. In corporatist contexts, centralized and influential producer groups transfer their claims via ‘interlocking directorates’ to the parliament and parties in government (Berger, 1983).

While most CPE accounts assume that unions engage with governments to obtain gains on behalf of traditional workers, I argue that both ‘associational’ and ‘institutional’ logics steer unions towards the defence of NSR groups. On the one hand, membership diversity at the grassroots and leadership levels stimulates unions to modernize their agenda. Rather than ‘overturn[ing] all their past definitions of character and purpose’, unions experiment, learn and ‘adapt selectively’ to the political economic environment, including a sharp increase in female participation (Hyman, 2007, p. 198). Second, centralized unions with formal access to policy-making are more likely to empower marginal groups, as well as to have enough leverage to put WFP proposals centre-stage in the process of political exchange (Pizzorno, 1978). In the context of rising female employment and declining fertility, unions act not as obstructive ‘antagonists’ to welfare recalibration, monopolized by sectarian factions within the working class, but rather as ‘consenters’ if not *tout court* ‘protagonists’ in reforms that favour female employment and an equitable distribution of work-family duties (Korpi, 2006). My hypotheses are therefore that:

H1: Larger proportions of female-to-male union members are positively associated with the expansion of work-family policies (such as leaves).

H2: Union centralisation and access to corporatist institutions are positively associated with the expansion of work-family policies.

Moreover, unions’ role in WFP reform is likely to depend on different welfare state and trade union types. Nordic, Liberal, Continental or Mediterranean welfare regimes present distinct features, both in terms of institutional arrangements and the degree to which different socio-economic needs are satisfied (Esping-Andersen, 1990). Nordic systems have transitioned into a dual-earner model much earlier than other countries, while Southern European ones in many ways lag behind. Likewise, trade unions differ in their cultures, power resources and organizational features, with non-negligible implications on their preferences towards welfare recalibration (Gumbrell-McCormick & Hyman, 2013). Albeit such differences are not discernible in this study, organizations who fall closer to a tradition of ‘business’ unionism generally focus on the narrow interests of their members, while those nearer to ‘class’ and ‘societal’ unions styles account for a broader set of needs (Hyman, 2001). Differences also a rise on the degree of institutionalization, as unions in Nordic and Continental countries usually benefit from routine participation in political decisions as compared to Liberal and Southern European countries.

In the following section, I illustrate the main variables, describe their operationalization and the estimation strategy, before passing to the empirical results.

## Data and methods

To test these dimensions empirically, I gather data on 20 OECD countries in the 1980–2010 period. Male and female union density^1^ rates are extracted from the ICTWSS database (Visser, 2019). Density rates across women and men are used to calculate *gender odds ratios*, which I consider as a proxy for union inclusivity (Table 1). If H1 is true, in the context of declining fertility and rising female employment rates, higher gender odds ratios should be associated with the generosity of work-family policies, such as different types of leaves. I complement this indicator with two measures of unions’ participation in the state structure from Visser (2019). The first variable illustrates the routine involvement of unions and employers in government decisions on social and economic policy, ranging from 0 (no concertation) to 2 (full concertation). Secondly, I add a synthetic measure of centralization to verify the capacity of peripheral groups to participate in strategic decisions. H2 expects that centralization and routine involvement favour the extension of WFP schemes.

**Table 1.** Centralization, routine involvement in policy-making and female-to-male membership ratios by country (*N* = 20) and welfare cluster across different periods (Visser, 2019).

		Centralization		Inv. in policy-making		Female share	
		1985–1990	2005–2010	1990	2010	1985–1990	2005–2010
**Nordic**	Denmark	0.80	0.76	2.00	2.00	0.98	1.07
	Finland	0.67	0.70	1.00	1.00	1.03	1.14
	Norway	0.80	0.83	2.00	2.00	-	1.30
	Sweden	0.74	0.70	2.00	2.00	1.03	1.08
	***Mean***	***0.74***	***0.75***	***1.75***	***1.75***	***1.01***	***1.13***
**Continental**	Austria	1.22	1.14	2.00	2.00	0.67	0.57
	Belgium	0.66	0.68	2.00	2.00	-	0.80
	France	0.28	0.32	1.00	0.00	-	0.88
	Germany	0.36	0.46	1.00	1.00	0.51	0.61
	Netherlands	0.77	0.87	2.00	2.00	0.38	0.62
	Switzerland	0.33	0.50	2.00	2.00	0.34	-
	***Mean***	***0.60***	***0.66***	***1.67***	***1.50***	***0.47***	***0.60***
**Southern European**	Greece	0.37	0.38	0.00	1.00	0.48	0.68
	Italy	0.38	0.39	0.00	1.00	-	0.92
	Portugal	0.47	0.44	1.00	1.00	-	-
	Spain	0.48	0.53	0.00	1.00	-	0.80
	***Mean***	***0.42***	***0.44***	***0.25***	***1.00***	***0.48***	***0.80***
**Liberal**	Australia	0.51	0.12	1.00	1.00	0.77	0.96
	Canada	0.21	0.25	0.00	0.00	0.57	1.07
	Ireland	0.43	0.49	0.00	1.00	1.05	1.14
	New Zealand	0.43	0.14	1.00	1.00	-	1.34
	United Kingdom	0.19	0.22	0.00	0.00	0.81	1.20
	United States	0.14	0.20	0.00	0.00	0.63	0.90
	***Mean***	***0.32***	***0.24***	***0.33***	***0.50***	***0.77***	***1.10***

Note: More details are available in the annex.

I test these hypotheses by looking at two dependent variables. First, I construct an index of leaves generosity based on leaves net replacement rates and duration (in weeks). I choose to focus on leaves because their function is precisely to support work-family reconciliation and female employment rates. The index is unweighted (all components have the same importance) and includes data on duration and replacement rates of maternal, parental and childcare leaves from the Comparative Family Policy Database (Gauthier, 2011). For every country and year, I first calculate an average score based on the duration of each of the three schemes (where available) and another one based on the income replacement rate of each scheme. I then compute the average of these two measures to create a synthetic leave index, ranging from a minimum of 0 to a maximum of 90.6. A more detailed explanation on construction of the index is available in the appendix.

The decision to include all three leave schemes in the index is based on the idea that, albeit each programme comes with different goals and features, they all contribute in some way to smoothening women’s transitions in and out of employment (the ‘flow’ function in a social investment jargon). To include one without considering the others (e.g., maternal without parental and childcare leaves, or vice versa) would offer an incomplete picture of how countries combine different schemes to promote work-family reconciliation and female employability. Furthermore, while high leave generosity might be seen as discouraging work participation, it still provides an indication of whether a country makes efforts in promoting investments in families and children. In any case, robustness checks with alternative dependent variables (only maternal, only parental, only maternal + parental leaves) are provided in the appendix. As an additional test, I check whether union inclusivity and institutional resources are correlated with public expenditures on leaves as a share of GDP (Armingeon *et al*., 2021). This measure, more common in the literature, is meant to triangulate results from the leave generosity index.

The empirical section is divided into two parts. In the first part, I make use of descriptive statistics to see how countries ‘fare’ regarding their levels of leave generosity *vis à vis* levels of union inclusivity and incorporation in the institutional framework. This section includes data on 20 OECD countries over the whole period (1980–2010). Simple country mapping and bivariate regressions give us a first indication on whether countries with more gender-sensitive and institutionalized unions also tend to provide young parents with more generous work-family policies. In the second part, I run time-series cross-sectional analyses with data from 1990 to 2010.^2^ Both hypotheses suggest that unions provide a further channel by which structural pressures are converted into WFP reforms, to the extent that labour organizations are sufficiently gender-inclusive or institutionalized. If this reasoning is correct, adding union-related indicators to the model is expected to improve its overall explanatory power, and keep robust to the presence of fertility and female employment rates. The use of fixed-effects models is aimed at finding out whether union inclusivity and institutionalization have a significant impact on WFP expenditures and coverage, as measured by leaves generosity (or leaves spending as an additional checkup).

In line with previous studies on the relationship between union strength and welfare generosity, I use fixed-effects (Gordon, 2015; Hooghe & Oser, 2016; Swank, 2020). Fixed-effect models are meant to assess within-country variation over a certain period. This approach eliminates time-constant unobserved heterogeneity as it compares the same observations over time. Fixed-effects cannot account for time-invariant characteristics such as Ghent systems or market economy models. However, they are effective at singling out how a certain set of variables longitudinally affects the dependent variable within the countries analysed. In the context of this article, they are expected to illustrate how variations in the gender balance of trade unions and their institutionalization are associated with variations in WFP generosity. In view of addressing possible OLS violations, all models are adjusted for country-level robust standard errors. Moreover, within-country designs that measure changes between individual years run the risk of autocorrelation, that is, to inflate independent variables’ statistical and substantive significance. To check if results are due to autocorrelation, I include a lagged dependent variable in all models.

Finally, in view of testing if my hypotheses hold robust to conventional explanations, I add a number of controls, such as employer density (OECD & AIAS, 2021), log GDP per capita (OECD, 2021), government strength (Armingeon *et al*., 2021), share of female parliament representatives and left and right-wing parties cabinet shares^3^ (Brady *et al*., 2020). According to the left-partisanship literature, WFP expansion tends to be fostered by left-of-centre cabinets and, to a minor extent, trade unions. For electoral competition scholars, instead, it rests on factors such as the (higher) share of female MPs and government strength, since female-dominated parliaments and solid majority governments have more chances to push the intended reforms through. In line with the latter approach, a variable on employers’ organizations density is included to probe whether unions’ agency is independent of the role of business interests.^4^ I also control for unemployment and the share of elderly people.

Unfortunately, data on female and male density ratios, centralization and employers’ density result somewhat scattered across years. To deal with scarce information, I follow Skorge and Rasmussen (2022) in performing linear interpolation for missing observations. Differences between designs mostly seem to depend on the very low number of observations in models with non-interpolated variables, as shown in the appendix.

## Descriptive statistics

**Figure 2.**
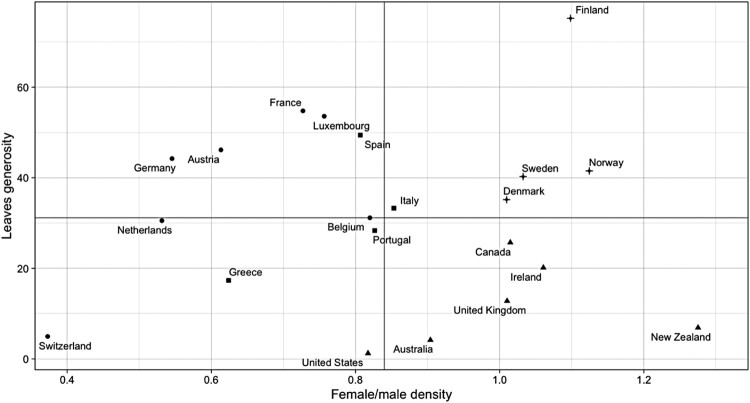
Cartesian graph mapping countries according to leaves generosity and female-to-male union density odds ratio (pooled averages 1980–2010). Black solid lines represent median values. Different dots indicate welfare regime families (Nordic; Liberal; Continental; Mediterranean).

Figure 2 plots countries’ position regarding their levels of leaves generosity and union inclusivity, as proxied by the relative subscription rate of female versus male union members. For both variables, country averages are computed across the whole period (1980–2010). Black solid lines represent median values. Whereas no clear cross-sectional trend emerges, countries seem to cluster according to distinct levels of union encompassingness and WFP extension. In the top-right quadrant, Nordic countries are characterized by high levels of leaves generosity and gender inclusivity in workers’ organizations. Since the 1980s, Nordic trade unions have been vocal promoters of the dual earner model, operating in welfare states that are generally seen as the ‘early movers’ of the post-industrial transition. In the bottom-right area, Liberal market economies feature high shares of female union membership, which stand in contrast with comparatively low levels of leaves generosity. Widespread female representation is the result of a decentralized model of union ‘organising’, in which unions strive to enhance grassroots coverage also among female-dominated sectors such as public and general services (Gumbrell-McCormick & Hyman, 2013). Conversely, in Mediterranean and Continental welfare states low levels of female union representation coexist with medium-to-high levels of leaves generosity, as extensively reported in the dualization literature.

**Figure 3.**
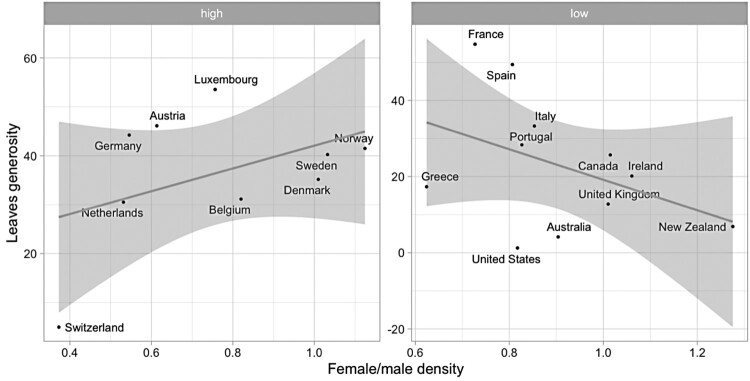
Scatterplots of countries’ leaves generosity and female/male union density ratios in different corporatist contexts (‘low’ vs. ‘high’ levels of routine involvement of trade unions in policy-making; pooled averages 1980-2010). Black solid lines indicate the best fit using OLS. ‘High’: above the median value of ‘routine involvement in policy-making’; ‘low’: below median.

My main contention is that trade unions’ inclusivity and their institutionalization encourage political exchanges with the government in the area of work-family policies. Whereas assessing interactions between the two logics is beyond the scope of this study, they could work simultaneously in certain configurations. In corporatist contexts, unions represent a potential channel through which new labour market groups find representation and encourage legislation on ‘new’ social policies. If this expectation is correct, I should be able to see that the relationship between union inclusivity and leaves generosity is more positive in contexts where unions have institutionalized access to policy-making, and can therefore engage with governments on WFP reforms, and non-existent or even negative in countries where unions cannot rely on customary participation in state structures. Figure 3 splits the previous graph into two groups: countries where unions are regularly involved in social concertation, and countries where they are not. As expected, the association between female union participation and leave generosity is positive in contexts where social partners are conventionally invited to negotiate on social policy issues. On the other hand, Liberal and Mediterranean countries (including France) show a negative association between the two variables. Southern European economies offer more generous leave provisions than one would expect by looking at their degrees of union inclusivity, whilst more gender-inclusive Liberal unions coincide with low degrees of statutory WFP coverage.

**Figure 4.**
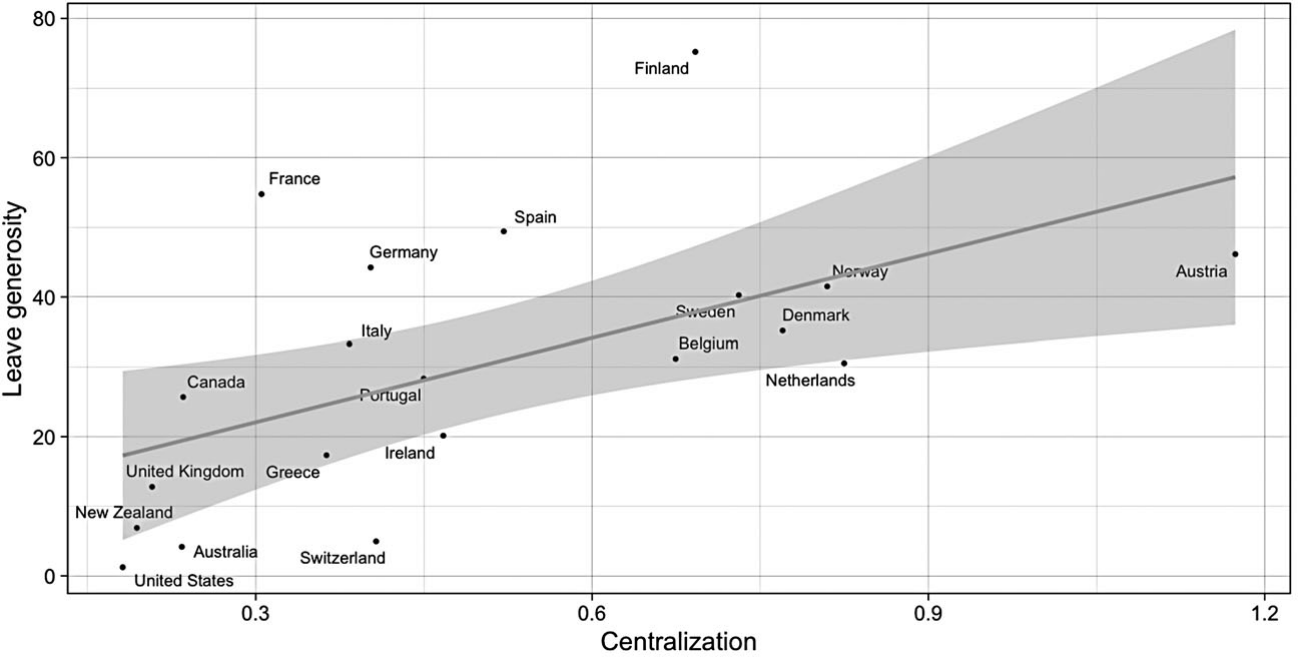
Scatterplot of leaves generosity and union centralization (pooled averages 1980–2010). The black solid line indicates the best fit using OLS.

Finally, the relationship between wage bargaining centralization and leaves generosity is displayed in Figure 4. As made clear above, centralization resolves crucial collective action problems within the union movement by empowering peripheral labour market segments. In centralized unions, NSR groups such as female workers have greater leverage on the peak-level agenda, thereby increasing their chances to influence social policies in the desired direction. Unsurprisingly, the association between the two variables is positive: higher degrees of union centralization are correlated with larger social investments in young parents and children. In this case again, Liberal and Mediterranean market economies tend to cluster around the bottom-left area of the graph, while Continental (Belgium, Netherlands, Austria) and Nordic countries (Sweden, Denmark, Norway) are located on the right-hand side. The most apparent exceptions are France, Germany and Spain. In the face of relatively generous leave provisions, producer groups in these countries are only moderately centralized. Another outlier is Finland, whose statutory provision of parental, maternal and childcare leaves is far more generous than one would predict by its degree of labour centralization.

## TCSC analysis: findings from fixed-effects estimations

Regression results confirm that gender inclusivity in unions is positively correlated with the expansion of WFP regimes. In fixed-effects specifications, the more the union gender balance is skewed towards women, the more governments tend to invest in policies that enhance female employability (Table 2). For every additional percentage point in the share of female versus male members, the leave generosity index increases by 5.1–9.1 points. Consistently with my H1, countries where more women join trade unions tend to make larger investments in maternal, parental and childcare leaves. As discussed in earlier sections, this is the result of pressures from the grassroots base as well as from higher leadership positions. As trade unions move away from their male breadwinner bias, social policy strategies are updated accordingly. This finding holds robust to the main structural variable (female employment rates), which is highly significant and positively correlated, as well as fertility, which fails to reach significance levels. This result is confirmed also when taking leave expenditures on the dependent variable: female-to-male union density is statistically significant and positively associated with leave spending in the full model, and robust to a series of demographic, economic and institutional controls.

**Table 2.** Regression results from fixed-effects designs, including lagged dependent variables and country-clustered robust standard errors (in parentheses).

	Leave generosity		Leaves spending (% GDP)	
	(1)	(2)	(3)	(4)
Lagged dep. var.	0.734***	0.715***	0.854***	0.853***
	(0.037)	(0.043)	(0.036)	(0.036)
Female-male ratio	5.143*	9.138**	0.001	0.048**
	(3.042)	(4.323)	(0.020)	(0.022)
Routine involvem.	−0.054	0.379	0.013*	0.015*
	(0.912)	(0.883)	(0.007)	(0.008)
Centralization	0.136	0.111	−0.070	−0.119
	(4.956)	(4.305)	(0.107)	(0.126)
Employers’ density	0.058	0.100	−0.00005	0.001*
	(0.094)	(0.097)	(0.001)	(0.001)
Female employment	0.211***	0.224***	0.001	0.001
	(0.078)	(0.074)	(0.001)	(0.001)
Fertility rate	−1.470	−1.358	0.060	0.055
	(1.596)	(1.657)	(0.044)	(0.041)
Left cab. Share	−0.003	−0.005	−0.00001	−0.00002
	(0.006)	(0.006)	(0.0001)	(0.0001)
Right cab. Share	−0.002	−0.003	0.0001	0.00004
	(0.005)	(0.005)	(0.0001)	(0.0001)
Women in parliam.	−0.026	0.009	−0.0002	0.001
	(0.023)	(0.034)	(0.0004)	(0.001)
Govern. Strength	0.621	0.490	−0.002	−0.007
	(0.502)	(0.460)	(0.008)	(0.007)
Elderly population		0.345*		0.008***
		(0.208)		(0.002)
Unemployment		0.036		−0.003**
		(0.054)		(0.001)
Log GDP per capita		−2.134		−0.065***
		(1.483)		(0.019)
Observations	414	414	521	521
R^2^	0.748	0.752	0.781	0.792
Adjusted R^2^	0.728	0.731	0.768	0.778
F Statistic	103.368*** (df = 11; 383)	82.384*** (df = 14; 380)	159.513*** (df = 11; 491)	132.482*** (df = 14; 488)

**p < 0.1;**p < 0.05;***p < 0.01.

Variables that gauge unions’ institutional resources and access to reforms (H2) only partially act as anticipated. Following classical corporatist accounts (Schmitter, 1974), I expect that centralized interests allow more peripheral groups to influence reform choices. Corporatism can also be seen as the *practice* of inviting relevant actors to the decision-making process, as captured by the ‘routine involvement’ variable (Lembruch, 1984). In countries where unions are centralized and recognized as relevant partners, new labour market groups have higher chances to influence governments on social policies. However, this hypothesis finds only moderate confirmation: when coming to the empirical results, the degree of routine involvement in policy-making is significant and positively correlated with spending on leave schemes (with every additional unit increasing leaves expenditures by 1.3–1.5 per cent), but not with the leave generosity index. Despite showing a positive association with leave generosity, the centralization variable is not significant in fixed-effects specifications. The reason may be that there is less variation in corporatist practices over time within countries, which in turn hinders the explanatory power of these factors on the selected response variables. Albeit not reaching significance in three models over four, employer density positively correlates with WFP generosity. This finding is in line with the argument by Skorge and Rasmussen (2022) that business interests favour the expansion of WFP provision to enhance female employment and productivity.

The remaining variables in the model offer limited evidence in favour of alternative actor-centred explanations (electoral competition and left partisanship). Differently from what is postulated by electoral competition scholars, the share of female MPs seems to have neither a substantial nor a statistically significant effect on leaves generosity. The same applies to the share of left and right-wing cabinets: I fail to see any clear left-partisan trend on the expansion of work-family policies. Effects are negligible in absolute terms and non-significant. The issue seems to be less controversial or politically connotated than a left-partisanship approach would expect. On the other hand, electoral competition studies have well documented how post-industrial cleavages spur both left and right parties to attract female voters, and that centre-right actors can take important reform initiatives in this domain (Morgan, 2013). This would corroborate the claim that in the post-industrial era left parties ceased to be the sole champions of labour market newcomers, including women. The share of elderly population is positively associated with leave investments. Finally, unemployment is negatively and significantly correlated with leave expenditures: one potential reason may be that, as unemployment goes up, spending on shock absorbers also surges, in turn crowding out expenditures on other policy areas.

## Discussion and conclusion

In much of the recent literature, there is a shared understanding that unions have missed the train of post-industrialization, resorting to the protection of core interests (Emmenegger *et al*., 2012; Palier & Thelen, 2010). Smaller and weaker than in the past, unions would act as mere lobbies on behalf of traditional members, disregarding new economic groups and their needs. Yet, such conclusions might have been too quick, failing to grasp deep-seated trends within labour. Despite popular perceptions of unions as ‘vested interests’, unions have always pushed their demands beyond narrow membership (Flanders, 1970). In the last decades, unions have made efforts to go recruit in new, female-dominated service sectors, and reconfigure their practices to a much more diverse set of working profiles (Doellgast *et al*., 2018; Gumbrell-McCormick & Hyman, 2013). In the political arena, they have tried to turn the ‘snakes’ into ‘ladders’ (Crouch, 2000), supporting governments in the transition to women-friendly welfare states. Contrary to the view of unions as antagonists to welfare recalibration, this article suggests that, under certain organizational and institutional conditions, labour may join coalitions that promote social investment reforms (Bledow, 2021; Durazzi & Geyer, 2022; Häusermann & Palier, 2017)

This study makes two contributions to the literature on producer groups and welfare recalibration. First, I corroborate previous arguments on trade union revitalization, suggesting that labour is indeed adapting to new socio-economic realities. In particular, I shed light on the importance of membership inclusivity (associational resources) and corporatist channels (institutional resources) for the reconversion of unions from defenders of the *status quo* to champions of new economic segments. Findings validate the claim that gender-inclusive unions and those that enjoy higher degrees of institutionalization are associated with the expansion of WFP regimes since the 1980s. With the transition to the knowledge economy and dual-earner labour markets, women and their movements have infiltrated into the gears of unions, managing to impose new priorities in the agenda and modify their long-term strategic orientations. New grassroots interests to be represented and a higher share of women at the top of these organizations concur in reshaping unions’ priorities towards welfare recalibration. At the same time, peripheral groups are likely to have more influence when the union movement is centralized and institutionally embedded. Whereas centralization encourages unions to broaden their agenda towards a wider set of demands, corporatist involvement offers new social groups a more direct channel to WFP formation.

Secondly, I reassess left-partisanship and electoral competition explanations on family policy investments, finding only moderate confirmation for their claims. The positive relationship between union inclusivity and WFP generosity corroborates the left-partisanship hypothesis: as labour organizations slowly adapt to labour market feminization, their functions, priorities and strategies are revised accordingly. Centralized union movements are best placed to assimilate the demands of labour market outsiders and steer social policy-making in their favoured direction. However, contrarily to left-partisanship assumptions, I find no significant left-partisan effect on leaves generosity. This rather seems to confirm electoral competition expectations that both left and right parties are interested in the expansion of WFP packages. At the same time, studies emphasizing the role of electoral entrepreneurs also raise the attention on factors such as the share of female MPs, whose effect is not significant in any of my models. More importantly, electoral competition claims that trade unions are uninterested in NSRs are rebutted in the light of this new evidence. Looking at the dynamics of organizational change within unions over time, the article attests that higher degrees of gender inclusivity and institutionalization spur unions to support work-family reconciliation.

Besides paying attention to ‘thick’ material interests, future research should also explore ideational processes within unions, and their effect on welfare state reconfiguration. Negotiations in the context of welfare reform do not happen in a vacuum: unions’ ideological repertoires and identities are continuously rediscovered, acting as cues or ‘road maps’ in situations of extreme uncertainty. These causal beliefs are likely to have a strong influence on how gender equality and work-family reconciliation are processed by union élites. Despite having material bases, new concepts can assume a separate normative dimension, giving shape and purpose to unions’ strategies (Cigna, 2022; Tassinari *et al*., 2022). Future studies may look at how such shifts in normative orientations affect the ‘explicit negotiation of increasingly overt internal differences’ between groups and their priorities within the labour movement (Hyman, 2007, p. 206). Likewise, whereas ‘influence’ and ‘membership’ channels have been analysed as independent from each other, future accounts should assess whether these two logics interact, and how this affects the politics of social policy change.

## Supplementary Material

Supplemental Material

## Notes

1 Union density is usually defined as the net share of union members as a proportion of wage earners (Visser, 2019).

2 I am forced to use a shorter time period as OECD time series on female employment rates start from year 1990.

3 Right-wing cabinets comprehend both secular and catholic right.

4 I use this variable as a proxy for employer associations’ representativity in the labour market. Their influence on policy-making is already captured by the routine involvement variable.
