# Supplementary material for: Bread or roses? Trade unions, female employment and the expansion of work-family policies
Source: J Eur Public Policy. 2023 Mar 6;31(5):1320–45. doi: 10.1080/13501763.2023.2184414 (PMC10964190; doi:10.1080/13501763.2023.2184414)
Supplement: Supplemental Material [file RJPP_A_2184414_SM2065.docx]

1. Variables description.

|  | **Variable name** | **Description** | **Source** | **min** | **max** | **mean** | **s. dev.** | **obs.** |
| --- | --- | --- | --- | --- | --- | --- | --- | --- |
| **Dependent**  **variables** | MLWKS | Total number of weeks of maternity leave | Gauthier, 2011 | 0 | 100 | 13,76 | 13,73 | 655 |
|  | MLPAY | Cash benefits paid during maternal leave (as a percent of female wages in manufacturing) | Gauthier, 2011 | 0 | 100 | 55,60 | 39,35 | 670 |
|  | PLWKS | Total number of weeks of parental leave | Gauthier, 2011 | 0 | 156 | 39,18 | 47,84 | 681 |
|  | PLPAY | Cash benefits paid during parental leave (as a percent of female wages in manufacturing) | Gauthier, 2011 | 0 | 105,72 | 23,60 | 32,85 | 681 |
|  | CCWKS | Total number of weeks of childcare leave | Gauthier, 2011 | 0 | 156 | 18,99 | 39,95 | 681 |
|  | CCPAY | Cash benefits paid during childcare leave (as a percent of female wages in manufacturing) | Gauthier, 2011 | 0 | 72 | 3,68 | 10,33 | 671 |
|  | COMPWKS | Average of leaves duration (maternal, parental, childcare leave) | Gauthier, 2011, own elaboration | 0 | 93,8 | 24,99 | 22,97 | 681 |
|  | COMPPAY | Average of replacement rates (maternal, parental, childcare leave) | Gauthier, 2011, own elaboration | 0 | 77,95 | 27,49 | 17,02 | 681 |
|  | LVGEN | Composite index of leaves generosity (average of normalized COMPWKS and COMPPAY) | Gauthier, 2011, own elaboration | 0 | 90,62 | 30,96 | 20,76 | 681 |
|  | MPLEAVE | Total public and mandatory private cash benefits for maternal and parental leave as a percentage of GDP | Armingeon et al., 2021 | 0 | 1,64 | 0,30 | 0,27 | 961 |
| **Independent**  **variables** | FMUD | Odds ratio between union density rate of females and union density rate of males (union density: net union membership as a proportion of wage and salary earners in employment) | Visser, 2019, own elaboration | 0,31 | 2,12 | 0,91 | 0,30 | 408 |
|  | FMUD2 | Interpolated values for the same variable as above | own elaboration | 0,31 | 2,12 | 0,94 | 0,30 | 1200 |
|  | CENT | Summary measure of centralization of wage bargaining | Visser, 2019 | 0,08 | 1,22 | 0,53 | 0,27 | 779 |
|  | CENT2 | Interpolated values for the same variable as above | own elaboration | 0,08 | 1,22 | 0,50 | 0,27 | 960 |
|  | RIV | routine involvement of unions and employers in government decisions on social and economic policy | Visser, 2019 | 0 | 1 | 0,49 | 0,40 | 1047 |
|  | EMPDOECD | Employer organizations’ density (share of employees in firms organized by employers’ associations). For the US, employer density in the private sector is used | OECD-AIAS, 2021 | 0 | 100 | 0,67 | 0,16 | 198 |
|  | EMPD2 | Interpolated values for the same variable as above | own elaboration | 0 | 100 | 0,63 | 0,19 | 1159 |
| **Controls** | GDPCAP | Share of GDP per capita | OECD, 2021 | 5504,27 | 120670,50 | 28150,73 | 16279,98 | 1114 |
|  | FERRATE | Number of children born per woman | OECD, 2021 | 1,1 | 3,2 | 1,67 | 0,29 | 1175 |
|  | FEMP_OECD | Share of employed people as a percentage of the female labour force | OECD, 2021 | 33,30 | 78,20 | 54,83 | 8,68 | 863 |
|  | LEFTCAB | Share of seats in parliament held by leftist parties in the most recent government as a percentage of all seats held by the government | Brady et al., 2020 | 0 | 100 | 35,54 | 39,51 | 798 |
|  | RIGHTCAB | Share of seats in parliament held by right parties in the most recent government as a percentage of all seats held by the government (sum of catholic and secular right parties) | Brady et al., 2020 | 0 | 100 | 39,04 | 40,40 | 836 |
|  | UNEMR | Share of unemployed people as a percentage of the civilian labour force | Brady et al., 2020 | 0,185 | 27,47 | 7,41 | 4,25 | 836 |
|  | ELDPOP | Share of population 65 and older | Brady et al., 2020 | 9,10 | 28,44 | 14,86 | 3,03 | 1200 |
|  | FEMPAR | Share of seats in parliament held by women after the most recent election | Brady et al., 2020 | 1,37 | 47,28 | 21,39 | 12,10 | 858 |
|  | GOVTYPE | Strength of government based on the following classification:  1. technocratic; 2. Single-party minority; 3. Caretaker; 4. Multi-party minority; 5. Minimal winning coalition; 6. Single-party majority; 7. Surplus coalition | Armingeon et al., 2021, own elaboration | 1 | 7 | 4,68 | 1,70 | 1122 |

2. Leave generosity index: data and construction.

For the empirical part of this study, I compile an original leave generosity index, with data extracted from the comparative family policy database (Gauthier, 2011). The method for creating the index is quite straightforward. I use all three existing components in the database: maternal leaves, parental leaves and childcare leaves. For these three different leave types I look at their a) duration and b) replacement rates. Both measures are standardized from 0 to 100. I first calculate the average duration in weeks between the three leaves, and the average replacement rate between the three leaves. The second step is to then calculate the average of these two scores (that is, the average of the averages between leaves’ duration and replacement rates), or as follows:

$${Leave generosity}_{country, year}=\frac{\frac{MLwks+PLwks+CLwks}{3}+\frac{MLpay+PLpay+CLpay}{3}}{2}$$

which is the equivalent of

$${Leave generosity}_{country, year}=\frac{MLwks+PLwks+CLwks+MLpay+PLpay+CLpay}{6}$$

ML = maternal leave

PL = parental leave

CL = childcare leave

wks = standardized weeks (duration)

pay = standardized replacement rates

The index is unweighted, so that all components have the same importance. In the case the country does not provide one of the three schemes (usually this is the case for childcare leaves), or even two are missing, the average is calculated for the existing leave scheme(s). Similarly to Skorge and Rasmussen (2021), the goal of the leave generosity index is to capture both the duration component and its generosity: my basic assumption is that a short but very generous leave has about the same importance as a long but not generous leave.

3. Data interpolation for the variable: female-to-male union density. Red: missing/interpolated values. Blue: actual/existing values. Y axis: value. X axis: time (years). 20 countries. Period: 1980-2010.


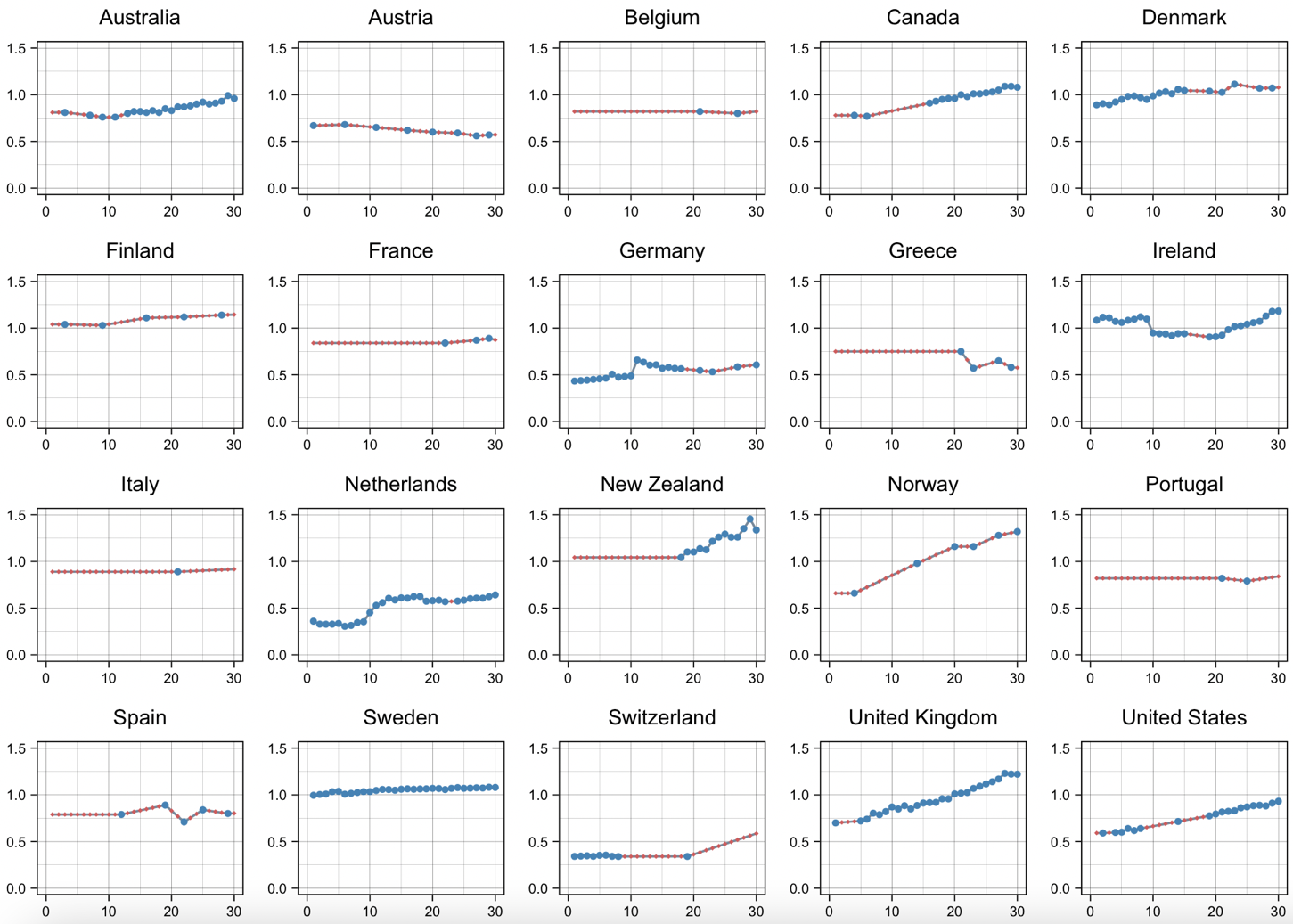


4. Data interpolation for the variable: centralization. Red: missing/interpolated values. Blue: actual/existing values. Y axis: value. X axis: time (years). 20 countries. Period: 1980-2010.


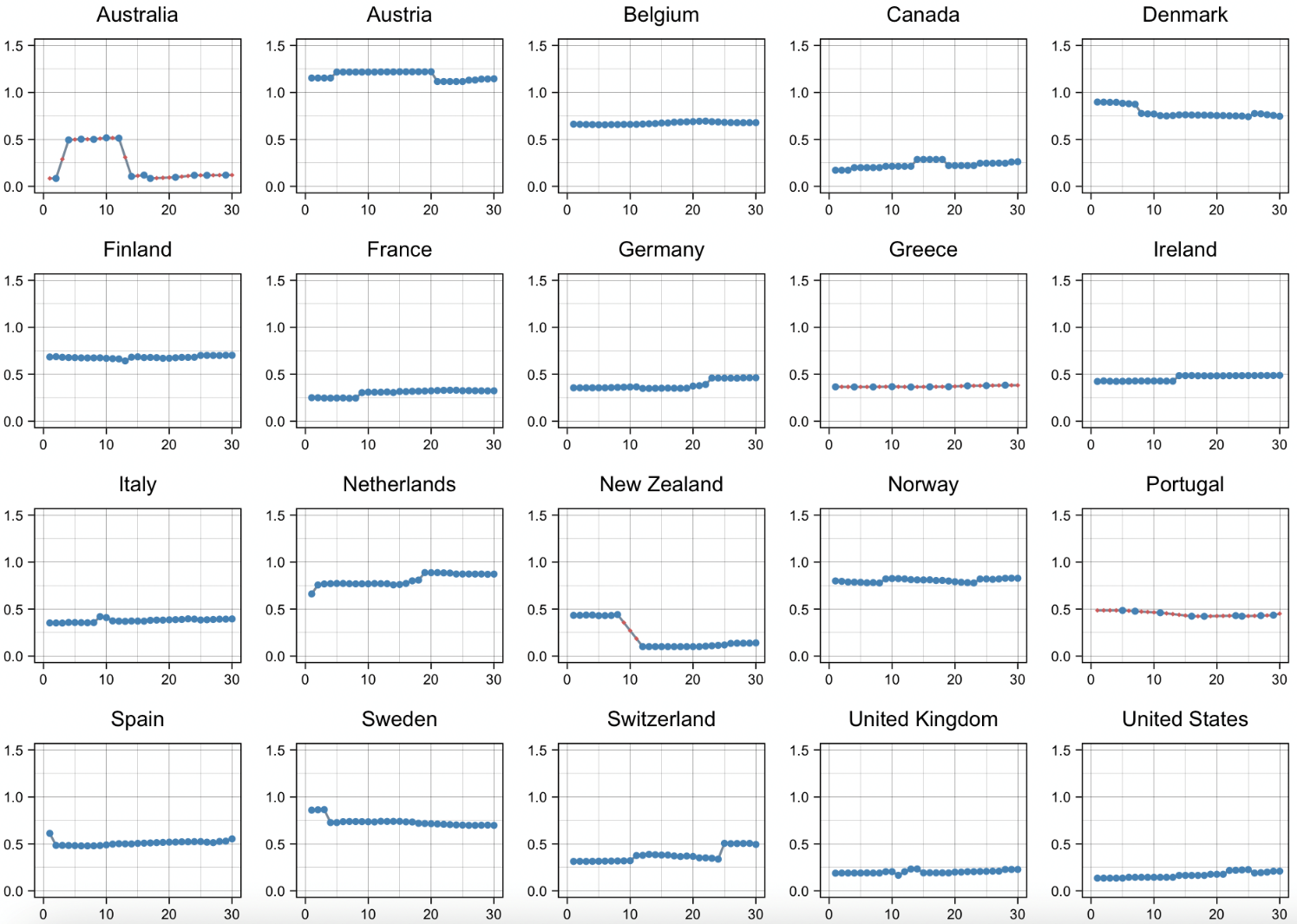


4. Data interpolation for the variable: employers’ density. Red: missing/interpolated values. Blue: actual/existing values. Y axis: value. X axis: time (years). 20 countries. Period: 1980-2010.


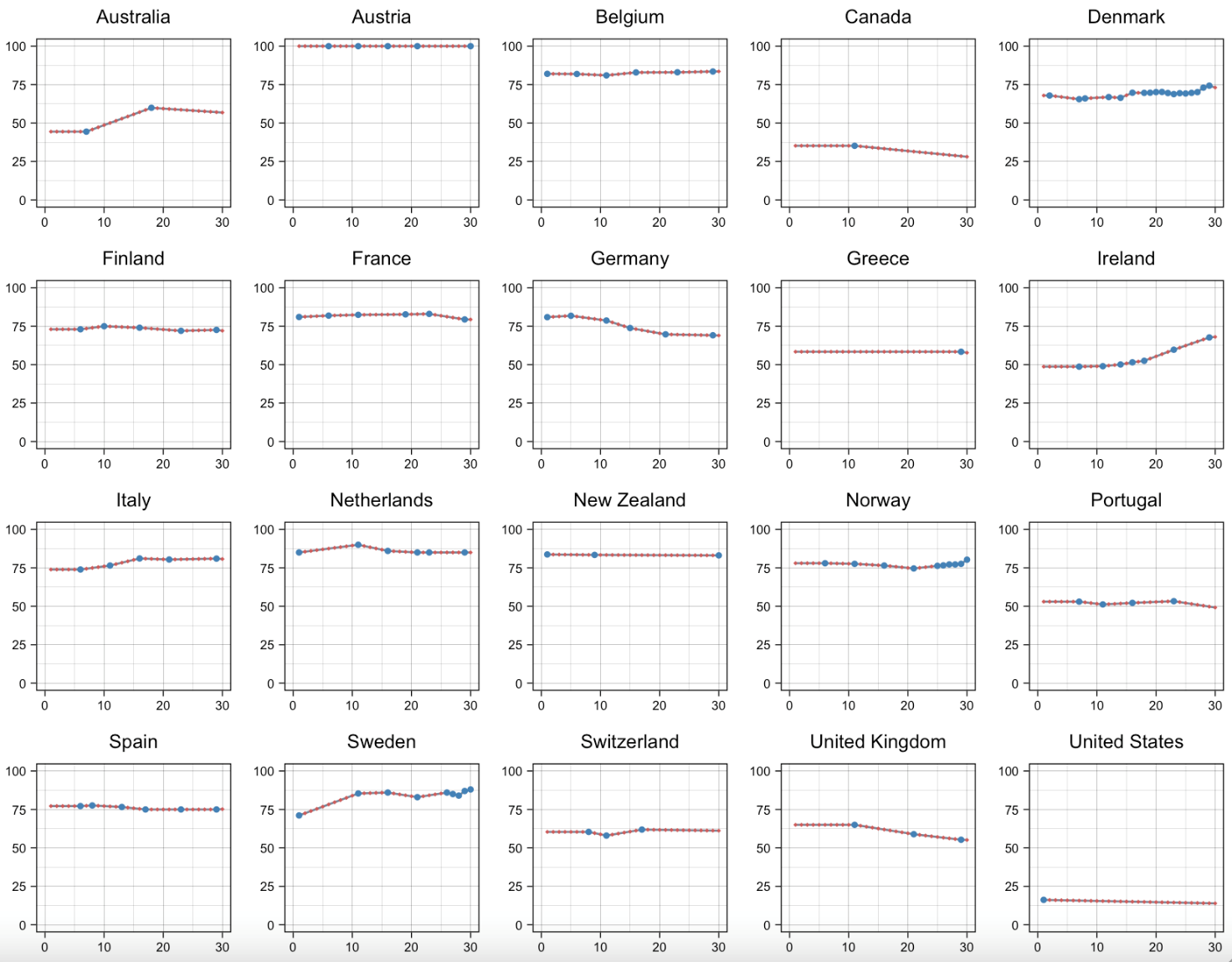


5. Results from fixed-effects designs *without* lagged dependent variables but with interpolated variables (female union density, centralization and employers’ density). Country-robust standard errors in parentheses.

|  | | | | |
| --- | --- | --- | --- | --- |
|  | LVGEN | | MPLEAVE | |
|  | (1) | (2) | (3) | (4) |
|  | | | | |
| FMUD2 | 22.807^**^ | 34.268^***^ | 0.138^**^ | 0.276^***^ |
|  | (10.406) | (12.908) | (0.058) | (0.095) |
|  |  |  |  |  |
| RIV | 0.274 | 1.661 | 0.002 | 0.030 |
|  | (2.408) | (2.275) | (0.047) | (0.050) |
|  |  |  |  |  |
| CENT2 | 2.322 | 2.934 | 0.223 | 0.250 |
|  | (10.243) | (7.516) | (0.467) | (0.400) |
|  |  |  |  |  |
| EMPD2 | 0.025 | 0.151 | 0.001 | 0.005 |
|  | (0.257) | (0.253) | (0.003) | (0.005) |
|  |  |  |  |  |
| FEMP_OECD | 0.563^***^ | 0.585^***^ | 0.0001 | 0.001 |
|  | (0.191) | (0.151) | (0.003) | (0.002) |
|  |  |  |  |  |
| FERRATE | -3.084 | -2.679 | 0.195 | 0.232 |
|  | (2.749) | (2.718) | (0.151) | (0.160) |
|  |  |  |  |  |
| LEFTCAB | -0.003 | -0.007 | 0.00004 | 0.00001 |
|  | (0.011) | (0.011) | (0.0003) | (0.0004) |
|  |  |  |  |  |
| RIGHTCABTOT | -0.002 | -0.003 | 0.0001 | 0.0001 |
|  | (0.008) | (0.009) | (0.0003) | (0.0003) |
|  |  |  |  |  |
| FEMPAR | -0.004 | 0.134 | 0.001 | 0.005 |
|  | (0.069) | (0.110) | (0.002) | (0.004) |
|  |  |  |  |  |
| GOVTYPE_DUMMYweak | 0.835 | 0.348 | 0.010 | 0.007 |
|  | (1.424) | (1.223) | (0.022) | (0.021) |
|  |  |  |  |  |
| ELDPOP |  | 0.925 |  | 0.008 |
|  |  | (0.701) |  | (0.012) |
|  |  |  |  |  |
| UNEMR |  | 0.040 |  | 0.002 |
|  |  | (0.163) |  | (0.004) |
|  |  |  |  |  |
| log(GDPCAP) |  | -7.433 |  | -0.158 |
|  |  | (5.142) |  | (0.098) |
|  |  |  |  |  |
|  | | | | |
| Observations | 414 | 414 | 522 | 522 |
| R^2^ | 0.414 | 0.455 | 0.075 | 0.137 |
| Adjusted R^2^ | 0.370 | 0.409 | 0.022 | 0.082 |
| F Statistic | 27.146^***^ (df = 10; 384) | 24.438^***^ (df = 13; 381) | 3.990^***^ (df = 10; 493) | 5.979^***^ (df = 13; 490) |
|  | | | | |
| Note: | ^**^p<0.1;^**^p<0.05;^***^p<0.01 | | | |

6. Results from fixed-effects designs with lagged dependent variables but *without* interpolated variables. Country-robust standard errors (in parentheses).

|  | | | | |
| --- | --- | --- | --- | --- |
|  | LVGEN | | MPLEAVE | |
|  | (1) | (2) | (3) | (4) |
|  | | | | |
| LAGGED DEP. VAR. | 0.566^***^ | 0.666^***^ | 0.585^***^ | 0.662^***^ |
|  | (0.170) | (0.223) | (0.115) | (0.153) |
|  |  |  |  |  |
|  |  |  |  |  |
| FMUD | -18.215^**^ | -28.963^**^ | 0.043 | 0.231^**^ |
|  | (8.033) | (13.743) | (0.049) | (0.108) |
|  |  |  |  |  |
| RIV | -0.738 | -3.654 | -0.038 | -0.194 |
|  | (1.623) | (4.546) | (0.036) | (0.128) |
|  |  |  |  |  |
| CENT | -36.321^***^ | -41.173^***^ | 0.181 | -1.246^*^ |
|  | (6.966) | (15.035) | (0.789) | (0.701) |
|  |  |  |  |  |
| EMPDOECD | 0.242^**^ | -0.042 | 0.003 | 0.002 |
|  | (0.109) | (0.203) | (0.003) | (0.004) |
|  |  |  |  |  |
| FEMP_OECD | 0.144^**^ | 0.200^*^ | 0.001 | 0.003 |
|  | (0.065) | (0.108) | (0.002) | (0.002) |
|  |  |  |  |  |
| FERRATE | 3.056 | 8.218^*^ | 0.119 | -0.056 |
|  | (3.755) | (4.295) | (0.288) | (0.308) |
|  |  |  |  |  |
| LEFTCAB | -0.020 | 0.030 | 0.0002 | 0.001^**^ |
|  | (0.037) | (0.042) | (0.001) | (0.001) |
|  |  |  |  |  |
| RIGHTCABTOT | -0.086^***^ | -0.034 | -0.0002 | 0.001 |
|  | (0.032) | (0.044) | (0.002) | (0.001) |
|  |  |  |  |  |
| FEMPAR | 0.186 | -0.289 | -0.001 | -0.003 |
|  | (0.139) | (0.282) | (0.003) | (0.004) |
|  |  |  |  |  |
| GOVTYPE_DUMMYweak | -3.342 | -3.392^**^ | -0.010 | -0.092^**^ |
|  | (2.047) | (1.421) | (0.013) | (0.036) |
|  |  |  |  |  |
| ELDPOP |  | -2.071 |  | 0.071^**^ |
|  |  | (1.953) |  | (0.030) |
|  |  |  |  |  |
| UNEMR |  | 0.180 |  | -0.025^**^ |
|  |  | (0.281) |  | (0.011) |
|  |  |  |  |  |
| log(GDPCAP) |  | 10.255 |  | -0.046 |
|  |  | (8.844) |  | (0.044) |
|  |  |  |  |  |
|  | | | | |
| Observations | 38 | 38 | 45 | 45 |
| R^2^ | 0.896 | 0.912 | 0.406 | 0.543 |
| Adjusted R^2^ | 0.724 | 0.705 | -0.245 | -0.116 |
| F Statistic | 10.925^***^ (df = 11; 14) | 8.176^***^ (df = 14; 11) | 1.304 (df = 11; 21) | 1.530 (df = 14; 18) |
|  | | | | |
| Note: | ^**^p<0.1;^**^p<0.05;^***^p<0.01 | | | |

7. Results from fixed-effects designs using alternative leave generosity measures: only maternal leaves, only parental leaves, and both maternal and parental (but not childcare) leaves. Alternative models with only childcare leaves are excluded for low number of cases. Country-robust standard errors (in parentheses).

|  | | | | | | |
| --- | --- | --- | --- | --- | --- | --- |
|  | MAT. LEAVES | | PARENT. LEAVES | | MAT+PARENT. LEAVES | |
|  | (1) | (2) | (3) | (4) | (5) | (6) |
|  | | | | | | |
| LAGGED DEP. VAR. | 0.819^***^ | 0.804^***^ | 0.736^***^ | 0.726^***^ | 0.686^***^ | 0.686^***^ |
|  | (0.045) | (0.041) | (0.037) | (0.032) | (0.041) | (0.041) |
|  |  |  |  |  |  |  |
|  |  |  |  |  |  |  |
| FMUD2 | 3.411 | 4.544 | 11.495^**^ | 16.954^**^ | 13.272^***^ | 13.272^***^ |
|  | (3.079) | (3.825) | (5.216) | (7.910) | (4.587) | (4.587) |
|  |  |  |  |  |  |  |
| RIV | -0.545 | -0.172 | 0.792 | 0.635 | 0.331 | 0.331 |
|  | (0.888) | (0.936) | (2.057) | (2.069) | (1.042) | (1.042) |
|  |  |  |  |  |  |  |
| CENT2 | 11.160 | 10.600 | -8.973^**^ | -8.986^*^ | 2.051 | 2.051 |
|  | (10.133) | (9.612) | (4.564) | (4.639) | (4.554) | (4.554) |
|  |  |  |  |  |  |  |
| EMPD2 | 0.070 | 0.108 | 0.282^**^ | 0.316^**^ | 0.237^***^ | 0.237^***^ |
|  | (0.081) | (0.090) | (0.137) | (0.131) | (0.088) | (0.088) |
|  |  |  |  |  |  |  |
| FEMP_OECD | 0.097 | 0.108 | 0.016 | -0.001 | 0.085 | 0.085 |
|  | (0.094) | (0.097) | (0.057) | (0.055) | (0.060) | (0.060) |
|  |  |  |  |  |  |  |
| FERRATE | -1.444 | -0.953 | -0.969 | -1.958 | -1.662 | -1.662 |
|  | (1.881) | (1.952) | (3.496) | (3.455) | (1.572) | (1.572) |
|  |  |  |  |  |  |  |
| LEFTCAB | -0.005 | -0.007 | 0.004 | 0.003 | -0.003 | -0.003 |
|  | (0.005) | (0.005) | (0.010) | (0.011) | (0.006) | (0.006) |
|  |  |  |  |  |  |  |
| RIGHTCABTOT | -0.002 | -0.003 | 0.002 | 0.0004 | -0.0004 | -0.0004 |
|  | (0.004) | (0.004) | (0.009) | (0.011) | (0.006) | (0.006) |
|  |  |  |  |  |  |  |
| FEMPAR | -0.046 | -0.045 | 0.084^**^ | 0.088 | 0.030 | 0.030 |
|  | (0.065) | (0.075) | (0.040) | (0.060) | (0.034) | (0.034) |
|  |  |  |  |  |  |  |
| GOVTYPE_DUMMYweak | 0.056 | 0.088 | 2.004^*^ | 1.767^*^ | 0.929^*^ | 0.929^*^ |
|  | (0.294) | (0.303) | (1.167) | (1.033) | (0.524) | (0.524) |
|  |  |  |  |  |  |  |
| ELDPOP |  | 0.213 |  | 0.658^**^ | 0.574^***^ | 0.574^***^ |
|  |  | (0.191) |  | (0.326) | (0.209) | (0.209) |
|  |  |  |  |  |  |  |
| UNEMR |  | 0.187^**^ |  | -0.260^**^ | -0.041 | -0.041 |
|  |  | (0.082) |  | (0.107) | (0.062) | (0.062) |
|  |  |  |  |  |  |  |
| log(GDPCAP) |  | -0.122 |  | -3.160 | -2.374^*^ | -2.374^*^ |
|  |  | (1.279) |  | (2.040) | (1.256) | (1.256) |
|  |  |  |  |  |  |  |
|  | | | | | | |
| Observations | 414 | 414 | 414 | 414 | 414 | 414 |
| R^2^ | 0.760 | 0.765 | 0.769 | 0.773 | 0.753 | 0.753 |
| Adjusted R^2^ | 0.741 | 0.745 | 0.751 | 0.753 | 0.731 | 0.731 |
| F Statistic | 110.339^***^ (df = 11; 383) | 88.345^***^ (df = 14; 380) | 115.897^***^ (df = 11; 383) | 92.360^***^ (df = 14; 380) | 82.582^***^ (df = 14; 380) | 82.582^***^ (df = 14; 380) |
|  | | | | | | |
| Note: | ^**^p<0.1;^**^p<0.05;^***^p<0.01 | | | | | |

8. Descriptive statistics: main variables (30 countries, 1980-2019).

| **Country** | **Year** | **Leave generosity** | **Leave spending** | **F/M union density** | **Interpolated F/M union density** | **Centralization** | **Interpolated centralization** | **Routine inv. in policy-making** |
| --- | --- | --- | --- | --- | --- | --- | --- | --- |
| Australia | 1980 | 0 |  |  | 0,81 |  | 0,08485281 | 0,00 |
| Australia | 1981 | 0 |  |  | 0,81 | 0,08 | 0,08485281 | 0,00 |
| Australia | 1982 | 0 |  | 0,81 | 0,81 |  | 0,28960686 | 0,00 |
| Australia | 1983 | 0 |  |  | 0,8025 | 0,49 | 0,49436091 | 0,00 |
| Australia | 1984 | 0 |  |  | 0,795 |  | 0,49865539 | 0,50 |
| Australia | 1985 | 0 |  |  | 0,7875 | 0,50 | 0,50294986 | 0,50 |
| Australia | 1986 | 0 |  | 0,78 | 0,78 |  | 0,50173976 | 0,50 |
| Australia | 1987 | 0 |  |  | 0,77 | 0,50 | 0,50052965 | 0,50 |
| Australia | 1988 | 0 |  | 0,76 | 0,76 |  | 0,50878378 | 0,50 |
| Australia | 1989 | 0 |  |  | 0,76 | 0,52 | 0,5170379 | 0,50 |
| Australia | 1990 | 0 |  | 0,76 | 0,76 |  | 0,51504252 | 0,50 |
| Australia | 1991 | 0 |  |  | 0,78 | 0,51 | 0,51304713 | 0,50 |
| Australia | 1992 | 0 |  | 0,80 | 0,8 |  | 0,30943859 | 0,50 |
| Australia | 1993 | 0 |  | 0,82 | 0,82 | 0,11 | 0,10583005 | 0,50 |
| Australia | 1994 | 0 |  | 0,82 | 0,82 |  | 0,11291503 | 0,50 |
| Australia | 1995 | 0 | 0,01 | 0,81 | 0,81 | 0,12 | 0,12 | 0,50 |
| Australia | 1996 | 0 | 0,04 | 0,83 | 0,83 | 0,08 | 0,08485281 | 0,00 |
| Australia | 1997 | 9,2395167 | 0,03 | 0,81 | 0,81 |  | 0,08787801 | 0,00 |
| Australia | 1998 | 9,2395167 | 0,03 | 0,85 | 0,85 |  | 0,09090321 | 0,00 |
| Australia | 1999 | 9,2395167 | 0,03 | 0,83 | 0,83 |  | 0,0939284 | 0,00 |
| Australia | 2000 | 9,2395167 | 0,03 | 0,87 | 0,87 | 0,10 | 0,0969536 | 0,00 |
| Australia | 2001 | 9,2395167 | 0,03 | 0,87 | 0,87 |  | 0,10407626 | 0,00 |
| Australia | 2002 | 9,2395167 | 0,03 | 0,88 | 0,88 |  | 0,11119893 | 0,00 |
| Australia | 2003 | 9,2395167 | 0,03 | 0,90 | 0,9 | 0,12 | 0,1183216 | 0,00 |
| Australia | 2004 | 9,2395167 | 0,09 | 0,92 | 0,92 |  | 0,1181608 | 0,00 |
| Australia | 2005 | 9,2395167 | 0,09 | 0,90 | 0,9 | 0,12 | 0,118 | 0,00 |
| Australia | 2006 | 9,2395167 | 0,11 | 0,91 | 0,91 |  | 0,11866667 | 0,00 |
| Australia | 2007 | 9,2395167 | 0,11 | 0,93 | 0,93 |  | 0,11933333 | 0,50 |
| Australia | 2008 | 9,2395167 | 0,12 | 0,99 | 0,99 | 0,12 | 0,12 | 0,50 |
| Australia | 2009 | 9,2395167 | 0,11 | 0,96 | 0,96 |  | 0,1204949 | 0,50 |
| Australia | 2010 | 9,2395167 | 0,13 | 1,04 | 1,04 |  | 0,12098979 | 0,50 |
| Australia | 2011 |  | 0,16 | 0,99 | 0,99 |  | 0,12148469 | 0,50 |
| Australia | 2012 |  | 0,14 | 1,08 | 1,08 |  | 0,12197959 | 0,50 |
| Australia | 2013 |  | 0,13 | 1,09 | 1,09 | 0,12 | 0,12247449 | 0,50 |
| Australia | 2014 |  | 0,12 | 1,10 | 1,1 |  | 0,12247449 | 0,50 |
| Australia | 2015 |  | 0,13 |  | 1,15 | 0,12 | 0,12247449 | 0,50 |
| Australia | 2016 |  | 0,12 | 1,20 | 1,2 |  | 0,12288138 | 0,50 |
| Australia | 2017 |  | 0,12 |  | 1,2 | 0,12 | 0,12328828 | 0,50 |
| Australia | 2018 |  |  |  | 1,2 |  | 0,12328828 | 0,50 |
| Australia | 2019 |  |  |  | 1,2 |  | 0,12328828 |  |
| Austria | 1980 | 41,5030137 | 0,42 | 0,67 | 0,67 | 1,15 | 1,15362291 | 1,00 |
| Austria | 1981 | 41,5030137 |  |  | 0,672 | 1,15 | 1,15362291 | 1,00 |
| Austria | 1982 | 41,5030137 |  |  | 0,674 | 1,15 | 1,15362291 | 1,00 |
| Austria | 1983 | 41,5030137 |  |  | 0,676 | 1,15 | 1,15362291 | 1,00 |
| Austria | 1984 | 41,5030137 |  |  | 0,678 | 1,22 | 1,21725561 | 1,00 |
| Austria | 1985 | 41,5030137 | 0,38 | 0,68 | 0,68 | 1,22 | 1,21725561 | 1,00 |
| Austria | 1986 | 41,5030137 |  |  | 0,674 | 1,22 | 1,21725561 | 1,00 |
| Austria | 1987 | 41,5030137 |  |  | 0,668 | 1,22 | 1,21725561 | 1,00 |
| Austria | 1988 | 41,5030137 |  |  | 0,662 | 1,22 | 1,21725561 | 1,00 |
| Austria | 1989 | 41,5030137 |  |  | 0,656 | 1,22 | 1,21725561 | 1,00 |
| Austria | 1990 | 50,7425304 | 0,39 | 0,65 | 0,65 | 1,22 | 1,21725561 | 1,00 |
| Austria | 1991 | 50,7425304 | 0,44 |  | 0,644 | 1,22 | 1,21908902 | 1,00 |
| Austria | 1992 | 50,7425304 | 0,62 |  | 0,638 | 1,22 | 1,21908902 | 1,00 |
| Austria | 1993 | 50,7425304 | 0,68 |  | 0,632 | 1,22 | 1,21908902 | 1,00 |
| Austria | 1994 | 50,1866185 | 0,67 |  | 0,626 | 1,22 | 1,22 | 1,00 |
| Austria | 1995 | 50,1866185 | 0,63 | 0,62 | 0,62 | 1,22 | 1,22 | 1,00 |
| Austria | 1996 | 48,4922451 | 0,61 |  | 0,615 | 1,22 | 1,22 | 1,00 |
| Austria | 1997 | 48,4922451 | 0,51 |  | 0,61 | 1,22 | 1,22 | 1,00 |
| Austria | 1998 | 48,4922451 | 0,41 |  | 0,605 | 1,22 | 1,22 | 1,00 |
| Austria | 1999 | 48,4922451 | 0,36 | 0,60 | 0,6 | 1,22 | 1,22090722 | 1,00 |
| Austria | 2000 | 48,4922451 | 0,35 |  | 0,5975 | 1,12 | 1,11623792 | 0,50 |
| Austria | 2001 | 48,4922451 | 0,36 |  | 0,595 | 1,12 | 1,11623792 | 0,50 |
| Austria | 2002 | 47,013427 | 0,38 |  | 0,5925 | 1,12 | 1,11623792 | 0,50 |
| Austria | 2003 | 46,9585485 | 0,29 | 0,59 | 0,59 | 1,12 | 1,11623792 | 0,50 |
| Austria | 2004 | 46,90367 | 0,17 |  | 0,58 | 1,12 | 1,11623792 | 0,50 |
| Austria | 2005 | 46,8487916 | 0,14 |  | 0,57 | 1,13 | 1,13022371 | 0,50 |
| Austria | 2006 | 46,7939131 | 0,14 | 0,56 | 0,56 | 1,13 | 1,13191403 | 1,00 |
| Austria | 2007 | 46,7288751 | 0,13 |  | 0,565 | 1,14 | 1,14262066 | 1,00 |
| Austria | 2008 | 46,6567119 | 0,13 | 0,57 | 0,57 | 1,14 | 1,14342518 | 1,00 |
| Austria | 2009 | 46,5870903 | 0,14 |  | 0,57142857 | 1,15 | 1,14582329 | 1,00 |
| Austria | 2010 | 46,5198784 | 0,15 |  | 0,57285714 | 1,15 | 1,15210917 | 1,00 |
| Austria | 2011 |  | 0,13 |  | 0,57428571 | 1,15 | 1,15210917 | 1,00 |
| Austria | 2012 |  | 0,13 |  | 0,57571429 | 1,15 | 1,15210917 | 1,00 |
| Austria | 2013 |  | 0,14 |  | 0,57714286 | 1,15 | 1,15288415 | 1,00 |
| Austria | 2014 |  | 0,14 |  | 0,57857143 | 1,15 | 1,15365682 | 1,00 |
| Austria | 2015 |  | 0,14 | 0,58 | 0,58 | 1,16 | 1,15519529 | 1,00 |
| Austria | 2016 |  | 0,14 |  | 0,58 | 1,16 | 1,15596113 | 1,00 |
| Austria | 2017 |  | 0,14 |  | 0,58 | 1,16 | 1,15596113 | 1,00 |
| Austria | 2018 |  |  |  | 0,58 | 1,16 | 1,15596113 | 1,00 |
| Austria | 2019 |  |  |  | 0,58 |  | 1,15596113 |  |
| Belgium | 1980 | 19,4856379 | 0,08 |  | 0,82 | 0,66 | 0,66238914 | 1,00 |
| Belgium | 1981 | 19,4856379 | 0,08 |  | 0,82 | 0,66 | 0,66076108 | 1,00 |
| Belgium | 1982 | 19,4856379 | 0,08 |  | 0,82 | 0,66 | 0,65930761 | 1,00 |
| Belgium | 1983 | 19,4856379 | 0,08 |  | 0,82 | 0,66 | 0,65849063 | 1,00 |
| Belgium | 1984 | 19,4856379 | 0,08 |  | 0,82 | 0,66 | 0,65662527 | 1,00 |
| Belgium | 1985 | 33,5646848 | 0,08 |  | 0,82 | 0,66 | 0,65639729 | 1,00 |
| Belgium | 1986 | 33,5646848 | 0,08 |  | 0,82 | 0,66 | 0,65842957 | 1,00 |
| Belgium | 1987 | 32,9018667 | 0,07 |  | 0,82 | 0,66 | 0,65867651 | 1,00 |
| Belgium | 1988 | 32,9018667 | 0,07 |  | 0,82 | 0,66 | 0,66017144 | 1,00 |
| Belgium | 1989 | 32,9018667 | 0,07 |  | 0,82 | 0,66 | 0,66027221 | 1,00 |
| Belgium | 1990 | 33,0795498 | 0,15 |  | 0,82 | 0,66 | 0,66056742 | 1,00 |
| Belgium | 1991 | 33,0795498 | 0,17 |  | 0,82 | 0,66 | 0,66385156 | 1,00 |
| Belgium | 1992 | 33,0795498 | 0,17 |  | 0,82 | 0,67 | 0,66672711 | 1,00 |
| Belgium | 1993 | 33,2078371 | 0,17 |  | 0,82 | 0,67 | 0,66863704 | 1,00 |
| Belgium | 1994 | 33,2078371 | 0,16 |  | 0,82 | 0,67 | 0,67432135 | 1,00 |
| Belgium | 1995 | 33,2078371 | 0,15 |  | 0,82 | 0,68 | 0,67503001 | 1,00 |
| Belgium | 1996 | 33,2078371 | 0,16 |  | 0,82 | 0,68 | 0,68313305 | 1,00 |
| Belgium | 1997 | 33,2078371 | 0,17 |  | 0,82 | 0,68 | 0,68482748 | 1,00 |
| Belgium | 1998 | 42,8089995 | 0,16 |  | 0,82 | 0,69 | 0,687353 | 1,00 |
| Belgium | 1999 | 42,8565068 | 0,16 |  | 0,82 | 0,69 | 0,68925448 | 1,00 |
| Belgium | 2000 | 31,3984435 | 0,17 | 0,82 | 0,82 | 0,69 | 0,69278891 | 1,00 |
| Belgium | 2001 | 31,4697045 | 0,18 |  | 0,81666667 | 0,69 | 0,69460571 | 1,00 |
| Belgium | 2002 | 31,5409654 | 0,18 |  | 0,81333333 | 0,69 | 0,68859659 | 1,00 |
| Belgium | 2003 | 31,6122263 | 0,19 |  | 0,81 | 0,69 | 0,68581254 | 1,00 |
| Belgium | 2004 | 31,6834873 | 0,19 |  | 0,80666667 | 0,68 | 0,68152216 | 1,00 |
| Belgium | 2005 | 31,4489855 | 0,19 |  | 0,80333333 | 0,68 | 0,67893127 | 1,00 |
| Belgium | 2006 | 32,6889493 | 0,20 | 0,80 | 0,8 | 0,68 | 0,67806903 | 1,00 |
| Belgium | 2007 | 32,6516818 | 0,20 |  | 0,80666667 | 0,68 | 0,67770707 | 1,00 |
| Belgium | 2008 | 33,2667961 | 0,21 |  | 0,81333333 | 0,68 | 0,67854619 | 1,00 |
| Belgium | 2009 | 31,9788713 | 0,22 |  | 0,82 | 0,68 | 0,678539 | 1,00 |
| Belgium | 2010 | 31,9339364 | 0,23 |  | 0,82666667 | 0,68 | 0,67887105 | 1,00 |
| Belgium | 2011 |  | 0,24 |  | 0,83333333 | 0,68 | 0,67839358 | 1,00 |
| Belgium | 2012 |  | 0,24 | 0,84 | 0,84 | 0,68 | 0,67834478 | 1,00 |
| Belgium | 2013 |  | 0,24 |  | 0,84 | 0,68 | 0,67799405 | 1,00 |
| Belgium | 2014 |  | 0,23 |  | 0,84 | 0,68 | 0,68184329 | 1,00 |
| Belgium | 2015 |  | 0,23 |  | 0,84 | 0,68 | 0,6821048 | 1,00 |
| Belgium | 2016 |  | 0,22 |  | 0,84 | 0,68 | 0,68259489 | 1,00 |
| Belgium | 2017 |  | 0,23 |  | 0,84 | 0,68 | 0,6831889 | 1,00 |
| Belgium | 2018 |  |  |  | 0,84 | 0,68 | 0,68303026 | 1,00 |
| Belgium | 2019 |  |  |  | 0,84 |  | 0,68303026 |  |
| Canada | 1980 | 15,4939816 | 0,07 |  | 0,78 | 0,17 | 0,17204651 | 0,00 |
| Canada | 1981 | 15,4939816 | 0,08 |  | 0,78 | 0,17 | 0,17204651 | 0,00 |
| Canada | 1982 | 15,4939816 | 0,08 |  | 0,78 | 0,17 | 0,17204651 | 0,00 |
| Canada | 1983 | 15,4939816 | 0,08 | 0,78 | 0,78 | 0,20 | 0,2 | 0,00 |
| Canada | 1984 | 15,4939816 | 0,09 |  | 0,775 | 0,20 | 0,2 | 0,00 |
| Canada | 1985 | 15,4939816 | 0,09 | 0,77 | 0,77 | 0,20 | 0,2 | 0,00 |
| Canada | 1986 | 15,4939816 | 0,09 |  | 0,784 | 0,20 | 0,2 | 0,00 |
| Canada | 1987 | 15,4939816 | 0,09 |  | 0,798 | 0,20 | 0,2 | 0,00 |
| Canada | 1988 | 15,4939816 | 0,09 |  | 0,812 | 0,21 | 0,21354157 | 0,00 |
| Canada | 1989 | 15,4939816 | 0,10 |  | 0,826 | 0,21 | 0,21354157 | 0,00 |
| Canada | 1990 | 30,0995481 | 0,11 |  | 0,84 | 0,21 | 0,21354157 | 0,00 |
| Canada | 1991 | 30,0995481 | 0,18 |  | 0,854 | 0,21 | 0,21354157 | 0,00 |
| Canada | 1992 | 30,0995481 | 0,18 |  | 0,868 | 0,21 | 0,21354157 | 0,00 |
| Canada | 1993 | 29,6719235 | 0,17 |  | 0,882 | 0,29 | 0,287054 | 0,00 |
| Canada | 1994 | 29,6719235 | 0,16 |  | 0,896 | 0,29 | 0,287054 | 0,00 |
| Canada | 1995 | 27,9614253 | 0,15 | 0,91 | 0,91 | 0,29 | 0,287054 | 0,00 |
| Canada | 1996 | 27,9614253 | 0,14 | 0,93 | 0,93 | 0,29 | 0,287054 | 0,00 |
| Canada | 1997 | 27,9614253 | 0,13 | 0,95 | 0,95 | 0,29 | 0,287054 | 0,00 |
| Canada | 1998 | 27,9614253 | 0,12 | 0,96 | 0,96 | 0,22 | 0,22090722 | 0,00 |
| Canada | 1999 | 27,9614253 | 0,12 | 0,96 | 0,96 | 0,22 | 0,22090722 | 0,00 |
| Canada | 2000 | 27,9614253 | 0,11 | 1,00 | 1 | 0,22 | 0,22090722 | 0,00 |
| Canada | 2001 | 32,4035007 | 0,18 | 0,98 | 0,98 | 0,22 | 0,22090722 | 0,00 |
| Canada | 2002 | 32,4035007 | 0,23 | 1,01 | 1,01 | 0,22 | 0,22090722 | 0,00 |
| Canada | 2003 | 32,4035007 | 0,23 | 1,01 | 1,01 | 0,25 | 0,24657656 | 0,00 |
| Canada | 2004 | 32,4035007 | 0,22 | 1,02 | 1,02 | 0,25 | 0,24657656 | 0,00 |
| Canada | 2005 | 32,4035007 | 0,22 | 1,03 | 1,03 | 0,25 | 0,24657656 | 0,00 |
| Canada | 2006 | 32,4035007 | 0,24 | 1,05 | 1,05 | 0,25 | 0,24819347 | 0,00 |
| Canada | 2007 | 32,4035007 | 0,27 | 1,09 | 1,09 | 0,25 | 0,24657656 | 0,00 |
| Canada | 2008 | 32,4035007 | 0,28 | 1,09 | 1,09 | 0,26 | 0,25922963 | 0,00 |
| Canada | 2009 | 32,4035007 | 0,30 | 1,08 | 1,08 | 0,26 | 0,26229754 | 0,00 |
| Canada | 2010 | 32,4035007 | 0,28 | 1,09 | 1,09 | 0,27 | 0,26683328 | 0,00 |
| Canada | 2011 |  | 0,27 | 1,10 | 1,1 | 0,27 | 0,26683328 | 0,00 |
| Canada | 2012 |  | 0,28 | 1,12 | 1,12 | 0,27 | 0,26683328 | 0,00 |
| Canada | 2013 |  | 0,28 | 1,13 | 1,13 | 0,27 | 0,26532998 | 0,00 |
| Canada | 2014 |  | 0,28 | 1,15 | 1,15 | 0,26 | 0,26381812 | 0,00 |
| Canada | 2015 |  | 0,28 | 1,13 | 1,13 | 0,26 | 0,2607681 | 0,00 |
| Canada | 2016 |  | 0,28 |  | 1,13 |  | 0,2607681 | 0,00 |
| Canada | 2017 |  | 0,27 |  | 1,13 |  | 0,2607681 | 0,00 |
| Canada | 2018 |  | 0,26 |  | 1,13 |  | 0,2607681 | 0,00 |
| Canada | 2019 |  |  |  | 1,13 |  | 0,2607681 |  |
| Czech Republic | 1980 |  |  |  | 1,01 |  | 0,97459667 |  |
| Czech Republic | 1981 |  |  |  | 1,01 |  | 0,97459667 |  |
| Czech Republic | 1982 |  |  |  | 1,01 |  | 0,97459667 |  |
| Czech Republic | 1983 |  |  |  | 1,01 |  | 0,97459667 |  |
| Czech Republic | 1984 |  |  |  | 1,01 |  | 0,97459667 |  |
| Czech Republic | 1985 |  |  |  | 1,01 |  | 0,97459667 |  |
| Czech Republic | 1986 |  |  |  | 1,01 |  | 0,97459667 |  |
| Czech Republic | 1987 |  |  |  | 1,01 |  | 0,97459667 |  |
| Czech Republic | 1988 |  |  |  | 1,01 |  | 0,97459667 |  |
| Czech Republic | 1989 |  |  |  | 1,01 |  | 0,97459667 |  |
| Czech Republic | 1990 |  | 0,43 |  | 1,01 | 0,97 | 0,97459667 | 1,00 |
| Czech Republic | 1991 |  | 0,54 |  | 1,01 | 0,97 | 0,97459667 | 1,00 |
| Czech Republic | 1992 |  | 0,57 |  | 1,01 | 0,97 | 0,97459667 | 0,50 |
| Czech Republic | 1993 |  | 0,55 |  | 1,01 | 0,97 | 0,97459667 | 0,50 |
| Czech Republic | 1994 |  | 0,53 |  | 1,01 | 0,97 | 0,97459667 | 0,50 |
| Czech Republic | 1995 |  | 0,52 |  | 1,01 | 0,62 | 0,61964006 | 0,00 |
| Czech Republic | 1996 |  | 0,53 |  | 1,01 | 0,62 | 0,61691092 | 0,00 |
| Czech Republic | 1997 |  | 0,51 |  | 1,01 | 0,60 | 0,59808064 | 0,00 |
| Czech Republic | 1998 |  | 0,48 |  | 1,01 | 0,59 | 0,59479354 | 0,50 |
| Czech Republic | 1999 |  | 0,46 |  | 1,01 | 0,59 | 0,58957797 | 0,50 |
| Czech Republic | 2000 |  | 0,46 | 1,01 | 1,01 | 0,59 | 0,59298639 | 0,50 |
| Czech Republic | 2001 |  | 0,44 |  | 1,01 | 0,59 | 0,58894549 | 0,50 |
| Czech Republic | 2002 |  | 0,46 |  | 1,01 | 0,59 | 0,58587506 | 0,50 |
| Czech Republic | 2003 |  | 0,44 |  | 1,01 | 0,57 | 0,56645879 | 0,50 |
| Czech Republic | 2004 |  | 0,50 |  | 1,01 | 0,55 | 0,5526207 | 0,50 |
| Czech Republic | 2005 |  | 0,55 |  | 1,01 | 0,55 | 0,55071461 | 0,50 |
| Czech Republic | 2006 |  | 0,57 | 1,01 | 1,01 | 0,55 | 0,54941614 | 0,50 |
| Czech Republic | 2007 |  | 0,95 |  | 1,02833333 | 0,55 | 0,55044595 | 0,50 |
| Czech Republic | 2008 |  | 0,90 |  | 1,04666667 | 0,55 | 0,55280744 | 0,50 |
| Czech Republic | 2009 |  | 0,94 |  | 1,065 | 0,55 | 0,55209652 | 0,50 |
| Czech Republic | 2010 |  | 0,92 |  | 1,08333333 | 0,55 | 0,54966891 | 0,50 |
| Czech Republic | 2011 |  | 0,83 |  | 1,10166667 | 0,55 | 0,54608671 | 0,50 |
| Czech Republic | 2012 |  | 0,79 | 1,12 | 1,12 | 0,55 | 0,54717221 | 0,50 |
| Czech Republic | 2013 |  | 0,77 |  | 1,006 | 0,55 | 0,55476134 | 0,50 |
| Czech Republic | 2014 |  | 0,70 |  | 0,892 | 0,55 | 0,55345637 | 1,00 |
| Czech Republic | 2015 |  | 0,66 |  | 0,778 | 0,49 | 0,49478201 | 1,00 |
| Czech Republic | 2016 |  | 0,65 |  | 0,664 | 0,50 | 0,49727966 | 1,00 |
| Czech Republic | 2017 |  | 0,63 | 0,55 | 0,55 | 0,50 | 0,49685965 | 1,00 |
| Czech Republic | 2018 |  |  |  | 0,55 |  | 0,49685965 | 1,00 |
| Czech Republic | 2019 |  |  |  | 0,55 |  | 0,49685965 |  |
| Denmark | 1980 | 22,4413988 | 0,24 | 0,89 | 0,89084507 | 0,90 | 0,8974939 | 1,00 |
| Denmark | 1981 | 22,4413988 | 0,29 | 0,90 | 0,90420561 | 0,90 | 0,89628723 | 1,00 |
| Denmark | 1982 | 22,4413988 | 0,28 | 0,89 | 0,89192264 | 0,89 | 0,89440961 | 1,00 |
| Denmark | 1983 | 22,4413988 | 0,28 | 0,92 | 0,92138728 | 0,89 | 0,89472668 | 1,00 |
| Denmark | 1984 | 22,4413988 | 0,31 | 0,95 | 0,94964871 | 0,89 | 0,88501855 | 1,00 |
| Denmark | 1985 | 43,4613335 | 0,36 | 0,98 | 0,98170732 | 0,88 | 0,88014675 | 1,00 |
| Denmark | 1986 | 43,4613335 | 0,39 | 0,99 | 0,98628429 | 0,87 | 0,87449802 | 1,00 |
| Denmark | 1987 | 43,4613335 | 0,41 | 0,97 | 0,96940024 | 0,78 | 0,77666887 | 1,00 |
| Denmark | 1988 | 43,4613335 | 0,43 | 0,95 | 0,94909091 | 0,77 | 0,77219701 | 1,00 |
| Denmark | 1989 | 43,4613335 | 0,44 | 0,99 | 0,98655257 | 0,77 | 0,7710132 | 1,00 |
| Denmark | 1990 | 43,4613335 | 0,46 | 1,02 | 1,01713586 | 0,75 | 0,75320352 | 1,00 |
| Denmark | 1991 | 34,6519427 | 0,46 | 1,03 | 1,03292683 | 0,75 | 0,74970454 | 1,00 |
| Denmark | 1992 | 54,1828962 | 0,48 | 1,01 | 1,01043025 | 0,75 | 0,75351972 | 1,00 |
| Denmark | 1993 | 51,098832 | 0,53 | 1,06 | 1,05777944 | 0,76 | 0,76109989 | 1,00 |
| Denmark | 1994 | 48,2433763 | 0,79 | 1,05 | 1,04530344 | 0,76 | 0,76195113 | 1,00 |
| Denmark | 1995 | 45,9606724 | 0,84 |  | 1,04350731 | 0,76 | 0,75987483 | 1,00 |
| Denmark | 1996 | 45,1188857 | 0,68 |  | 1,04171119 | 0,76 | 0,75853301 | 1,00 |
| Denmark | 1997 | 40,9368353 | 0,57 |  | 1,03991506 | 0,76 | 0,75839767 | 1,00 |
| Denmark | 1998 | 40,4443395 | 0,52 | 1,04 | 1,03811893 | 0,76 | 0,75713056 | 1,00 |
| Denmark | 1999 | 40,6253073 | 0,51 |  | 1,03154829 | 0,75 | 0,75372182 | 1,00 |
| Denmark | 2000 | 40,4732651 | 0,49 | 1,02 | 1,02497765 | 0,75 | 0,75356694 | 1,00 |
| Denmark | 2001 | 39,7621883 | 0,49 |  | 1,06922845 | 0,75 | 0,75122322 | 1,00 |
| Denmark | 2002 | 26,8323423 | 0,50 | 1,11 | 1,11347926 | 0,75 | 0,74992827 | 1,00 |
| Denmark | 2003 | 26,6772185 | 0,57 |  | 1,10267056 | 0,75 | 0,74844952 | 1,00 |
| Denmark | 2004 | 26,9795353 | 0,57 |  | 1,09186185 | 0,74 | 0,7415579 | 1,00 |
| Denmark | 2005 | 26,6270601 | 0,55 |  | 1,08105314 | 0,78 | 0,77633868 | 1,00 |
| Denmark | 2006 | 26,2407706 | 0,53 | 1,07 | 1,07024443 | 0,77 | 0,77257161 | 1,00 |
| Denmark | 2007 | 25,8557149 | 0,52 |  | 1,07129707 | 0,76 | 0,76272334 | 1,00 |
| Denmark | 2008 | 25,5852649 | 0,52 | 1,07 | 1,07234971 | 0,76 | 0,75509485 | 1,00 |
| Denmark | 2009 | 25,5947676 | 0,58 |  | 1,07774882 | 0,75 | 0,74594084 | 1,00 |
| Denmark | 2010 | 25,5947676 | 0,56 |  | 1,08314792 | 0,76 | 0,764197 | 1,00 |
| Denmark | 2011 |  | 0,54 | 1,09 | 1,08854703 | 0,74 | 0,74384701 | 1,00 |
| Denmark | 2012 |  | 0,49 |  | 1,10065078 | 0,71 | 0,71082932 | 1,00 |
| Denmark | 2013 |  | 0,48 | 1,11 | 1,11275453 | 0,70 | 0,70178572 | 1,00 |
| Denmark | 2014 |  | 0,48 | 1,11 | 1,10728666 | 0,70 | 0,69697685 | 1,00 |
| Denmark | 2015 |  | 0,47 |  | 1,10728666 | 0,70 | 0,69735639 | 1,00 |
| Denmark | 2016 |  | 0,50 |  | 1,10728666 | 0,69 | 0,69130881 | 1,00 |
| Denmark | 2017 |  | 0,49 |  | 1,10728666 | 0,69 | 0,68998594 | 1,00 |
| Denmark | 2018 |  |  |  | 1,10728666 | 0,67 | 0,66708606 | 1,00 |
| Denmark | 2019 |  |  |  | 1,10728666 |  | 0,66708606 |  |
| Estonia | 1980 |  |  |  | 1,44716654 |  |  |  |
| Estonia | 1981 |  |  |  | 1,44716654 |  |  |  |
| Estonia | 1982 |  |  |  | 1,44716654 |  |  |  |
| Estonia | 1983 |  |  |  | 1,44716654 |  |  |  |
| Estonia | 1984 |  |  |  | 1,44716654 |  |  |  |
| Estonia | 1985 |  |  |  | 1,44716654 |  |  |  |
| Estonia | 1986 |  |  |  | 1,44716654 |  |  |  |
| Estonia | 1987 |  |  |  | 1,44716654 |  |  |  |
| Estonia | 1988 |  |  |  | 1,44716654 |  |  |  |
| Estonia | 1989 |  |  |  | 1,44716654 |  |  |  |
| Estonia | 1990 |  |  |  | 1,44716654 |  |  |  |
| Estonia | 1991 |  |  |  | 1,44716654 |  |  | 0,00 |
| Estonia | 1992 |  |  |  | 1,44716654 |  |  | 0,00 |
| Estonia | 1993 |  |  |  | 1,44716654 |  |  | 0,00 |
| Estonia | 1994 |  |  |  | 1,44716654 |  |  | 0,00 |
| Estonia | 1995 |  |  |  | 1,44716654 |  |  | 0,00 |
| Estonia | 1996 |  |  |  | 1,44716654 |  |  | 0,00 |
| Estonia | 1997 |  |  |  | 1,44716654 |  |  | 0,00 |
| Estonia | 1998 |  |  |  | 1,44716654 |  |  | 0,00 |
| Estonia | 1999 |  | 0,41 | 1,45 | 1,44716654 |  |  | 0,50 |
| Estonia | 2000 |  | 0,55 | 1,43 | 1,42780697 |  |  | 0,50 |
| Estonia | 2001 |  | 0,50 | 1,73 | 1,73417616 |  |  | 0,50 |
| Estonia | 2002 |  | 0,47 | 2,01 | 2,00659258 |  |  | 0,50 |
| Estonia | 2003 |  | 0,45 | 1,93 | 1,92792758 |  |  | 0,50 |
| Estonia | 2004 |  | 0,68 | 1,87 | 1,86571912 |  |  | 0,50 |
| Estonia | 2005 |  | 0,67 | 1,74 | 1,74449541 |  |  | 0,50 |
| Estonia | 2006 |  | 0,75 | 1,76 | 1,76338063 |  |  | 0,50 |
| Estonia | 2007 |  | 0,75 | 1,56 | 1,56212181 |  |  | 0,50 |
| Estonia | 2008 |  | 1,09 | 1,58 | 1,5762649 |  |  | 0,50 |
| Estonia | 2009 |  | 1,51 | 1,31 | 1,31107121 |  |  | 0,50 |
| Estonia | 2010 |  | 1,55 | 1,43 | 1,43142106 |  |  | 0,50 |
| Estonia | 2011 |  | 1,33 | 1,35 | 1,35096593 |  |  | 0,50 |
| Estonia | 2012 |  | 1,17 | 1,51 | 1,5106206 |  |  | 0,50 |
| Estonia | 2013 |  | 1,10 | 1,43 | 1,42876305 |  |  | 0,50 |
| Estonia | 2014 |  | 1,10 | 1,29 | 1,2912813 |  |  | 0,50 |
| Estonia | 2015 |  | 1,15 |  | 1,2912813 |  |  | 0,50 |
| Estonia | 2016 |  | 1,24 |  | 1,2912813 |  |  | 0,50 |
| Estonia | 2017 |  | 1,22 |  | 1,2912813 |  |  | 0,50 |
| Estonia | 2018 |  |  |  | 1,2912813 |  |  | 0,50 |
| Estonia | 2019 |  |  |  | 1,2912813 |  |  |  |
| Finland | 1980 | 15,2683162 | 0,26 |  | 1,04 | 0,68 | 0,68371208 | 0,50 |
| Finland | 1981 | 15,9790482 | 0,25 |  | 1,04 | 0,69 | 0,68680531 | 0,50 |
| Finland | 1982 | 15,9790482 | 0,55 | 1,04 | 1,04 | 0,68 | 0,68057066 | 0,50 |
| Finland | 1983 | 24,7453514 | 0,61 |  | 1,03833333 | 0,68 | 0,67755077 | 0,50 |
| Finland | 1984 | 24,7453514 | 0,58 |  | 1,03666667 | 0,68 | 0,67681067 | 0,50 |
| Finland | 1985 | 88,9138337 | 0,66 |  | 1,035 | 0,67 | 0,67448453 | 0,50 |
| Finland | 1986 | 88,9138337 | 0,67 |  | 1,03333333 | 0,67 | 0,67377666 | 0,50 |
| Finland | 1987 | 88,9138337 | 0,76 |  | 1,03166667 | 0,67 | 0,67416206 | 0,50 |
| Finland | 1988 | 88,9138337 | 0,77 | 1,03 | 1,03 | 0,68 | 0,67506895 | 0,50 |
| Finland | 1989 | 88,9672867 | 0,82 |  | 1,04142857 | 0,67 | 0,66959528 | 0,50 |
| Finland | 1990 | 89,0207398 | 1,00 |  | 1,05285714 | 0,67 | 0,66630212 | 0,50 |
| Finland | 1991 | 89,0741929 | 1,22 |  | 1,06428571 | 0,66 | 0,66343924 | 0,50 |
| Finland | 1992 | 89,1276459 | 1,40 |  | 1,07571429 | 0,64 | 0,6418138 | 0,50 |
| Finland | 1993 | 89,181099 | 1,31 |  | 1,08714286 | 0,68 | 0,68134488 | 0,50 |
| Finland | 1994 | 89,2345521 | 1,20 |  | 1,09857143 | 0,69 | 0,68520619 | 0,50 |
| Finland | 1995 | 90,5708788 | 1,04 | 1,11 | 1,11 | 0,68 | 0,67741615 | 0,50 |
| Finland | 1996 | 90,6243318 | 0,80 |  | 1,11166667 | 0,68 | 0,67889417 | 0,50 |
| Finland | 1997 | 83,7823391 | 0,73 |  | 1,11333333 | 0,68 | 0,67683927 | 0,50 |
| Finland | 1998 | 83,7823391 | 0,70 |  | 1,115 | 0,67 | 0,6701896 | 0,50 |
| Finland | 1999 | 83,5685268 | 0,66 |  | 1,11666667 | 0,67 | 0,67061626 | 0,50 |
| Finland | 2000 | 83,4078816 | 0,62 |  | 1,11833333 | 0,68 | 0,67546815 | 0,50 |
| Finland | 2001 | 83,2472363 | 0,59 | 1,12 | 1,12 | 0,68 | 0,67952295 | 0,50 |
| Finland | 2002 | 83,0865911 | 0,58 |  | 1,12333333 | 0,68 | 0,67921455 | 0,50 |
| Finland | 2003 | 82,9878394 | 0,60 |  | 1,12666667 | 0,68 | 0,68055879 | 0,50 |
| Finland | 2004 | 82,8890877 | 0,60 |  | 1,13 | 0,70 | 0,70086477 | 0,50 |
| Finland | 2005 | 82,7305346 | 0,61 |  | 1,13333333 | 0,70 | 0,70123583 | 0,50 |
| Finland | 2006 | 82,5719816 | 0,61 |  | 1,13666667 | 0,70 | 0,70041593 | 0,50 |
| Finland | 2007 | 82,8062608 | 0,61 | 1,14 | 1,14 | 0,70 | 0,69995658 | 0,50 |
| Finland | 2008 | 82,6949778 | 0,61 |  | 1,1425 | 0,70 | 0,70232345 | 0,50 |
| Finland | 2009 | 82,7745275 | 0,71 |  | 1,145 | 0,70 | 0,70219231 | 0,50 |
| Finland | 2010 | 82,6736582 | 0,72 |  | 1,1475 | 0,71 | 0,70951582 | 0,50 |
| Finland | 2011 |  | 0,69 | 1,15 | 1,15 | 0,71 | 0,71164904 | 0,50 |
| Finland | 2012 |  | 0,70 |  | 1,15 | 0,72 | 0,72045581 | 0,50 |
| Finland | 2013 |  | 0,70 |  | 1,15 | 0,73 | 0,72674533 | 0,50 |
| Finland | 2014 |  | 0,69 |  | 1,15 | 0,73 | 0,73125292 | 0,50 |
| Finland | 2015 |  | 0,67 |  | 1,15 | 0,73 | 0,73299434 | 0,50 |
| Finland | 2016 |  | 0,65 |  | 1,15 | 0,74 | 0,73596576 | 0,50 |
| Finland | 2017 |  | 0,58 |  | 1,15 | 0,76 | 0,75685345 | 0,50 |
| Finland | 2018 |  |  |  | 1,15 | 0,76 | 0,75983963 | 0,50 |
| Finland | 2019 |  |  |  | 1,15 |  | 0,75983963 |  |
| France | 1980 | 40,5650662 | 0,14 |  | 0,84 | 0,25 | 0,2504636 | 0,00 |
| France | 1981 | 40,5650662 | 0,33 |  | 0,84 | 0,25 | 0,2500808 | 0,00 |
| France | 1982 | 40,5650662 | 0,31 |  | 0,84 | 0,25 | 0,24661152 | 0,00 |
| France | 1983 | 40,5650662 | 0,27 |  | 0,84 | 0,25 | 0,24576801 | 0,00 |
| France | 1984 | 40,5650662 | 0,27 |  | 0,84 | 0,25 | 0,24684879 | 0,00 |
| France | 1985 | 49,3313694 | 0,24 |  | 0,84 | 0,25 | 0,2477093 | 0,00 |
| France | 1986 | 49,3313694 | 0,22 |  | 0,84 | 0,24 | 0,24431899 | 0,00 |
| France | 1987 | 58,5708861 | 0,29 |  | 0,84 | 0,25 | 0,24625347 | 0,00 |
| France | 1988 | 58,5708861 | 0,35 |  | 0,84 | 0,30 | 0,30424563 | 0,50 |
| France | 1989 | 58,5403415 | 0,30 |  | 0,84 | 0,31 | 0,30916362 | 0,50 |
| France | 1990 | 58,5097968 | 0,29 |  | 0,84 | 0,31 | 0,30836164 | 0,50 |
| France | 1991 | 57,1963786 | 0,30 |  | 0,84 | 0,31 | 0,30852709 | 0,00 |
| France | 1992 | 57,165834 | 0,29 |  | 0,84 | 0,31 | 0,31119105 | 0,00 |
| France | 1993 | 57,1352894 | 0,29 |  | 0,84 | 0,31 | 0,30533647 | 0,00 |
| France | 1994 | 57,1047448 | 0,28 |  | 0,84 | 0,32 | 0,3164843 | 0,00 |
| France | 1995 | 57,0742002 | 0,32 |  | 0,84 | 0,31 | 0,31492257 | 0,50 |
| France | 1996 | 56,8952777 | 0,36 |  | 0,84 | 0,32 | 0,31853077 | 0,50 |
| France | 1997 | 60,3046442 | 0,40 |  | 0,84 | 0,32 | 0,31932812 | 0,00 |
| France | 1998 | 60,2930142 | 0,40 |  | 0,84 | 0,32 | 0,32110629 | 0,00 |
| France | 1999 | 60,2813842 | 0,39 |  | 0,84 | 0,32 | 0,32362202 | 0,00 |
| France | 2000 | 58,8584267 | 0,38 |  | 0,84 | 0,33 | 0,32686939 | 0,00 |
| France | 2001 | 58,554439 | 0,38 | 0,84 | 0,84 | 0,33 | 0,3287855 | 0,00 |
| France | 2002 | 58,1941488 | 0,39 |  | 0,846 | 0,33 | 0,3302365 | 0,00 |
| France | 2003 | 58,012068 | 0,39 |  | 0,852 | 0,33 | 0,32998472 | 0,50 |
| France | 2004 | 58,2904922 | 0,37 |  | 0,858 | 0,32 | 0,32463362 | 0,50 |
| France | 2005 | 58,3998362 | 0,34 |  | 0,864 | 0,33 | 0,32587474 | 0,00 |
| France | 2006 | 57,7664978 | 0,32 | 0,87 | 0,87 | 0,32 | 0,32436747 | 0,00 |
| France | 2007 | 57,753648 | 0,30 |  | 0,88 | 0,32 | 0,32363849 | 0,00 |
| France | 2008 | 57,6301121 | 0,30 | 0,89 | 0,89 | 0,32 | 0,32308144 | 0,00 |
| France | 2009 | 57,6315523 | 0,32 |  | 0,87333333 | 0,32 | 0,32378837 | 0,00 |
| France | 2010 | 57,4678545 | 0,31 |  | 0,85666667 | 0,32 | 0,32438826 | 0,00 |
| France | 2011 |  | 0,30 | 0,84 | 0,84 | 0,32 | 0,32472859 | 0,00 |
| France | 2012 |  | 0,29 |  | 0,83666667 | 0,32 | 0,32415344 | 0,00 |
| France | 2013 |  | 0,29 |  | 0,83333333 | 0,32 | 0,32381414 | 0,00 |
| France | 2014 |  | 0,28 | 0,83 | 0,83 | 0,32 | 0,32358097 | 0,00 |
| France | 2015 |  | 0,25 |  | 0,69 | 0,32 | 0,32378917 | 0,00 |
| France | 2016 |  | 0,25 |  | 0,55 | 0,32 | 0,3239858 | 0,00 |
| France | 2017 |  | 0,22 | 0,41 | 0,41 | 0,32 | 0,31617336 | 0,00 |
| France | 2018 |  | 0,21 | 0,41 | 0,41 | 0,32 | 0,31606392 | 0,00 |
| France | 2019 |  |  |  | 0,41 |  | 0,31606392 |  |
| Germany | 1980 | 17,7683014 | 0,11 | 0,43 | 0,43227092 | 0,36 | 0,35552778 | 0,50 |
| Germany | 1981 | 17,7683014 | 0,11 | 0,44 | 0,43762781 | 0,36 | 0,35552778 | 0,50 |
| Germany | 1982 | 17,7683014 | 0,11 | 0,44 | 0,44282744 | 0,36 | 0,35552778 | 0,50 |
| Germany | 1983 | 17,7683014 | 0,10 | 0,45 | 0,45031712 | 0,36 | 0,35552778 | 0,50 |
| Germany | 1984 | 17,7683014 | 0,07 | 0,46 | 0,45647558 | 0,36 | 0,35552778 | 0,50 |
| Germany | 1985 | 17,7683014 | 0,07 | 0,46 | 0,46351931 | 0,36 | 0,35552778 | 0,50 |
| Germany | 1986 | 36,0012683 | 0,13 | 0,51 | 0,50663717 | 0,36 | 0,35777088 | 0,50 |
| Germany | 1987 | 36,0012683 | 0,20 | 0,47 | 0,47494553 | 0,36 | 0,36 | 0,50 |
| Germany | 1988 | 38,887734 | 0,20 | 0,48 | 0,48148148 | 0,36 | 0,36221541 | 0,50 |
| Germany | 1989 | 42,8793903 | 0,22 | 0,49 | 0,48796499 | 0,36 | 0,36441734 | 0,50 |
| Germany | 1990 | 38,5323679 | 0,23 | 0,66 | 0,65864412 | 0,37 | 0,36551334 | 0,50 |
| Germany | 1991 | 42,4319345 | 0,24 | 0,64 | 0,63571492 | 0,35 | 0,34871192 | 0,50 |
| Germany | 1992 | 56,0773973 | 0,27 | 0,60 | 0,60418903 | 0,35 | 0,34871192 | 0,50 |
| Germany | 1993 | 55,863585 | 0,25 | 0,61 | 0,60720852 | 0,35 | 0,34985711 | 0,50 |
| Germany | 1994 | 55,863585 | 0,23 | 0,57 | 0,56976966 | 0,35 | 0,35099858 | 0,50 |
| Germany | 1995 | 55,6497728 | 0,24 | 0,58 | 0,58064094 | 0,35 | 0,35213634 | 0,50 |
| Germany | 1996 | 54,1582001 | 0,23 | 0,57 | 0,56991021 | 0,35 | 0,35099858 | 0,50 |
| Germany | 1997 | 54,1065774 | 0,23 | 0,57 | 0,56516404 | 0,35 | 0,34985711 | 0,50 |
| Germany | 1998 | 54,0549548 | 0,23 |  | 0,55891334 | 0,35 | 0,35099858 | 0,50 |
| Germany | 1999 | 54,0033322 | 0,22 |  | 0,55266263 | 0,37 | 0,37416574 | 1,00 |
| Germany | 2000 | 53,9517096 | 0,21 | 0,55 | 0,54641193 | 0,38 | 0,37841776 | 1,00 |
| Germany | 2001 | 53,900087 | 0,19 |  | 0,53893214 | 0,39 | 0,39089641 | 1,00 |
| Germany | 2002 | 53,8484644 | 0,19 | 0,53 | 0,53145236 | 0,46 | 0,45956501 | 0,50 |
| Germany | 2003 | 53,7968417 | 0,19 |  | 0,54491722 | 0,46 | 0,45956501 | 0,50 |
| Germany | 2004 | 53,7452191 | 0,17 |  | 0,55838208 | 0,46 | 0,4586938 | 0,50 |
| Germany | 2005 | 53,7211861 | 0,19 |  | 0,57184693 | 0,46 | 0,4586938 | 0,50 |
| Germany | 2006 | 53,7077683 | 0,18 | 0,59 | 0,58531179 | 0,46 | 0,45782093 | 0,50 |
| Germany | 2007 | 56,3624803 | 0,21 |  | 0,59249241 | 0,46 | 0,4613025 | 0,50 |
| Germany | 2008 | 56,3624803 | 0,25 |  | 0,59967304 | 0,46 | 0,4621688 | 0,50 |
| Germany | 2009 | 56,3624803 | 0,25 | 0,61 | 0,60685366 | 0,46 | 0,4621688 | 0,50 |
| Germany | 2010 |  | 0,23 | 0,65 | 0,6491674 | 0,46 | 0,4613025 | 0,50 |
| Germany | 2011 |  | 0,20 | 0,65 | 0,65371734 | 0,45 | 0,45254834 | 0,50 |
| Germany | 2012 |  | 0,20 |  | 0,6247778 | 0,45 | 0,45254834 | 0,50 |
| Germany | 2013 |  | 0,21 | 0,60 | 0,59583826 | 0,46 | 0,45607017 | 0,50 |
| Germany | 2014 |  | 0,23 | 0,59 | 0,59100899 | 0,46 | 0,45607017 | 0,50 |
| Germany | 2015 |  | 0,25 |  | 0,59100899 | 0,46 | 0,45607017 | 0,50 |
| Germany | 2016 |  | 0,24 |  | 0,59100899 | 0,46 | 0,46043458 | 0,50 |
| Germany | 2017 |  | 0,23 |  | 0,59100899 | 0,47 | 0,46561787 | 0,50 |
| Germany | 2018 |  |  |  | 0,59100899 | 0,47 | 0,46733286 |  |
| Germany | 2019 |  |  |  | 0,59100899 |  | 0,46733286 |  |
| Greece | 1980 | 12,8228098 | 0,03 |  | 0,75 | 0,37 | 0,3660163 | 0,00 |
| Greece | 1981 | 12,8228098 | 0,04 |  | 0,75 |  | 0,36564902 | 0,00 |
| Greece | 1982 | 12,8228098 | 0,04 |  | 0,75 |  | 0,36528173 | 0,00 |
| Greece | 1983 | 12,8228098 | 0,04 |  | 0,75 | 0,36 | 0,36491445 | 0,00 |
| Greece | 1984 | 17,4425682 | 0,04 |  | 0,75 |  | 0,3649296 | 0,00 |
| Greece | 1985 | 17,4425682 | 0,05 |  | 0,75 |  | 0,36494475 | 0,00 |
| Greece | 1986 | 17,4425682 | 0,05 |  | 0,75 | 0,36 | 0,36495991 | 0,00 |
| Greece | 1987 | 17,4425682 | 0,04 |  | 0,75 |  | 0,36592907 | 0,00 |
| Greece | 1988 | 17,4425682 | 0,04 |  | 0,75 |  | 0,36689823 | 0,00 |
| Greece | 1989 | 17,4425682 | 0,04 |  | 0,75 | 0,37 | 0,36786739 | 0,00 |
| Greece | 1990 | 17,4425682 | 0,08 |  | 0,75 |  | 0,36662684 | 0,00 |
| Greece | 1991 | 17,9756172 | 0,08 |  | 0,75 |  | 0,3653863 | 0,00 |
| Greece | 1992 | 17,9756172 | 0,07 |  | 0,75 | 0,36 | 0,36414575 | 0,50 |
| Greece | 1993 | 17,9756172 | 0,07 |  | 0,75 |  | 0,36483107 | 0,50 |
| Greece | 1994 | 17,9756172 | 0,07 |  | 0,75 |  | 0,3655164 | 0,50 |
| Greece | 1995 | 18,1533002 | 0,08 |  | 0,75 | 0,37 | 0,36620172 | 0,50 |
| Greece | 1996 | 18,1533002 | 0,08 |  | 0,75 |  | 0,36655206 | 0,50 |
| Greece | 1997 | 18,1533002 | 0,07 |  | 0,75 |  | 0,3669024 | 0,50 |
| Greece | 1998 | 18,1533002 | 0,07 |  | 0,75 | 0,37 | 0,36725275 | 0,50 |
| Greece | 1999 | 18,1533002 | 0,08 |  | 0,75 |  | 0,3703997 | 0,50 |
| Greece | 2000 | 18,1533002 | 0,17 | 0,75 | 0,75 |  | 0,37354664 | 0,50 |
| Greece | 2001 | 18,1533002 | 0,17 |  | 0,66 | 0,38 | 0,37669359 | 0,50 |
| Greece | 2002 | 18,3309832 | 0,18 | 0,57 | 0,57 |  | 0,37740347 | 0,50 |
| Greece | 2003 | 18,3309832 | 0,17 |  | 0,59 |  | 0,37811335 | 0,50 |
| Greece | 2004 | 18,3309832 | 0,18 |  | 0,61 | 0,38 | 0,37882323 | 0,50 |
| Greece | 2005 | 18,3309832 | 0,19 |  | 0,63 |  | 0,38028355 | 0,50 |
| Greece | 2006 | 18,3309832 | 0,19 | 0,65 | 0,65 |  | 0,38174387 | 0,50 |
| Greece | 2007 | 18,3309832 | 0,19 |  | 0,615 | 0,38 | 0,38320419 | 0,50 |
| Greece | 2008 | 18,3309832 | 0,21 | 0,58 | 0,58 |  | 0,38293046 | 0,50 |
| Greece | 2009 | 18,3309832 | 0,26 |  | 0,575 |  | 0,38265673 | 0,50 |
| Greece | 2010 | 18,3309832 | 0,26 | 0,57 | 0,57 | 0,38 | 0,38238299 | 0,50 |
| Greece | 2011 |  | 0,25 |  | 0,57 |  | 0,36111981 | 0,50 |
| Greece | 2012 |  | 0,24 |  | 0,57 |  | 0,33985662 | 0,50 |
| Greece | 2013 |  | 0,23 |  | 0,57 | 0,32 | 0,31859344 | 0,50 |
| Greece | 2014 |  | 0,26 |  | 0,57 |  | 0,318755 | 0,50 |
| Greece | 2015 |  | 0,24 |  | 0,57 |  | 0,31891656 | 0,50 |
| Greece | 2016 |  | 0,25 |  | 0,57 | 0,32 | 0,31907812 | 0,50 |
| Greece | 2017 |  | 0,20 |  | 0,57 |  | 0,31907812 | 0,50 |
| Greece | 2018 |  |  |  | 0,57 |  | 0,31907812 | 0,50 |
| Greece | 2019 |  |  |  | 0,57 |  | 0,31907812 |  |
| Hungary | 1980 |  |  |  | 1,23 |  | 0,34219998 |  |
| Hungary | 1981 |  |  |  | 1,23 |  | 0,34219998 |  |
| Hungary | 1982 |  |  |  | 1,23 |  | 0,34219998 |  |
| Hungary | 1983 |  |  |  | 1,23 |  | 0,34219998 |  |
| Hungary | 1984 |  |  |  | 1,23 |  | 0,34219998 |  |
| Hungary | 1985 |  |  |  | 1,23 |  | 0,34219998 |  |
| Hungary | 1986 |  |  |  | 1,23 |  | 0,34219998 |  |
| Hungary | 1987 |  |  |  | 1,23 |  | 0,34219998 |  |
| Hungary | 1988 |  |  |  | 1,23 |  | 0,34219998 |  |
| Hungary | 1989 |  |  |  | 1,23 |  | 0,34219998 |  |
| Hungary | 1990 |  |  |  | 1,23 | 0,34 | 0,34219998 | 0,00 |
| Hungary | 1991 |  |  |  | 1,23 |  | 0,33184877 | 0,00 |
| Hungary | 1992 |  |  |  | 1,23 |  | 0,32149756 | 0,00 |
| Hungary | 1993 |  |  |  | 1,23 |  | 0,31114635 | 0,00 |
| Hungary | 1994 |  |  |  | 1,23 |  | 0,30079514 | 0,00 |
| Hungary | 1995 |  |  |  | 1,23 | 0,29 | 0,29044393 | 0,00 |
| Hungary | 1996 |  |  |  | 1,23 |  | 0,28597102 | 0,00 |
| Hungary | 1997 |  |  |  | 1,23 |  | 0,28149811 | 0,00 |
| Hungary | 1998 |  |  |  | 1,23 | 0,28 | 0,2770252 | 0,00 |
| Hungary | 1999 |  | 0,45 |  | 1,23 |  | 0,28258497 | 0,00 |
| Hungary | 2000 |  | 0,52 |  | 1,23 |  | 0,28814474 | 0,00 |
| Hungary | 2001 |  | 0,55 |  | 1,23 | 0,29 | 0,29370452 | 0,00 |
| Hungary | 2002 |  | 0,56 | 1,23 | 1,23 |  | 0,29666396 | 0,00 |
| Hungary | 2003 |  | 0,77 |  | 1,216 | 0,30 | 0,2996234 | 0,00 |
| Hungary | 2004 |  | 0,60 |  | 1,202 |  | 0,30173827 | 0,50 |
| Hungary | 2005 |  | 0,63 |  | 1,188 | 0,30 | 0,30385314 | 0,50 |
| Hungary | 2006 |  | 0,70 |  | 1,174 |  | 0,30329503 | 0,50 |
| Hungary | 2007 |  | 0,71 | 1,16 | 1,16 | 0,30 | 0,30273692 | 0,50 |
| Hungary | 2008 |  | 0,73 |  | 1,14333333 | 0,30 | 0,30145642 | 0,50 |
| Hungary | 2009 |  | 0,79 |  | 1,12666667 |  | 0,30189124 | 0,50 |
| Hungary | 2010 |  | 0,77 |  | 1,11 |  | 0,30232606 | 0,00 |
| Hungary | 2011 |  | 0,72 |  | 1,09333333 |  | 0,30276088 | 0,00 |
| Hungary | 2012 |  | 0,72 |  | 1,07666667 | 0,30 | 0,3031957 | 0,00 |
| Hungary | 2013 |  | 0,69 | 1,06 | 1,06 |  | 0,35347502 | 0,00 |
| Hungary | 2014 |  | 0,68 |  | 1,06 | 0,40 | 0,40375434 | 0,00 |
| Hungary | 2015 |  | 0,68 |  | 1,06 |  | 0,38365027 | 0,00 |
| Hungary | 2016 |  | 0,71 |  | 1,06 | 0,36 | 0,3635462 | 0,00 |
| Hungary | 2017 |  | 0,73 |  | 1,06 |  | 0,36278523 | 0,00 |
| Hungary | 2018 |  | 0,73 |  | 1,06 | 0,36 | 0,36202425 | 0,00 |
| Hungary | 2019 |  |  |  | 1,06 |  | 0,36202425 |  |
| Iceland | 1980 |  |  |  | 1,11 | 0,76 | 0,75530003 |  |
| Iceland | 1981 |  |  |  | 1,11 | 0,54 | 0,54051502 |  |
| Iceland | 1982 |  |  |  | 1,11 | 0,53 | 0,53469478 |  |
| Iceland | 1983 |  |  |  | 1,11 | 0,52 | 0,522951 |  |
| Iceland | 1984 |  |  |  | 1,11 | 0,53 | 0,52583721 |  |
| Iceland | 1985 |  |  |  | 1,11 | 0,52 | 0,51977018 |  |
| Iceland | 1986 |  |  |  | 1,11 | 0,53 | 0,52673734 |  |
| Iceland | 1987 |  |  |  | 1,11 | 0,73 | 0,72524745 |  |
| Iceland | 1988 |  |  |  | 1,11 | 0,73 | 0,72591066 |  |
| Iceland | 1989 |  |  |  | 1,11 | 0,76 | 0,75532926 |  |
| Iceland | 1990 |  | 0,30 |  | 1,11 | 0,81 | 0,80795479 |  |
| Iceland | 1991 |  | 0,28 |  | 1,11 | 0,81 | 0,80604241 |  |
| Iceland | 1992 |  | 0,29 |  | 1,11 | 0,83 | 0,82583789 |  |
| Iceland | 1993 |  | 0,29 |  | 1,11 | 0,82 | 0,82375919 |  |
| Iceland | 1994 |  | 0,26 |  | 1,11 | 0,82 | 0,82290104 |  |
| Iceland | 1995 |  | 0,25 |  | 1,11 | 0,82 | 0,82140659 |  |
| Iceland | 1996 |  | 0,37 |  | 1,11 | 0,82 | 0,82018824 |  |
| Iceland | 1997 |  | 0,35 |  | 1,11 | 0,82 | 0,81934213 |  |
| Iceland | 1998 |  | 0,35 |  | 1,11 | 0,82 | 0,81735489 |  |
| Iceland | 1999 |  | 0,35 |  | 1,11 | 0,81 | 0,81033859 |  |
| Iceland | 2000 |  | 0,37 |  | 1,11 | 0,81 | 0,81045736 |  |
| Iceland | 2001 |  | 0,41 | 1,11 | 1,11 | 0,81 | 0,81061547 |  |
| Iceland | 2002 |  | 0,53 | 1,08 | 1,08 | 0,81 | 0,81090942 |  |
| Iceland | 2003 |  | 0,64 | 1,10 | 1,1 | 0,81 | 0,81108238 |  |
| Iceland | 2004 |  | 0,68 | 1,11 | 1,11014851 | 0,82 | 0,81815608 |  |
| Iceland | 2005 |  | 0,62 | 1,08 | 1,08078335 | 0,82 | 0,81838116 |  |
| Iceland | 2006 |  | 0,57 | 1,07 | 1,0746634 | 0,82 | 0,81877654 |  |
| Iceland | 2007 |  | 0,58 | 1,03 | 1,03151515 | 0,83 | 0,82589642 |  |
| Iceland | 2008 |  | 0,61 | 1,05 | 1,04528764 | 0,83 | 0,82772203 |  |
| Iceland | 2009 |  | 0,63 | 1,05 | 1,04539877 | 0,60 | 0,6000682 |  |
| Iceland | 2010 |  | 0,55 | 1,05 | 1,04523227 | 0,60 | 0,60033166 |  |
| Iceland | 2011 |  | 0,46 | 1,05 | 1,04700353 | 0,60 | 0,6001653 |  |
| Iceland | 2012 |  | 0,42 | 1,07 | 1,06574394 | 0,29 | 0,29325757 |  |
| Iceland | 2013 |  | 0,42 | 1,07 | 1,06531532 | 0,61 | 0,60738913 |  |
| Iceland | 2014 |  | 0,42 | 1,08 | 1,07931034 | 0,30 | 0,29664794 |  |
| Iceland | 2015 |  | 0,41 |  | 1,07931034 | 0,30 | 0,29664794 |  |
| Iceland | 2016 |  | 0,37 |  | 1,07931034 | 0,61 | 0,60698745 |  |
| Iceland | 2017 |  | 0,41 |  | 1,07931034 | 0,61 | 0,60695357 |  |
| Iceland | 2018 |  |  |  | 1,07931034 |  | 0,60695357 |  |
| Iceland | 2019 |  |  |  | 1,07931034 |  | 0,60695357 |  |
| Ireland | 1980 | 7,26369071 | 0,06 | 1,08 | 1,08445298 | 0,42 | 0,42305982 | 0,00 |
| Ireland | 1981 | 19,592544 | 0,07 | 1,12 | 1,11637081 | 0,43 | 0,42874023 | 0,00 |
| Ireland | 1982 | 19,592544 | 0,09 | 1,11 | 1,11065574 | 0,43 | 0,42515528 | 0,00 |
| Ireland | 1983 | 19,592544 | 0,09 | 1,07 | 1,07158351 | 0,42 | 0,42381452 | 0,00 |
| Ireland | 1984 | 17,4544213 | 0,08 | 1,06 | 1,06026786 | 0,42 | 0,42459925 | 0,00 |
| Ireland | 1985 | 17,4544213 | 0,09 | 1,08 | 1,08370044 | 0,43 | 0,425757 | 0,00 |
| Ireland | 1986 | 17,4544213 | 0,09 | 1,10 | 1,0955414 | 0,43 | 0,42755572 | 0,00 |
| Ireland | 1987 | 17,4544213 | 0,09 | 1,12 | 1,12033195 | 0,43 | 0,42780694 | 0,00 |
| Ireland | 1988 | 17,4544213 | 0,07 | 1,10 | 1,09766454 | 0,43 | 0,4275613 | 0,00 |
| Ireland | 1989 | 17,4544213 | 0,07 | 0,95 | 0,94805195 | 0,43 | 0,42771367 | 0,00 |
| Ireland | 1990 | 17,4544213 | 0,07 | 0,94 | 0,93973214 | 0,43 | 0,4273368 | 0,00 |
| Ireland | 1991 | 17,4544213 | 0,07 | 0,94 | 0,93630573 | 0,43 | 0,42669435 | 0,00 |
| Ireland | 1992 | 17,4544213 | 0,07 | 0,92 | 0,91810345 | 0,43 | 0,42557693 | 0,00 |
| Ireland | 1993 | 17,4544213 | 0,06 | 0,94 | 0,94091904 | 0,49 | 0,48543158 | 0,00 |
| Ireland | 1994 | 17,4544213 | 0,06 | 0,94 | 0,94022989 | 0,49 | 0,48545401 | 0,50 |
| Ireland | 1995 | 17,4544213 | 0,06 |  | 0,93130307 | 0,49 | 0,48654044 | 0,50 |
| Ireland | 1996 | 17,4544213 | 0,06 |  | 0,92237625 | 0,48 | 0,48402873 | 0,50 |
| Ireland | 1997 | 17,4544213 | 0,06 |  | 0,91344943 | 0,48 | 0,48411405 | 0,50 |
| Ireland | 1998 | 22,4295457 | 0,06 | 0,90 | 0,90452261 | 0,48 | 0,48298784 | 0,50 |
| Ireland | 1999 | 22,4295457 | 0,06 | 0,91 | 0,9071618 | 0,48 | 0,48376142 | 0,50 |
| Ireland | 2000 | 22,4295457 | 0,05 | 0,92 | 0,92307692 | 0,48 | 0,4839297 | 1,00 |
| Ireland | 2001 | 22,4295457 | 0,06 | 0,98 | 0,98280802 | 0,49 | 0,48514052 | 1,00 |
| Ireland | 2002 | 23,1402777 | 0,07 | 1,02 | 1,01757227 | 0,49 | 0,48507125 | 1,00 |
| Ireland | 2003 | 23,1402777 | 0,07 | 1,02 | 1,02414557 | 0,49 | 0,4856165 | 1,00 |
| Ireland | 2004 | 23,1402777 | 0,08 | 1,04 | 1,041862 | 0,49 | 0,48623027 | 1,00 |
| Ireland | 2005 | 24,2093391 | 0,08 | 1,06 | 1,05814296 | 0,49 | 0,48613875 | 1,00 |
| Ireland | 2006 | 25,2784004 | 0,10 | 1,07 | 1,07306746 | 0,49 | 0,48706117 | 1,00 |
| Ireland | 2007 | 25,2784004 | 0,13 | 1,13 | 1,12983716 | 0,49 | 0,48658435 | 1,00 |
| Ireland | 2008 | 26,6998646 | 0,17 | 1,18 | 1,17963992 | 0,49 | 0,48669145 | 1,00 |
| Ireland | 2009 | 26,6998646 | 0,19 | 1,18 | 1,1827029 | 0,49 | 0,4879321 | 1,00 |
| Ireland | 2010 | 26,6998646 | 0,19 | 1,24 | 1,23606698 | 0,49 | 0,49064193 | 0,50 |
| Ireland | 2011 |  | 0,18 | 1,28 | 1,27615063 | 0,49 | 0,4918027 | 0,50 |
| Ireland | 2012 |  | 0,17 | 1,21 | 1,21338912 | 0,49 | 0,49180792 | 0,50 |
| Ireland | 2013 |  | 0,16 | 1,27 | 1,26511628 | 0,49 | 0,49134472 | 0,50 |
| Ireland | 2014 |  | 0,14 |  | 1,26511628 | 0,49 | 0,49114112 | 0,50 |
| Ireland | 2015 |  | 0,10 |  | 1,26511628 | 0,49 | 0,493476 | 0,50 |
| Ireland | 2016 |  | 0,09 |  | 1,26511628 | 0,49 | 0,49324998 | 0,50 |
| Ireland | 2017 |  | 0,09 |  | 1,26511628 | 0,49 | 0,49360268 | 0,50 |
| Ireland | 2018 |  |  |  | 1,26511628 | 0,49 | 0,49114158 | 0,50 |
| Ireland | 2019 |  |  |  | 1,26511628 |  | 0,49114158 |  |
| Italy | 1980 | 32,3501958 | 0,12 |  | 0,89 | 0,35 | 0,3526728 | 0,00 |
| Italy | 1981 | 32,3501958 | 0,15 |  | 0,89 | 0,35 | 0,35220976 | 0,00 |
| Italy | 1982 | 32,3501958 | 0,15 |  | 0,89 | 0,35 | 0,35133779 | 0,00 |
| Italy | 1983 | 32,3501958 | 0,15 |  | 0,89 | 0,36 | 0,35753822 | 0,00 |
| Italy | 1984 | 32,3501958 | 0,13 |  | 0,89 | 0,36 | 0,35661109 | 0,00 |
| Italy | 1985 | 31,9592932 | 0,13 |  | 0,89 | 0,36 | 0,35542189 | 0,00 |
| Italy | 1986 | 31,9592932 | 0,13 |  | 0,89 | 0,35 | 0,35436373 | 0,00 |
| Italy | 1987 | 31,9592932 | 0,11 |  | 0,89 | 0,36 | 0,35568 | 0,00 |
| Italy | 1988 | 31,9592932 | 0,10 |  | 0,89 | 0,42 | 0,41998665 | 0,00 |
| Italy | 1989 | 31,9592932 | 0,09 |  | 0,89 | 0,41 | 0,40962889 | 0,00 |
| Italy | 1990 | 31,9592932 | 0,09 |  | 0,89 | 0,37 | 0,37438073 | 0,00 |
| Italy | 1991 | 31,9592932 | 0,11 |  | 0,89 | 0,37 | 0,37150726 | 0,00 |
| Italy | 1992 | 31,9592932 | 0,12 |  | 0,89 | 0,37 | 0,36933096 | 0,50 |
| Italy | 1993 | 31,9592932 | 0,11 |  | 0,89 | 0,37 | 0,37290816 | 0,50 |
| Italy | 1994 | 31,9592932 | 0,10 |  | 0,89 | 0,37 | 0,37232899 | 0,00 |
| Italy | 1995 | 31,9592932 | 0,10 |  | 0,89 | 0,37 | 0,37132583 | 0,50 |
| Italy | 1996 | 31,9592932 | 0,10 |  | 0,89 | 0,38 | 0,38057757 | 0,50 |
| Italy | 1997 | 31,9592932 | 0,11 |  | 0,89 | 0,38 | 0,38294663 | 0,50 |
| Italy | 1998 | 31,9592932 | 0,11 |  | 0,89 | 0,38 | 0,38269891 | 0,50 |
| Italy | 1999 | 34,9799044 | 0,13 |  | 0,89 | 0,39 | 0,38565069 | 0,50 |
| Italy | 2000 | 35,1872012 | 0,12 | 0,89 | 0,89 | 0,39 | 0,38740701 | 0,50 |
| Italy | 2001 | 35,1872012 | 0,13 |  | 0,893 | 0,39 | 0,38950488 | 0,00 |
| Italy | 2002 | 35,1872012 | 0,14 |  | 0,896 | 0,40 | 0,39599518 | 0,50 |
| Italy | 2003 | 35,1872012 | 0,17 |  | 0,899 | 0,39 | 0,39300833 | 0,50 |
| Italy | 2004 | 35,1872012 | 0,18 |  | 0,902 | 0,38 | 0,38413054 | 0,50 |
| Italy | 2005 | 35,1872012 | 0,17 |  | 0,905 | 0,39 | 0,38639058 | 0,50 |
| Italy | 2006 | 35,1872012 | 0,17 |  | 0,908 | 0,39 | 0,38928378 | 0,50 |
| Italy | 2007 | 35,1872012 | 0,17 |  | 0,911 | 0,39 | 0,39256855 | 0,50 |
| Italy | 2008 | 35,1872012 | 0,18 |  | 0,914 | 0,39 | 0,3927723 | 0,50 |
| Italy | 2009 | 35,4010135 | 0,19 |  | 0,917 | 0,40 | 0,39506749 | 0,50 |
| Italy | 2010 | 35,6148258 | 0,19 | 0,92 | 0,92 | 0,40 | 0,39844732 | 0,50 |
| Italy | 2011 |  | 0,19 |  | 0,89571429 | 0,40 | 0,39965185 | 0,50 |
| Italy | 2012 |  | 0,20 |  | 0,87142857 | 0,40 | 0,40180556 | 0,50 |
| Italy | 2013 |  | 0,20 |  | 0,84714286 | 0,40 | 0,40262163 | 0,50 |
| Italy | 2014 |  | 0,19 |  | 0,82285714 | 0,40 | 0,40370012 | 0,00 |
| Italy | 2015 |  | 0,18 |  | 0,79857143 | 0,40 | 0,4034735 | 0,50 |
| Italy | 2016 |  | 0,20 |  | 0,77428571 | 0,40 | 0,40488858 | 0,50 |
| Italy | 2017 |  | 0,22 | 0,75 | 0,75 | 0,41 | 0,40674577 | 0,50 |
| Italy | 2018 |  |  |  | 0,75 | 0,40 | 0,40054809 | 0,50 |
| Italy | 2019 |  |  |  | 0,75 |  | 0,40054809 |  |
| Japan | 1980 | 14,9609325 | 0,07 |  | 0,68 |  |  | 0,00 |
| Japan | 1981 | 14,9609325 | 0,08 |  | 0,68 |  |  | 0,00 |
| Japan | 1982 | 14,9609325 | 0,08 |  | 0,68 |  |  | 0,00 |
| Japan | 1983 | 14,9609325 | 0,08 | 0,68 | 0,68 |  |  | 0,00 |
| Japan | 1984 | 14,9609325 | 0,06 |  | 0,66333333 |  |  | 0,00 |
| Japan | 1985 | 14,9609325 | 0,07 |  | 0,64666667 |  |  | 0,00 |
| Japan | 1986 | 14,9609325 | 0,07 | 0,63 | 0,63 |  |  | 0,00 |
| Japan | 1987 | 14,9609325 | 0,06 |  | 0,62594961 |  |  | 0,00 |
| Japan | 1988 | 14,9609325 | 0,06 |  | 0,62189922 |  |  | 0,00 |
| Japan | 1989 | 15,3162986 | 0,05 |  | 0,61784884 |  |  | 0,00 |
| Japan | 1990 | 15,3162986 | 0,05 |  | 0,61379845 |  |  | 0,00 |
| Japan | 1991 | 15,3162986 | 0,05 |  | 0,60974806 |  |  | 0,00 |
| Japan | 1992 | 24,5558153 | 0,05 |  | 0,60569767 |  |  | 0,00 |
| Japan | 1993 | 24,5558153 | 0,06 |  | 0,60164729 |  |  | 0,00 |
| Japan | 1994 | 24,5558153 | 0,04 |  | 0,5975969 |  |  | 0,00 |
| Japan | 1995 | 29,9011221 | 0,02 |  | 0,59354651 |  |  | 0,00 |
| Japan | 1996 | 29,9011221 | 0,03 |  | 0,58949612 |  |  | 0,00 |
| Japan | 1997 | 29,9011221 | 0,03 |  | 0,58544574 |  |  | 0,00 |
| Japan | 1998 | 29,9011221 | 0,03 | 0,58 | 0,58139535 |  |  | 0,00 |
| Japan | 1999 | 29,9011221 | 0,03 | 0,56 | 0,56349206 |  |  | 0,00 |
| Japan | 2000 | 29,9011221 | 0,03 | 0,55 | 0,55060729 |  |  | 0,00 |
| Japan | 2001 | 33,1083062 | 0,04 | 0,55 | 0,55416667 |  |  | 0,00 |
| Japan | 2002 | 33,1083062 | 0,04 | 0,55 | 0,54661017 |  |  | 0,00 |
| Japan | 2003 | 33,1083062 | 0,04 | 0,54 | 0,54112554 |  |  | 0,00 |
| Japan | 2004 | 33,1083062 | 0,04 | 0,55 | 0,54910714 |  |  | 0,00 |
| Japan | 2005 | 33,1083062 | 0,05 | 0,55 | 0,55405405 |  |  | 0,00 |
| Japan | 2006 | 33,1083062 | 0,05 | 0,55 | 0,5470852 |  |  | 0,00 |
| Japan | 2007 | 33,1083062 | 0,05 | 0,56 | 0,5619469 |  |  | 0,00 |
| Japan | 2008 | 33,1083062 | 0,06 | 0,57 | 0,57142857 |  |  | 0,00 |
| Japan | 2009 | 33,1083062 | 0,07 | 0,58 | 0,57727273 |  |  | 0,00 |
| Japan | 2010 | 33,1083062 | 0,08 | 0,58 | 0,58256881 |  |  | 0,00 |
| Japan | 2011 |  | 0,09 | 0,59 | 0,5879389 |  |  | 0,00 |
| Japan | 2012 |  | 0,09 | 0,59 | 0,58837086 |  |  | 0,00 |
| Japan | 2013 |  | 0,09 | 0,59 | 0,59161491 |  |  | 0,00 |
| Japan | 2014 |  | 0,11 | 0,59 | 0,59202727 |  |  | 0,00 |
| Japan | 2015 |  | 0,12 | 0,60 | 0,60312081 |  |  | 0,00 |
| Japan | 2016 |  | 0,13 |  | 0,60312081 |  |  | 0,00 |
| Japan | 2017 |  | 0,13 |  | 0,60312081 |  |  | 0,00 |
| Japan | 2018 |  |  |  | 0,60312081 |  |  | 0,00 |
| Japan | 2019 |  |  |  | 0,60312081 |  |  |  |
| Latvia | 1980 |  |  |  | 1,42 |  |  |  |
| Latvia | 1981 |  |  |  | 1,42 |  |  |  |
| Latvia | 1982 |  |  |  | 1,42 |  |  |  |
| Latvia | 1983 |  |  |  | 1,42 |  |  |  |
| Latvia | 1984 |  |  |  | 1,42 |  |  |  |
| Latvia | 1985 |  |  |  | 1,42 |  |  |  |
| Latvia | 1986 |  |  |  | 1,42 |  |  |  |
| Latvia | 1987 |  |  |  | 1,42 |  |  |  |
| Latvia | 1988 |  |  |  | 1,42 |  |  |  |
| Latvia | 1989 |  |  |  | 1,42 |  |  |  |
| Latvia | 1990 |  |  |  | 1,42 |  |  |  |
| Latvia | 1991 |  |  |  | 1,42 |  |  | 0,50 |
| Latvia | 1992 |  |  |  | 1,42 |  |  | 0,50 |
| Latvia | 1993 |  |  |  | 1,42 |  |  | 0,50 |
| Latvia | 1994 |  |  |  | 1,42 |  |  | 0,50 |
| Latvia | 1995 |  |  |  | 1,42 |  |  | 0,50 |
| Latvia | 1996 |  |  |  | 1,42 |  |  | 0,50 |
| Latvia | 1997 |  | 0,21 |  | 1,42 |  |  | 0,50 |
| Latvia | 1998 |  | 0,28 |  | 1,42 |  |  | 0,50 |
| Latvia | 1999 |  | 0,32 |  | 1,42 |  |  | 0,50 |
| Latvia | 2000 |  | 0,30 |  | 1,42 |  |  | 0,50 |
| Latvia | 2001 |  | 0,31 | 1,42 | 1,42 |  |  | 0,00 |
| Latvia | 2002 |  | 0,26 |  | 1,595 |  |  | 0,00 |
| Latvia | 2003 |  | 0,23 |  | 1,77 |  |  | 0,00 |
| Latvia | 2004 |  | 0,21 |  | 1,945 |  |  | 0,50 |
| Latvia | 2005 |  | 0,34 | 2,12 | 2,12 |  |  | 0,50 |
| Latvia | 2006 |  | 0,34 |  | 2,06875 |  |  | 0,50 |
| Latvia | 2007 |  | 0,35 |  | 2,0175 |  |  | 0,50 |
| Latvia | 2008 |  | 0,52 |  | 1,96625 |  |  | 0,50 |
| Latvia | 2009 |  | 0,73 |  | 1,915 |  |  | 0,50 |
| Latvia | 2010 |  | 0,60 |  | 1,86375 |  |  | 0,50 |
| Latvia | 2011 |  | 0,38 |  | 1,8125 |  |  | 0,50 |
| Latvia | 2012 |  | 0,32 |  | 1,76125 |  |  | 0,50 |
| Latvia | 2013 |  | 0,45 | 1,71 | 1,71 |  |  | 0,50 |
| Latvia | 2014 |  | 0,51 |  | 1,71 |  |  | 0,50 |
| Latvia | 2015 |  | 0,59 |  | 1,71 |  |  | 0,50 |
| Latvia | 2016 |  | 0,67 |  | 1,71 |  |  | 0,50 |
| Latvia | 2017 |  | 0,65 |  | 1,71 |  |  | 0,50 |
| Latvia | 2018 |  |  |  | 1,71 |  |  | 0,50 |
| Latvia | 2019 |  |  |  | 1,71 |  |  |  |
| Lithuania | 1980 |  |  |  | 1,25 |  |  |  |
| Lithuania | 1981 |  |  |  | 1,25 |  |  |  |
| Lithuania | 1982 |  |  |  | 1,25 |  |  |  |
| Lithuania | 1983 |  |  |  | 1,25 |  |  |  |
| Lithuania | 1984 |  |  |  | 1,25 |  |  |  |
| Lithuania | 1985 |  |  |  | 1,25 |  |  |  |
| Lithuania | 1986 |  |  |  | 1,25 |  |  |  |
| Lithuania | 1987 |  |  |  | 1,25 |  |  |  |
| Lithuania | 1988 |  |  |  | 1,25 |  |  |  |
| Lithuania | 1989 |  |  |  | 1,25 |  |  |  |
| Lithuania | 1990 |  |  |  | 1,25 |  |  |  |
| Lithuania | 1991 |  |  |  | 1,25 |  |  |  |
| Lithuania | 1992 |  |  |  | 1,25 |  |  |  |
| Lithuania | 1993 |  |  |  | 1,25 |  |  |  |
| Lithuania | 1994 |  |  |  | 1,25 |  |  | 0,00 |
| Lithuania | 1995 |  |  |  | 1,25 |  |  | 1,00 |
| Lithuania | 1996 |  | 0,29 |  | 1,25 |  |  | 1,00 |
| Lithuania | 1997 |  | 0,34 |  | 1,25 |  |  | 0,50 |
| Lithuania | 1998 |  | 0,37 |  | 1,25 |  |  | 0,50 |
| Lithuania | 1999 |  | 0,43 |  | 1,25 |  |  | 1,00 |
| Lithuania | 2000 |  | 0,41 |  | 1,25 |  |  | 1,00 |
| Lithuania | 2001 |  | 0,31 | 1,25 | 1,25 |  |  | 0,50 |
| Lithuania | 2002 |  | 0,27 |  | 1,246 |  |  | 0,50 |
| Lithuania | 2003 |  | 0,26 |  | 1,242 |  |  | 0,50 |
| Lithuania | 2004 |  | 0,28 |  | 1,238 |  |  | 0,50 |
| Lithuania | 2005 |  | 0,29 |  | 1,234 |  |  | 1,00 |
| Lithuania | 2006 |  | 0,32 | 1,23 | 1,23 |  |  | 1,00 |
| Lithuania | 2007 |  | 0,44 |  | 1,20571429 |  |  | 1,00 |
| Lithuania | 2008 |  | 0,93 |  | 1,18142857 |  |  | 1,00 |
| Lithuania | 2009 |  | 1,64 |  | 1,15714286 |  |  | 0,50 |
| Lithuania | 2010 |  | 1,48 |  | 1,13285714 |  |  | 0,50 |
| Lithuania | 2011 |  | 1,05 |  | 1,10857143 |  |  | 1,00 |
| Lithuania | 2012 |  | 0,79 |  | 1,08428571 |  |  | 1,00 |
| Lithuania | 2013 |  | 0,60 | 1,06 | 1,06 |  |  | 1,00 |
| Lithuania | 2014 |  | 0,60 |  | 1,06 |  |  | 1,00 |
| Lithuania | 2015 |  | 0,64 |  | 1,06 |  |  | 1,00 |
| Lithuania | 2016 |  | 0,67 |  | 1,06 |  |  | 1,00 |
| Lithuania | 2017 |  | 0,68 |  | 1,06 |  |  | 1,00 |
| Lithuania | 2018 |  |  |  | 1,06 |  |  | 1,00 |
| Lithuania | 2019 |  |  |  | 1,06 |  |  |  |
| Luxembourg | 1980 | 24,2241555 | 0,17 |  | 0,77 |  |  | 1,00 |
| Luxembourg | 1981 | 24,2241555 | 0,20 |  | 0,77 |  |  | 1,00 |
| Luxembourg | 1982 | 24,2241555 | 0,16 |  | 0,77 |  |  | 1,00 |
| Luxembourg | 1983 | 24,2241555 | 0,20 |  | 0,77 |  |  | 1,00 |
| Luxembourg | 1984 | 24,2241555 | 0,19 |  | 0,77 |  |  | 1,00 |
| Luxembourg | 1985 | 24,2241555 | 0,19 |  | 0,77 |  |  | 1,00 |
| Luxembourg | 1986 | 24,2241555 | 0,19 |  | 0,77 |  |  | 1,00 |
| Luxembourg | 1987 | 24,2241555 | 0,18 |  | 0,77 |  |  | 1,00 |
| Luxembourg | 1988 | 48,0484957 | 0,18 |  | 0,77 |  |  | 1,00 |
| Luxembourg | 1989 | 48,0484957 | 0,18 |  | 0,77 |  |  | 1,00 |
| Luxembourg | 1990 | 48,0484957 | 0,38 |  | 0,77 |  |  | 1,00 |
| Luxembourg | 1991 | 48,0484957 | 0,39 |  | 0,77 |  |  | 1,00 |
| Luxembourg | 1992 | 48,0484957 | 0,40 |  | 0,77 |  |  | 1,00 |
| Luxembourg | 1993 | 48,0484957 | 0,47 |  | 0,77 |  |  | 1,00 |
| Luxembourg | 1994 | 48,0484957 | 0,53 |  | 0,77 |  |  | 1,00 |
| Luxembourg | 1995 | 48,0484957 | 0,53 |  | 0,77 |  |  | 1,00 |
| Luxembourg | 1996 | 48,0484957 | 0,53 |  | 0,77 |  |  | 1,00 |
| Luxembourg | 1997 | 48,0484957 | 0,53 |  | 0,77 |  |  | 1,00 |
| Luxembourg | 1998 | 48,0484957 | 0,52 |  | 0,77 |  |  | 1,00 |
| Luxembourg | 1999 | 80,5640588 | 0,53 |  | 0,77 |  |  | 1,00 |
| Luxembourg | 2000 | 78,7115448 | 0,65 |  | 0,77 |  |  | 1,00 |
| Luxembourg | 2001 | 79,0496765 | 0,41 |  | 0,77 |  |  | 1,00 |
| Luxembourg | 2002 | 78,7850038 | 0,43 | 0,77 | 0,77 |  |  | 1,00 |
| Luxembourg | 2003 | 78,3253409 | 0,45 |  | 0,77 |  |  | 1,00 |
| Luxembourg | 2004 | 78,0647047 | 0,46 |  | 0,77 |  |  | 1,00 |
| Luxembourg | 2005 | 77,5322683 | 0,46 | 0,77 | 0,77 |  |  | 1,00 |
| Luxembourg | 2006 | 78,4349826 | 0,43 |  | 0,762 |  |  | 1,00 |
| Luxembourg | 2007 | 78,0537988 | 0,40 |  | 0,754 |  |  | 1,00 |
| Luxembourg | 2008 | 77,5246753 | 0,42 |  | 0,746 |  |  | 0,50 |
| Luxembourg | 2009 | 77,0062741 | 0,47 |  | 0,738 |  |  | 0,50 |
| Luxembourg | 2010 | 76,498378 | 0,46 | 0,73 | 0,73 |  |  | 0,50 |
| Luxembourg | 2011 |  | 0,42 |  | 0,73 |  |  | 0,50 |
| Luxembourg | 2012 |  | 0,43 |  | 0,73 |  |  | 0,50 |
| Luxembourg | 2013 |  | 0,42 |  | 0,73 |  |  | 0,50 |
| Luxembourg | 2014 |  | 0,41 |  | 0,73 |  |  | 0,50 |
| Luxembourg | 2015 |  | 0,40 |  | 0,73 |  |  | 1,00 |
| Luxembourg | 2016 |  | 0,38 |  | 0,73 |  |  | 1,00 |
| Luxembourg | 2017 |  | 0,51 |  | 0,73 |  |  | 1,00 |
| Luxembourg | 2018 |  |  |  | 0,73 |  |  | 1,00 |
| Luxembourg | 2019 |  |  |  | 0,73 |  |  |  |
| Netherlands | 1980 | 23,5134234 | 0,04 | 0,36 | 0,36029412 | 0,66 | 0,66085088 |  |
| Netherlands | 1981 | 23,5134234 | 0,05 | 0,33 | 0,32839506 | 0,76 | 0,75734388 | 1,00 |
| Netherlands | 1982 | 23,5134234 | 0,04 | 0,33 | 0,32736573 | 0,77 | 0,7673683 | 1,00 |
| Netherlands | 1983 | 23,5134234 | 0,04 | 0,33 | 0,328125 | 0,77 | 0,77047515 | 1,00 |
| Netherlands | 1984 | 23,5134234 | 0,04 | 0,34 | 0,33597884 | 0,77 | 0,77244875 | 1,00 |
| Netherlands | 1985 | 23,5134234 | 0,04 | 0,31 | 0,30578512 | 0,77 | 0,77249997 | 1,00 |
| Netherlands | 1986 | 23,5134234 | 0,04 | 0,31 | 0,31476323 | 0,77 | 0,76997499 | 1,00 |
| Netherlands | 1987 | 23,5134234 | 0,05 | 0,35 | 0,34626039 | 0,77 | 0,76840941 | 1,00 |
| Netherlands | 1988 | 23,5134234 | 0,05 | 0,35 | 0,35422343 | 0,77 | 0,76897882 | 1,00 |
| Netherlands | 1989 | 23,5134234 | 0,05 | 0,45 | 0,4519774 | 0,77 | 0,76869983 | 1,00 |
| Netherlands | 1990 | 32,7529401 | 0,00 | 0,53 | 0,52941176 | 0,77 | 0,7716487 | 1,00 |
| Netherlands | 1991 | 32,7529401 | 0,00 | 0,56 | 0,55882353 | 0,77 | 0,77046063 | 1,00 |
| Netherlands | 1992 | 32,7529401 | 0,00 | 0,61 | 0,60606061 | 0,77 | 0,77005247 | 1,00 |
| Netherlands | 1993 | 33,4636722 | 0,00 | 0,59 | 0,5880597 | 0,76 | 0,75836558 | 1,00 |
| Netherlands | 1994 | 33,4636722 | 0,00 | 0,61 | 0,61042945 | 0,76 | 0,76194097 | 1,00 |
| Netherlands | 1995 | 33,4636722 | 0,02 | 0,61 | 0,60736196 | 0,77 | 0,77234161 | 1,00 |
| Netherlands | 1996 | 33,4636722 | 0,02 | 0,62 | 0,62460568 | 0,80 | 0,80067719 | 1,00 |
| Netherlands | 1997 | 33,4636722 | 0,02 | 0,63 | 0,62578616 | 0,81 | 0,80836192 | 1,00 |
| Netherlands | 1998 | 33,4636722 | 0,02 | 0,57 | 0,57439521 | 0,89 | 0,88814378 | 1,00 |
| Netherlands | 1999 | 33,4636722 | 0,02 | 0,58 | 0,58023885 | 0,89 | 0,88707249 | 1,00 |
| Netherlands | 2000 | 33,4636722 | 0,02 | 0,59 | 0,58527026 | 0,89 | 0,88779396 | 1,00 |
| Netherlands | 2001 | 33,4636722 | 0,02 | 0,57 | 0,56945879 | 0,89 | 0,88524196 | 1,00 |
| Netherlands | 2002 | 33,4636722 | 0,02 |  | 0,57271964 | 0,88 | 0,88319656 | 1,00 |
| Netherlands | 2003 | 33,4636722 | 0,02 | 0,58 | 0,57598049 | 0,87 | 0,87355772 | 1,00 |
| Netherlands | 2004 | 33,4636722 | 0,02 | 0,59 | 0,5853743 | 0,87 | 0,87360931 | 1,00 |
| Netherlands | 2005 | 33,4636722 | 0,02 | 0,60 | 0,60151538 | 0,87 | 0,87366779 | 1,00 |
| Netherlands | 2006 | 33,4636722 | 0,01 | 0,61 | 0,60748886 | 0,87 | 0,87314846 | 1,00 |
| Netherlands | 2007 | 33,4636722 | 0,01 | 0,61 | 0,60735915 | 0,87 | 0,87357126 | 1,00 |
| Netherlands | 2008 | 33,4636722 | 0,01 | 0,62 | 0,62366278 | 0,87 | 0,87063788 | 1,00 |
| Netherlands | 2009 | 38,7438423 | 0,01 | 0,64 | 0,64241599 | 0,87 | 0,87258008 | 1,00 |
| Netherlands | 2010 | 38,7241159 | 0,01 | 0,66 | 0,65940008 | 0,88 | 0,87557171 | 1,00 |
| Netherlands | 2011 |  | 0,01 | 0,67 | 0,67336853 | 0,87 | 0,87369286 | 1,00 |
| Netherlands | 2012 |  | 0,01 | 0,71 | 0,70966352 | 0,87 | 0,87416364 | 1,00 |
| Netherlands | 2013 |  | 0,01 | 0,74 | 0,73913028 | 0,87 | 0,86820006 | 1,00 |
| Netherlands | 2014 |  | 0,01 |  | 0,73913028 | 0,87 | 0,87118459 | 1,00 |
| Netherlands | 2015 |  | 0,12 |  | 0,73913028 | 0,87 | 0,87444275 | 1,00 |
| Netherlands | 2016 |  | 0,11 |  | 0,73913028 | 0,87 | 0,87065316 | 1,00 |
| Netherlands | 2017 |  | 0,12 |  | 0,73913028 | 0,87 | 0,86686931 | 1,00 |
| Netherlands | 2018 |  |  |  | 0,73913028 | 0,86 | 0,86175919 | 1,00 |
| Netherlands | 2019 |  |  |  | 0,73913028 |  | 0,86175919 | 1,00 |
| New Zealand | 1980 | 0 | 0,00 |  | 1,04379667 | 0,43 | 0,43292692 | 0,00 |
| New Zealand | 1981 | 0 | 0,00 |  | 1,04379667 | 0,43 | 0,43324805 | 0,00 |
| New Zealand | 1982 | 0 | 0,00 |  | 1,04379667 | 0,44 | 0,43601477 | 0,00 |
| New Zealand | 1983 | 0 | 0,00 |  | 1,04379667 | 0,44 | 0,43631085 | 0,00 |
| New Zealand | 1984 | 0 | 0,00 |  | 1,04379667 | 0,43 | 0,42977319 | 0,00 |
| New Zealand | 1985 | 0 | 0,00 |  | 1,04379667 | 0,43 | 0,43052077 | 0,00 |
| New Zealand | 1986 | 0 | 0,00 |  | 1,04379667 | 0,43 | 0,43174026 | 0,50 |
| New Zealand | 1987 | 0 | 0,00 |  | 1,04379667 | 0,44 | 0,44115306 | 0,50 |
| New Zealand | 1988 | 0 | 0,00 |  | 1,04379667 |  | 0,35586479 | 0,50 |
| New Zealand | 1989 | 0 | 0,00 |  | 1,04379667 |  | 0,27057653 | 0,50 |
| New Zealand | 1990 | 0 | 0,00 |  | 1,04379667 |  | 0,18528826 | 0,50 |
| New Zealand | 1991 | 0 | 0,00 |  | 1,04379667 | 0,10 | 0,1 | 0,00 |
| New Zealand | 1992 | 0 | 0,00 |  | 1,04379667 | 0,10 | 0,1 | 0,00 |
| New Zealand | 1993 | 0 | 0,00 |  | 1,04379667 | 0,10 | 0,1 | 0,00 |
| New Zealand | 1994 | 0 | 0,00 |  | 1,04379667 | 0,10 | 0,1 | 0,00 |
| New Zealand | 1995 | 0 | 0,00 |  | 1,04379667 | 0,10 | 0,1 | 0,00 |
| New Zealand | 1996 | 0 | 0,00 |  | 1,04379667 | 0,10 | 0,1 | 0,00 |
| New Zealand | 1997 | 0 | 0,00 | 1,04 | 1,04379667 | 0,10 | 0,1 | 0,00 |
| New Zealand | 1998 | 0 | 0,00 | 1,10 | 1,10280334 | 0,10 | 0,1 | 0,00 |
| New Zealand | 1999 | 0 | 0,00 | 1,10 | 1,1018295 | 0,10 | 0,1 | 0,00 |
| New Zealand | 2000 | 0 | 0,00 | 1,14 | 1,13846307 | 0,10 | 0,1 | 0,50 |
| New Zealand | 2001 | 0 | 0,00 | 1,13 | 1,12566106 | 0,10 | 0,10488088 | 0,50 |
| New Zealand | 2002 | 23,5134234 | 0,04 | 1,22 | 1,21606482 | 0,11 | 0,10954451 | 0,50 |
| New Zealand | 2003 | 23,5134234 | 0,04 | 1,26 | 1,26155296 | 0,11 | 0,11401754 | 0,50 |
| New Zealand | 2004 | 23,5134234 | 0,05 | 1,29 | 1,2929992 | 0,12 | 0,1183216 | 0,50 |
| New Zealand | 2005 | 23,8687895 | 0,06 | 1,26 | 1,26047367 | 0,13 | 0,13416408 | 0,50 |
| New Zealand | 2006 | 23,8687895 | 0,07 | 1,26 | 1,26119024 | 0,14 | 0,13638182 | 0,50 |
| New Zealand | 2007 | 23,8687895 | 0,07 | 1,35 | 1,3520306 | 0,14 | 0,13638182 | 0,50 |
| New Zealand | 2008 | 23,8687895 | 0,08 | 1,46 | 1,45507203 | 0,14 | 0,13711309 | 0,50 |
| New Zealand | 2009 | 23,8687895 | 0,08 | 1,34 | 1,33715789 | 0,14 | 0,14 | 0,50 |
| New Zealand | 2010 | 23,8687895 | 0,08 | 1,40 | 1,39687329 | 0,14 | 0,14142136 | 0,50 |
| New Zealand | 2011 |  | 0,07 | 1,40 | 1,39632957 | 0,14 | 0,14491377 | 0,50 |
| New Zealand | 2012 |  | 0,08 | 1,48 | 1,48275862 | 0,15 | 0,14696938 | 0,50 |
| New Zealand | 2013 |  | 0,07 | 1,45 | 1,45333333 | 0,15 | 0,14832397 | 0,50 |
| New Zealand | 2014 |  | 0,07 |  | 1,45333333 | 0,15 | 0,14899664 | 0,50 |
| New Zealand | 2015 |  | 0,09 |  | 1,45333333 | 0,15 | 0,1496663 | 0,50 |
| New Zealand | 2016 |  | 0,10 |  | 1,45333333 | 0,15 | 0,15165751 | 0,50 |
| New Zealand | 2017 |  | 0,10 |  | 1,45333333 | 0,15 | 0,15427249 | 0,50 |
| New Zealand | 2018 |  | 0,12 |  | 1,45333333 |  | 0,15427249 |  |
| New Zealand | 2019 |  |  |  | 1,45333333 |  | 0,15427249 |  |
| Norway | 1980 | 33,8190382 | 0,18 |  | 0,66 | 0,80 | 0,79982188 | 1,00 |
| Norway | 1981 | 33,8190382 |  |  | 0,66 | 0,80 | 0,79547134 | 1,00 |
| Norway | 1982 | 33,8190382 |  |  | 0,66 | 0,79 | 0,78792319 | 1,00 |
| Norway | 1983 | 34,4586971 |  | 0,66 | 0,66 | 0,79 | 0,78668275 | 1,00 |
| Norway | 1984 | 34,4586971 |  |  | 0,692 | 0,78 | 0,78462556 | 1,00 |
| Norway | 1985 | 33,8190382 | 0,19 |  | 0,724 | 0,78 | 0,77965075 | 1,00 |
| Norway | 1986 | 33,8190382 |  |  | 0,756 | 0,78 | 0,78159039 | 1,00 |
| Norway | 1987 | 33,8190382 |  |  | 0,788 | 0,78 | 0,7773507 | 1,00 |
| Norway | 1988 | 33,8190382 | 0,27 |  | 0,82 | 0,82 | 0,82119812 | 1,00 |
| Norway | 1989 | 34,8851363 | 0,33 |  | 0,852 | 0,83 | 0,82530976 | 1,00 |
| Norway | 1990 | 34,8851363 | 0,37 |  | 0,884 | 0,82 | 0,82417351 | 1,00 |
| Norway | 1991 | 35,5958684 | 0,44 |  | 0,916 | 0,82 | 0,82128645 | 1,00 |
| Norway | 1992 | 35,5958684 | 0,49 |  | 0,948 | 0,81 | 0,81319405 | 1,00 |
| Norway | 1993 | 38,0834306 | 0,54 | 0,98 | 0,98 | 0,81 | 0,81057844 | 1,00 |
| Norway | 1994 | 38,0834306 | 0,66 |  | 1,01 | 0,81 | 0,81013828 | 1,00 |
| Norway | 1995 | 38,0834306 | 0,66 |  | 1,04 | 0,81 | 0,81139 | 1,00 |
| Norway | 1996 | 38,0834306 | 0,64 |  | 1,07 | 0,80 | 0,80408909 | 1,00 |
| Norway | 1997 | 38,0834306 | 0,63 |  | 1,1 | 0,81 | 0,80500804 | 1,00 |
| Norway | 1998 | 41,5077752 | 0,71 |  | 1,13 | 0,80 | 0,79942081 | 1,00 |
| Norway | 1999 | 50,4840322 | 0,83 | 1,16 | 1,16 | 0,79 | 0,79186022 | 1,00 |
| Norway | 2000 | 49,7253119 | 0,77 |  | 1,16 | 0,79 | 0,78522332 | 1,00 |
| Norway | 2001 | 50,0627642 | 0,77 |  | 1,16 | 0,78 | 0,78152961 | 1,00 |
| Norway | 2002 | 49,8916057 | 0,73 | 1,16 | 1,16 | 0,78 | 0,77786855 | 1,00 |
| Norway | 2003 | 49,74063 | 0,75 |  | 1,19 | 0,82 | 0,82035859 | 1,00 |
| Norway | 2004 | 50,150072 | 0,72 |  | 1,22 | 0,82 | 0,82079901 | 1,00 |
| Norway | 2005 | 49,5403132 | 0,65 |  | 1,25 | 0,82 | 0,81628603 | 1,00 |
| Norway | 2006 | 51,6632692 | 0,57 | 1,28 | 1,28 | 0,82 | 0,82082231 | 1,00 |
| Norway | 2007 | 51,6377359 | 0,56 |  | 1,29333333 | 0,83 | 0,82810706 | 1,00 |
| Norway | 2008 | 51,5798914 | 0,54 |  | 1,30666667 | 0,83 | 0,8289806 | 1,00 |
| Norway | 2009 | 51,9287903 | 0,63 | 1,32 | 1,32 | 0,83 | 0,82817291 | 1,00 |
| Norway | 2010 | 51,9021502 | 0,63 |  | 1,31666667 | 0,83 | 0,82805602 | 1,00 |
| Norway | 2011 |  | 0,61 |  | 1,31333333 | 0,83 | 0,82786297 | 1,00 |
| Norway | 2012 |  | 0,60 | 1,31 | 1,31 | 0,83 | 0,82682195 | 1,00 |
| Norway | 2013 |  | 0,60 |  | 1,31 | 0,83 | 0,82638412 | 1,00 |
| Norway | 2014 |  | 0,63 |  | 1,31 | 0,83 | 0,82671193 | 1,00 |
| Norway | 2015 |  | 0,67 |  | 1,31 | 0,83 | 0,82582152 | 1,00 |
| Norway | 2016 |  | 0,68 |  | 1,31 | 0,82 | 0,82383644 | 1,00 |
| Norway | 2017 |  | 0,65 |  | 1,31 | 0,83 | 0,82833285 | 1,00 |
| Norway | 2018 |  |  |  | 1,31 | 0,83 | 0,82832091 | 1,00 |
| Norway | 2019 |  |  |  | 1,31 |  | 0,82832091 |  |
| Poland | 1980 |  |  |  | 1,23 |  | 0,26504494 |  |
| Poland | 1981 |  |  |  | 1,23 |  | 0,26504494 |  |
| Poland | 1982 |  |  |  | 1,23 |  | 0,26504494 |  |
| Poland | 1983 |  |  |  | 1,23 |  | 0,26504494 |  |
| Poland | 1984 |  |  |  | 1,23 |  | 0,26504494 |  |
| Poland | 1985 |  |  |  | 1,23 |  | 0,26504494 |  |
| Poland | 1986 |  |  |  | 1,23 |  | 0,26504494 |  |
| Poland | 1987 |  |  |  | 1,23 |  | 0,26504494 |  |
| Poland | 1988 |  |  |  | 1,23 |  | 0,26504494 |  |
| Poland | 1989 |  |  |  | 1,23 |  | 0,26504494 |  |
| Poland | 1990 |  |  |  | 1,23 | 0,27 | 0,26504494 | 0,00 |
| Poland | 1991 |  | 0,44 |  | 1,23 |  | 0,25997169 | 0,00 |
| Poland | 1992 |  | 0,42 |  | 1,23 |  | 0,25489844 | 0,00 |
| Poland | 1993 |  | 0,42 |  | 1,23 | 0,25 | 0,24982519 | 0,00 |
| Poland | 1994 |  | 0,44 |  | 1,23 |  | 0,244296 | 0,50 |
| Poland | 1995 |  | 0,32 |  | 1,23 |  | 0,23876681 | 0,50 |
| Poland | 1996 |  | 0,29 |  | 1,23 |  | 0,23323763 | 0,50 |
| Poland | 1997 |  | 0,27 |  | 1,23 |  | 0,22770844 | 0,50 |
| Poland | 1998 |  | 0,26 | 1,23 | 1,23 | 0,22 | 0,22217926 | 0,00 |
| Poland | 1999 |  | 0,25 |  | 1,29428571 |  | 0,21835845 | 0,00 |
| Poland | 2000 |  | 0,28 |  | 1,35857143 |  | 0,21453763 | 0,00 |
| Poland | 2001 |  | 0,32 |  | 1,42285714 |  | 0,21071682 | 0,50 |
| Poland | 2002 |  | 0,32 |  | 1,48714286 |  | 0,20689601 | 0,50 |
| Poland | 2003 |  | 0,28 |  | 1,55142857 | 0,20 | 0,2030752 | 0,50 |
| Poland | 2004 |  | 0,25 |  | 1,61571429 |  | 0,2020474 | 0,50 |
| Poland | 2005 |  | 0,23 | 1,68 | 1,68 |  | 0,2010196 | 0,50 |
| Poland | 2006 |  | 0,27 |  | 1,67285714 |  | 0,19999181 | 0,50 |
| Poland | 2007 |  | 0,26 |  | 1,66571429 | 0,20 | 0,19896401 | 0,50 |
| Poland | 2008 |  | 0,28 |  | 1,65857143 |  | 0,2038973 | 0,50 |
| Poland | 2009 |  | 0,32 |  | 1,65142857 |  | 0,20883059 | 0,50 |
| Poland | 2010 |  | 0,34 |  | 1,64428571 |  | 0,21376388 | 0,50 |
| Poland | 2011 |  | 0,32 |  | 1,63714286 |  | 0,21869718 | 0,00 |
| Poland | 2012 |  | 0,46 | 1,63 | 1,63 | 0,22 | 0,22363047 | 0,00 |
| Poland | 2013 |  | 0,49 |  | 1,63 |  | 0,2242912 | 0,00 |
| Poland | 2014 |  | 0,47 |  | 1,63 |  | 0,22495194 | 0,00 |
| Poland | 2015 |  | 0,65 |  | 1,63 |  | 0,22561268 | 0,00 |
| Poland | 2016 |  | 1,12 |  | 1,63 | 0,23 | 0,22627342 | 0,50 |
| Poland | 2017 |  | 1,28 |  | 1,63 |  | 0,22627342 | 0,50 |
| Poland | 2018 |  |  |  | 1,63 |  | 0,22627342 | 0,50 |
| Poland | 2019 |  |  |  | 1,63 |  | 0,22627342 |  |
| Portugal | 1980 | 23,6733382 | 0,09 |  | 0,82 |  | 0,48564857 | 0,00 |
| Portugal | 1981 | 23,6733382 | 0,10 |  | 0,82 |  | 0,48564857 | 0,00 |
| Portugal | 1982 | 23,6733382 | 0,10 |  | 0,82 |  | 0,48564857 | 0,00 |
| Portugal | 1983 | 23,6733382 | 0,09 |  | 0,82 |  | 0,48564857 | 0,00 |
| Portugal | 1984 | 28,2930965 | 0,09 |  | 0,82 | 0,49 | 0,48564857 | 0,00 |
| Portugal | 1985 | 28,2930965 | 0,08 |  | 0,82 |  | 0,48133631 | 0,00 |
| Portugal | 1986 | 28,2930965 | 0,07 |  | 0,82 | 0,48 | 0,47702405 | 0,00 |
| Portugal | 1987 | 28,2930965 | 0,07 |  | 0,82 |  | 0,47309353 | 0,50 |
| Portugal | 1988 | 28,2930965 | 0,07 |  | 0,82 |  | 0,469163 | 0,50 |
| Portugal | 1989 | 28,2930965 | 0,07 |  | 0,82 |  | 0,46523247 | 0,50 |
| Portugal | 1990 | 28,2930965 | 0,07 |  | 0,82 | 0,46 | 0,46130195 | 0,50 |
| Portugal | 1991 | 28,2930965 | 0,08 |  | 0,82 |  | 0,45386691 | 0,50 |
| Portugal | 1992 | 28,2930965 | 0,08 |  | 0,82 |  | 0,44643187 | 0,50 |
| Portugal | 1993 | 28,2930965 | 0,09 |  | 0,82 |  | 0,43899682 | 0,50 |
| Portugal | 1994 | 28,2930965 | 0,08 |  | 0,82 |  | 0,43156178 | 0,50 |
| Portugal | 1995 | 28,4885478 | 0,09 |  | 0,82 | 0,42 | 0,42412674 | 0,50 |
| Portugal | 1996 | 28,4885478 | 0,09 |  | 0,82 |  | 0,42358004 | 0,50 |
| Portugal | 1997 | 28,4885478 | 0,09 |  | 0,82 | 0,42 | 0,42303333 | 0,50 |
| Portugal | 1998 | 28,4885478 | 0,08 |  | 0,82 |  | 0,42448764 | 0,50 |
| Portugal | 1999 | 28,7931473 | 0,09 |  | 0,82 |  | 0,42594195 | 0,50 |
| Portugal | 2000 | 28,7931473 | 0,11 | 0,82 | 0,82 |  | 0,42739626 | 0,50 |
| Portugal | 2001 | 29,0469802 | 0,12 |  | 0,8125 |  | 0,42885057 | 0,50 |
| Portugal | 2002 | 29,0469802 | 0,12 |  | 0,805 | 0,43 | 0,43030489 | 0,00 |
| Portugal | 2003 | 29,0469802 | 0,15 |  | 0,7975 | 0,42 | 0,42381887 | 0,00 |
| Portugal | 2004 | 29,0469802 | 0,18 | 0,79 | 0,79 |  | 0,42597753 | 0,00 |
| Portugal | 2005 | 29,0469802 | 0,24 |  | 0,8 |  | 0,42813618 | 0,50 |
| Portugal | 2006 | 29,0469802 | 0,24 |  | 0,81 | 0,43 | 0,43029484 | 0,50 |
| Portugal | 2007 | 29,0469802 | 0,25 |  | 0,82 |  | 0,43258014 | 0,50 |
| Portugal | 2008 | 29,0469802 | 0,25 |  | 0,83 | 0,43 | 0,43486544 | 0,50 |
| Portugal | 2009 | 34,392287 | 0,30 |  | 0,84 |  | 0,45126429 | 0,50 |
| Portugal | 2010 | 34,392287 | 0,28 |  | 0,85 | 0,47 | 0,46766314 | 0,50 |
| Portugal | 2011 |  | 0,30 |  | 0,86 |  | 0,47194878 | 0,50 |
| Portugal | 2012 |  | 0,30 | 0,87 | 0,87 |  | 0,47623442 | 0,50 |
| Portugal | 2013 |  | 0,25 |  | 0,87 |  | 0,48052006 | 1,00 |
| Portugal | 2014 |  | 0,26 |  | 0,87 |  | 0,4848057 | 1,00 |
| Portugal | 2015 |  | 0,28 |  | 0,87 | 0,49 | 0,48909134 | 1,00 |
| Portugal | 2016 |  | 0,31 |  | 0,87 |  | 0,48909134 | 1,00 |
| Portugal | 2017 |  | 0,31 |  | 0,87 |  | 0,48909134 | 1,00 |
| Portugal | 2018 |  |  |  | 0,87 |  | 0,48909134 | 1,00 |
| Portugal | 2019 |  |  |  | 0,87 |  | 0,48909134 |  |
| Slovak Republic | 1980 |  |  |  | 0,8 |  |  |  |
| Slovak Republic | 1981 |  |  |  | 0,8 |  |  |  |
| Slovak Republic | 1982 |  |  |  | 0,8 |  |  |  |
| Slovak Republic | 1983 |  |  |  | 0,8 |  |  |  |
| Slovak Republic | 1984 |  |  |  | 0,8 |  |  |  |
| Slovak Republic | 1985 |  |  |  | 0,8 |  |  |  |
| Slovak Republic | 1986 |  |  |  | 0,8 |  |  |  |
| Slovak Republic | 1987 |  |  |  | 0,8 |  |  |  |
| Slovak Republic | 1988 |  |  |  | 0,8 |  |  |  |
| Slovak Republic | 1989 |  |  |  | 0,8 |  |  |  |
| Slovak Republic | 1990 |  |  |  | 0,8 |  |  |  |
| Slovak Republic | 1991 |  |  |  | 0,8 |  |  |  |
| Slovak Republic | 1992 |  |  |  | 0,8 |  |  |  |
| Slovak Republic | 1993 |  |  |  | 0,8 |  |  | 0,50 |
| Slovak Republic | 1994 |  |  |  | 0,8 |  |  | 0,50 |
| Slovak Republic | 1995 |  | 0,57 |  | 0,8 |  |  | 0,50 |
| Slovak Republic | 1996 |  | 0,56 |  | 0,8 |  |  | 0,00 |
| Slovak Republic | 1997 |  | 0,69 |  | 0,8 |  |  | 0,00 |
| Slovak Republic | 1998 |  | 0,70 |  | 0,8 |  |  | 0,00 |
| Slovak Republic | 1999 |  | 0,65 |  | 0,8 |  |  | 1,00 |
| Slovak Republic | 2000 |  | 0,58 | 0,80 | 0,8 |  |  | 1,00 |
| Slovak Republic | 2001 |  | 0,52 |  | 0,804 |  |  | 0,50 |
| Slovak Republic | 2002 |  | 0,47 |  | 0,808 |  |  | 0,50 |
| Slovak Republic | 2003 |  | 0,55 |  | 0,812 |  |  | 0,00 |
| Slovak Republic | 2004 |  | 0,49 |  | 0,816 |  |  | 0,00 |
| Slovak Republic | 2005 |  | 0,50 | 0,82 | 0,82 |  |  | 0,50 |
| Slovak Republic | 2006 |  | 0,49 |  | 0,83666667 |  |  | 0,50 |
| Slovak Republic | 2007 |  | 0,46 |  | 0,85333333 |  |  | 0,50 |
| Slovak Republic | 2008 |  | 0,44 |  | 0,87 |  |  | 0,50 |
| Slovak Republic | 2009 |  | 0,52 |  | 0,88666667 |  |  | 0,00 |
| Slovak Republic | 2010 |  | 0,60 |  | 0,90333333 |  |  | 0,00 |
| Slovak Republic | 2011 |  | 0,64 |  | 0,92 |  |  | 0,00 |
| Slovak Republic | 2012 |  | 0,64 |  | 0,93666667 |  |  | 0,00 |
| Slovak Republic | 2013 |  | 0,64 |  | 0,95333333 |  |  | 0,50 |
| Slovak Republic | 2014 |  | 0,62 | 0,97 | 0,97 |  |  | 0,50 |
| Slovak Republic | 2015 |  | 0,60 |  | 0,97 |  |  | 0,50 |
| Slovak Republic | 2016 |  | 0,62 |  | 0,97 |  |  | 0,50 |
| Slovak Republic | 2017 |  | 0,66 |  | 0,97 |  |  | 0,50 |
| Slovak Republic | 2018 |  |  |  | 0,97 |  |  |  |
| Slovak Republic | 2019 |  |  |  | 0,97 |  |  |  |
| Spain | 1980 | 46,2420328 | 0,05 |  | 0,79 | 0,61 | 0,61242735 | 0,00 |
| Spain | 1981 | 46,2420328 | 0,05 |  | 0,79 | 0,48 | 0,48401037 | 0,00 |
| Spain | 1982 | 46,2420328 | 0,05 |  | 0,79 | 0,48 | 0,48390357 | 0,00 |
| Spain | 1983 | 46,2420328 | 0,05 |  | 0,79 | 0,48 | 0,48286747 | 0,00 |
| Spain | 1984 | 46,2420328 | 0,05 |  | 0,79 | 0,48 | 0,48140726 | 0,00 |
| Spain | 1985 | 46,2420328 | 0,05 |  | 0,79 | 0,48 | 0,47898269 | 0,00 |
| Spain | 1986 | 46,2420328 | 0,05 |  | 0,79 | 0,48 | 0,47888101 | 0,00 |
| Spain | 1987 | 46,2420328 | 0,05 |  | 0,79 | 0,48 | 0,47954573 | 0,00 |
| Spain | 1988 | 46,2420328 | 0,05 |  | 0,79 | 0,48 | 0,48215406 | 0,00 |
| Spain | 1989 | 46,2420328 | 0,05 |  | 0,79 | 0,49 | 0,4891853 | 0,00 |
| Spain | 1990 | 46,5973988 | 0,07 |  | 0,79 | 0,50 | 0,49801028 | 0,00 |
| Spain | 1991 | 46,5973988 | 0,06 | 0,79 | 0,79 | 0,50 | 0,50275256 | 0,00 |
| Spain | 1992 | 46,5973988 | 0,07 |  | 0,80428571 | 0,50 | 0,50129739 | 0,50 |
| Spain | 1993 | 46,5973988 | 0,07 |  | 0,81857143 | 0,50 | 0,50024631 | 0,50 |
| Spain | 1994 | 51,9427056 | 0,07 |  | 0,83285714 | 0,51 | 0,50623152 | 0,50 |
| Spain | 1995 | 51,9427056 | 0,08 |  | 0,84714286 | 0,51 | 0,50843262 | 0,50 |
| Spain | 1996 | 51,9427056 | 0,11 |  | 0,86142857 | 0,51 | 0,51056994 | 0,50 |
| Spain | 1997 | 51,9427056 | 0,11 |  | 0,87571429 | 0,51 | 0,51353742 | 0,50 |
| Spain | 1998 | 51,9427056 | 0,11 | 0,89 | 0,89 | 0,52 | 0,51582 | 0,50 |
| Spain | 1999 | 51,9427056 | 0,12 |  | 0,83 | 0,52 | 0,51853529 | 0,50 |
| Spain | 2000 | 51,9427056 | 0,12 |  | 0,77 | 0,52 | 0,51925862 | 0,50 |
| Spain | 2001 | 51,9427056 | 0,13 | 0,71 | 0,71 | 0,52 | 0,52216541 | 0,50 |
| Spain | 2002 | 51,9427056 | 0,14 |  | 0,75333333 | 0,52 | 0,52270736 | 0,50 |
| Spain | 2003 | 51,9427056 | 0,14 |  | 0,79666667 | 0,52 | 0,52386056 | 0,50 |
| Spain | 2004 | 51,9427056 | 0,15 | 0,84 | 0,84 | 0,52 | 0,52452817 | 0,50 |
| Spain | 2005 | 51,9427056 | 0,15 |  | 0,83 | 0,52 | 0,51812947 | 0,50 |
| Spain | 2006 | 51,9427056 | 0,16 |  | 0,82 | 0,51 | 0,51358624 | 0,50 |
| Spain | 2007 | 51,9427056 | 0,23 |  | 0,81 | 0,53 | 0,52597096 | 0,50 |
| Spain | 2008 | 51,9427056 | 0,32 | 0,80 | 0,8 | 0,53 | 0,52911536 | 0,50 |
| Spain | 2009 | 51,9427056 | 0,34 |  | 0,8025 | 0,55 | 0,55369677 | 0,50 |
| Spain | 2010 | 51,9427056 | 0,02 |  | 0,805 | 0,56 | 0,55890963 | 0,50 |
| Spain | 2011 |  | 0,02 |  | 0,8075 | 0,56 | 0,56378849 | 0,50 |
| Spain | 2012 |  | 0,02 | 0,81 | 0,81 | 0,57 | 0,56628733 | 0,50 |
| Spain | 2013 |  | 0,02 |  | 0,81 | 0,57 | 0,56511443 | 0,50 |
| Spain | 2014 |  | 0,02 |  | 0,81 | 0,56 | 0,56065588 | 0,50 |
| Spain | 2015 |  | 0,02 |  | 0,81 | 0,56 | 0,55846623 | 0,50 |
| Spain | 2016 |  | 0,02 |  | 0,81 | 0,56 | 0,55891898 | 0,50 |
| Spain | 2017 |  | 0,04 |  | 0,81 | 0,56 | 0,55781477 | 0,50 |
| Spain | 2018 |  |  |  | 0,81 | 0,56 | 0,55521032 | 0,50 |
| Spain | 2019 |  |  |  | 0,81 |  | 0,55521032 |  |
| Sweden | 1980 | 38,321519 | 0,60 | 0,99 | 0,99496855 | 0,86 | 0,8594619 | 0,50 |
| Sweden | 1981 | 38,321519 | 0,64 | 1,00 | 1,00375469 | 0,86 | 0,86292773 | 0,50 |
| Sweden | 1982 | 38,1077067 | 0,64 | 1,01 | 1,00866337 | 0,86 | 0,86491757 | 1,00 |
| Sweden | 1983 | 38,1077067 | 0,60 | 1,03 | 1,03366584 | 0,73 | 0,72744759 | 1,00 |
| Sweden | 1984 | 38,1077067 | 0,58 | 1,04 | 1,03822441 | 0,73 | 0,7273716 | 1,00 |
| Sweden | 1985 | 35,969584 | 0,64 | 1,01 | 1,00730816 | 0,74 | 0,73714525 | 1,00 |
| Sweden | 1986 | 38,1017801 | 0,69 | 1,02 | 1,01608911 | 0,74 | 0,73862715 | 1,00 |
| Sweden | 1987 | 38,1017801 | 0,71 | 1,03 | 1,0250941 | 0,74 | 0,73814106 | 1,00 |
| Sweden | 1988 | 38,1017801 | 0,79 | 1,03 | 1,0343075 | 0,74 | 0,73740552 | 1,00 |
| Sweden | 1989 | 40,4116593 | 0,86 | 1,03 | 1,0343075 | 0,74 | 0,73620932 | 1,00 |
| Sweden | 1990 | 40,197847 | 1,02 | 1,05 | 1,04691358 | 0,73 | 0,73406245 | 1,00 |
| Sweden | 1991 | 44,4740925 | 1,08 | 1,06 | 1,05773956 | 0,74 | 0,74080931 | 0,50 |
| Sweden | 1992 | 44,4740925 | 1,14 | 1,06 | 1,05651106 | 0,74 | 0,73935383 | 0,50 |
| Sweden | 1993 | 44,4740925 | 0,96 | 1,05 | 1,04932182 | 0,74 | 0,73998556 | 0,50 |
| Sweden | 1994 | 44,4740925 | 0,98 | 1,06 | 1,05977584 | 0,74 | 0,74004211 | 0,50 |
| Sweden | 1995 | 39,5564102 | 0,78 | 1,06 | 1,06407035 | 0,73 | 0,73492848 | 1,00 |
| Sweden | 1996 | 39,5564102 | 0,59 | 1,06 | 1,05956907 | 0,73 | 0,73351416 | 1,00 |
| Sweden | 1997 | 38,4873488 | 0,52 | 1,06 | 1,06265985 | 0,72 | 0,71937136 | 1,00 |
| Sweden | 1998 | 42,3867363 | 0,52 | 1,06 | 1,06396867 | 0,72 | 0,71708316 | 1,00 |
| Sweden | 1999 | 42,3867363 | 0,52 | 1,07 | 1,06905636 | 0,72 | 0,71500036 | 1,00 |
| Sweden | 2000 | 38,9707579 | 0,51 | 1,07 | 1,06738392 | 0,71 | 0,71218925 | 1,00 |
| Sweden | 2001 | 38,9706072 | 0,52 | 1,06 | 1,05630202 | 0,71 | 0,7087235 | 1,00 |
| Sweden | 2002 | 39,9499434 | 0,56 | 1,07 | 1,06923198 | 0,71 | 0,70552159 | 1,00 |
| Sweden | 2003 | 39,9496677 | 0,60 | 1,08 | 1,07760764 | 0,70 | 0,70233989 | 1,00 |
| Sweden | 2004 | 39,9535446 | 0,63 | 1,07 | 1,06949042 | 0,70 | 0,7002236 | 1,00 |
| Sweden | 2005 | 39,9532216 | 0,62 | 1,07 | 1,07135563 | 0,70 | 0,69782066 | 1,00 |
| Sweden | 2006 | 39,9528097 | 0,62 | 1,08 | 1,07561066 | 0,70 | 0,69771728 | 1,00 |
| Sweden | 2007 | 39,9523982 | 0,63 | 1,07 | 1,07308645 | 0,70 | 0,69905085 | 1,00 |
| Sweden | 2008 | 42,8435404 | 0,66 | 1,08 | 1,08162765 | 0,70 | 0,70002316 | 1,00 |
| Sweden | 2009 | 42,7576431 | 0,70 | 1,08 | 1,07973468 | 0,70 | 0,69739149 | 1,00 |
| Sweden | 2010 | 42,6767019 | 0,70 | 1,07 | 1,07009301 | 0,71 | 0,70910338 | 1,00 |
| Sweden | 2011 |  | 0,69 | 1,08 | 1,07940994 | 0,71 | 0,70771782 | 1,00 |
| Sweden | 2012 |  | 0,71 | 1,08 | 1,07995103 | 0,71 | 0,71293364 | 1,00 |
| Sweden | 2013 |  | 0,73 | 1,08 | 1,08307444 | 0,71 | 0,71289284 | 1,00 |
| Sweden | 2014 |  | 0,71 |  | 0,89210381 | 0,71 | 0,71471313 | 1,00 |
| Sweden | 2015 |  | 0,68 |  | 0,70113318 | 0,72 | 0,7174168 | 1,00 |
| Sweden | 2016 |  | 0,69 |  | 0,51016255 | 0,72 | 0,71945052 | 1,00 |
| Sweden | 2017 |  | 0,68 | 0,32 | 0,31919192 | 0,72 | 0,71552041 | 1,00 |
| Sweden | 2018 |  |  |  | 0,31919192 |  | 0,71552041 |  |
| Sweden | 2019 |  |  |  | 0,31919192 |  | 0,71552041 |  |
| Switzerland | 1980 | 1,77683014 | 0,01 | 0,34 | 0,34080717 | 0,31 | 0,31368774 | 1,00 |
| Switzerland | 1981 | 1,77683014 | 0,01 | 0,34 | 0,34396355 | 0,31 | 0,31464265 | 1,00 |
| Switzerland | 1982 | 1,77683014 | 0,01 | 0,35 | 0,34722222 | 0,31 | 0,31368774 | 1,00 |
| Switzerland | 1983 | 1,77683014 | 0,01 | 0,34 | 0,34134615 | 0,31 | 0,31464265 | 1,00 |
| Switzerland | 1984 | 1,77683014 | 0,01 | 0,35 | 0,35207824 | 0,32 | 0,31559468 | 1,00 |
| Switzerland | 1985 | 1,77683014 | 0,01 | 0,35 | 0,35380835 | 0,32 | 0,31654384 | 1,00 |
| Switzerland | 1986 | 1,77683014 | 0,01 | 0,34 | 0,34146341 | 0,32 | 0,31749016 | 1,00 |
| Switzerland | 1987 | 1,77683014 | 0,01 | 0,34 | 0,33902439 | 0,32 | 0,31843367 | 1,00 |
| Switzerland | 1988 | 1,77683014 | 0,01 |  | 0,33911308 | 0,32 | 0,31937439 | 1,00 |
| Switzerland | 1989 | 1,77683014 | 0,01 |  | 0,33920177 | 0,32 | 0,32124757 | 1,00 |
| Switzerland | 1990 | 1,77683014 | 0,05 |  | 0,33929047 | 0,38 | 0,37629775 | 1,00 |
| Switzerland | 1991 | 1,77683014 | 0,05 |  | 0,33937916 | 0,38 | 0,37841776 | 1,00 |
| Switzerland | 1992 | 1,77683014 | 0,05 |  | 0,33946785 | 0,39 | 0,38884444 | 1,00 |
| Switzerland | 1993 | 1,77683014 | 0,07 |  | 0,33955654 | 0,38 | 0,38470768 | 1,00 |
| Switzerland | 1994 | 1,77683014 | 0,07 |  | 0,33964523 | 0,38 | 0,38157568 | 1,00 |
| Switzerland | 1995 | 1,77683014 | 0,06 |  | 0,33973392 | 0,38 | 0,38157568 | 1,00 |
| Switzerland | 1996 | 1,77683014 | 0,05 |  | 0,33982262 | 0,37 | 0,3720215 | 1,00 |
| Switzerland | 1997 | 2,84292822 | 0,04 |  | 0,33991131 | 0,37 | 0,36551334 | 1,00 |
| Switzerland | 1998 | 2,84292822 | 0,05 | 0,34 | 0,34 | 0,37 | 0,37094474 | 1,00 |
| Switzerland | 1999 | 2,84292822 | 0,04 |  | 0,36230769 | 0,37 | 0,36660606 | 1,00 |
| Switzerland | 2000 | 2,84292822 | 0,04 |  | 0,38461538 | 0,35 | 0,35194639 | 1,00 |
| Switzerland | 2001 | 2,84292822 | 0,05 |  | 0,40692308 | 0,35 | 0,35173258 | 1,00 |
| Switzerland | 2002 | 2,84292822 | 0,07 |  | 0,42923077 | 0,35 | 0,34743137 | 1,00 |
| Switzerland | 2003 | 2,84292822 | 0,05 |  | 0,45153846 | 0,34 | 0,33757899 | 1,00 |
| Switzerland | 2004 | 2,84292822 | 0,05 |  | 0,47384615 | 0,51 | 0,50650199 | 1,00 |
| Switzerland | 2005 | 2,84292822 | 0,07 |  | 0,49615385 | 0,50 | 0,50300683 | 1,00 |
| Switzerland | 2006 | 19,592544 | 0,12 |  | 0,51846154 | 0,50 | 0,50448608 | 1,00 |
| Switzerland | 2007 | 19,592544 | 0,12 |  | 0,54076923 | 0,51 | 0,50531301 | 1,00 |
| Switzerland | 2008 | 19,592544 | 0,13 |  | 0,56307692 | 0,51 | 0,50620582 | 1,00 |
| Switzerland | 2009 | 19,592544 | 0,14 |  | 0,58538462 | 0,49 | 0,4943616 | 1,00 |
| Switzerland | 2010 | 19,592544 | 0,15 |  | 0,60769231 | 0,50 | 0,49950019 | 1,00 |
| Switzerland | 2011 |  | 0,15 | 0,63 | 0,63 | 0,50 | 0,50283285 | 1,00 |
| Switzerland | 2012 |  | 0,15 |  | 0,63 | 0,51 | 0,50933608 | 1,00 |
| Switzerland | 2013 |  | 0,15 |  | 0,63 | 0,52 | 0,51585276 | 1,00 |
| Switzerland | 2014 |  | 0,16 |  | 0,63 | 0,52 | 0,52036572 | 1,00 |
| Switzerland | 2015 |  | 0,16 |  | 0,63 | 0,53 | 0,52542334 | 1,00 |
| Switzerland | 2016 |  | 0,17 |  | 0,63 | 0,53 | 0,53216539 | 1,00 |
| Switzerland | 2017 |  | 0,16 |  | 0,63 | 0,53 | 0,5329165 | 1,00 |
| Switzerland | 2018 |  | 0,17 |  | 0,63 |  | 0,5329165 |  |
| Switzerland | 2019 |  |  |  | 0,63 |  | 0,5329165 |  |
| United Kingdom | 1980 | 13,2474711 | 0,09 | 0,70 | 0,7 | 0,19 | 0,18814888 | 0,00 |
| United Kingdom | 1981 | 13,0336588 | 0,08 |  | 0,70517058 | 0,19 | 0,18973666 | 0,00 |
| United Kingdom | 1982 | 11,7507852 | 0,08 |  | 0,71034115 | 0,19 | 0,18973666 | 0,00 |
| United Kingdom | 1983 | 10,8955361 | 0,07 |  | 0,71551173 | 0,19 | 0,18973666 | 0,00 |
| United Kingdom | 1984 | 10,6817238 | 0,07 | 0,72 | 0,7206823 | 0,19 | 0,18973666 | 0,00 |
| United Kingdom | 1985 | 10,4679115 | 0,07 | 0,74 | 0,74008811 | 0,19 | 0,19131126 | 0,00 |
| United Kingdom | 1986 | 9,61266243 | 0,06 | 0,80 | 0,80382775 | 0,19 | 0,18973666 | 0,00 |
| United Kingdom | 1987 | 9,39885015 | 0,06 | 0,79 | 0,7875 | 0,19 | 0,18973666 | 0,00 |
| United Kingdom | 1988 | 8,97122561 | 0,06 | 0,82 | 0,82058047 | 0,20 | 0,20493902 | 0,00 |
| United Kingdom | 1989 | 13,0336588 | 0,06 | 0,87 | 0,8700565 | 0,20 | 0,20493902 | 0,00 |
| United Kingdom | 1990 | 12,8198465 | 0,09 | 0,85 | 0,84857143 | 0,17 | 0,16613248 | 0,00 |
| United Kingdom | 1991 | 12,8198465 | 0,09 | 0,88 | 0,88409166 | 0,20 | 0,20493902 | 0,00 |
| United Kingdom | 1992 | 13,6750956 | 0,10 | 0,85 | 0,84857143 | 0,23 | 0,232379 | 0,00 |
| United Kingdom | 1993 | 12,6060342 | 0,09 | 0,89 | 0,88588589 | 0,23 | 0,23366643 | 0,00 |
| United Kingdom | 1994 | 12,8198465 | 0,07 | 0,91 | 0,91277259 | 0,19 | 0,19183326 | 0,00 |
| United Kingdom | 1995 | 12,8198465 | 0,06 | 0,92 | 0,91666667 | 0,19 | 0,19287302 | 0,00 |
| United Kingdom | 1996 | 12,8198465 | 0,06 | 0,92 | 0,91909385 | 0,19 | 0,19183326 | 0,00 |
| United Kingdom | 1997 | 12,6060342 | 0,06 | 0,96 | 0,95723684 | 0,19 | 0,19183326 | 0,00 |
| United Kingdom | 1998 | 9,84928396 | 0,06 | 0,96 | 0,95652174 | 0,19 | 0,19078784 | 0,00 |
| United Kingdom | 1999 | 14,4644529 | 0,07 | 1,01 | 1,01045296 | 0,20 | 0,2 | 0,00 |
| United Kingdom | 2000 | 14,4617402 | 0,48 | 1,02 | 1,01718213 | 0,20 | 0,2 | 0,00 |
| United Kingdom | 2001 | 14,4506387 | 0,48 | 1,02 | 1,0245614 | 0,20 | 0,20493902 | 0,00 |
| United Kingdom | 2002 | 14,4449191 | 0,47 | 1,07 | 1,06859206 | 0,20 | 0,20493902 | 0,00 |
| United Kingdom | 2003 | 14,4727008 | 0,11 | 1,09 | 1,09225092 | 0,21 | 0,20688161 | 0,00 |
| United Kingdom | 2004 | 13,8936186 | 0,11 | 1,12 | 1,11698113 | 0,21 | 0,2078461 | 0,00 |
| United Kingdom | 2005 | 13,8891676 | 0,10 | 1,14 | 1,14007782 | 0,21 | 0,20976177 | 0,00 |
| United Kingdom | 2006 | 13,8904753 | 0,33 | 1,17 | 1,16996047 | 0,21 | 0,20976177 | 0,00 |
| United Kingdom | 2007 | 14,6510532 | 0,14 | 1,23 | 1,23012552 | 0,23 | 0,22803509 | 0,00 |
| United Kingdom | 2008 | 14,6533695 | 0,16 | 1,22 | 1,22222222 | 0,23 | 0,22803509 | 0,00 |
| United Kingdom | 2009 | 14,6533695 | 0,17 | 1,22 | 1,2212766 | 0,23 | 0,22803509 | 0,00 |
| United Kingdom | 2010 | 14,6533695 | 0,17 | 1,23 | 1,23043478 | 0,23 | 0,22978251 | 0,00 |
| United Kingdom | 2011 |  | 0,16 | 1,24 | 1,24215247 | 0,23 | 0,22803509 | 0,00 |
| United Kingdom | 2012 |  | 0,16 | 1,27 | 1,2706422 | 0,23 | 0,22538855 | 0,00 |
| United Kingdom | 2013 |  | 0,16 | 1,23 | 1,22641509 | 0,23 | 0,22538855 | 0,00 |
| United Kingdom | 2014 |  | 0,15 | 1,22 | 1,21904762 | 0,23 | 0,22538855 | 0,00 |
| United Kingdom | 2015 |  | 0,15 | 1,27 | 1,26570048 | 0,23 | 0,22538855 | 0,00 |
| United Kingdom | 2016 |  | 0,15 |  | 1,26570048 | 0,23 | 0,22538855 | 0,00 |
| United Kingdom | 2017 |  | 0,14 |  | 1,26570048 | 0,23 | 0,22891046 | 0,00 |
| United Kingdom | 2018 |  |  |  | 1,26570048 | 0,23 | 0,22891046 | 0,00 |
| United Kingdom | 2019 |  |  |  | 1,26570048 |  | 0,22891046 |  |
| United States | 1980 | 0 |  |  | 0,59109312 | 0,14 | 0,1356466 | 0,00 |
| United States | 1981 | 0 |  | 0,59 | 0,59109312 | 0,14 | 0,1356466 | 0,00 |
| United States | 1982 | 0 |  |  | 0,59418909 | 0,14 | 0,1356466 | 0,00 |
| United States | 1983 | 0 |  | 0,60 | 0,59728507 | 0,14 | 0,1356466 | 0,00 |
| United States | 1984 | 0 |  | 0,60 | 0,6 | 0,14 | 0,1356466 | 0,00 |
| United States | 1985 | 0 |  | 0,64 | 0,63959391 | 0,14 | 0,14422205 | 0,00 |
| United States | 1986 | 0 |  | 0,62 | 0,61764706 | 0,14 | 0,14422205 | 0,00 |
| United States | 1987 | 0 |  | 0,64 | 0,63959391 | 0,14 | 0,14422205 | 0,00 |
| United States | 1988 | 0 |  |  | 0,65218097 | 0,14 | 0,14422205 | 0,00 |
| United States | 1989 | 0 |  |  | 0,66476803 | 0,14 | 0,14422205 | 0,00 |
| United States | 1990 | 0 |  |  | 0,67735509 | 0,14 | 0,14422205 | 0,00 |
| United States | 1991 | 0 |  |  | 0,68994216 | 0,14 | 0,14422205 | 0,00 |
| United States | 1992 | 0 |  |  | 0,70252922 | 0,14 | 0,14422205 | 0,00 |
| United States | 1993 | 2,13219616 |  | 0,72 | 0,71511628 | 0,16 | 0,16492423 | 0,00 |
| United States | 1994 | 2,13219616 |  |  | 0,72723362 | 0,16 | 0,16492423 | 0,00 |
| United States | 1995 | 2,13219616 |  |  | 0,73935097 | 0,16 | 0,16492423 | 0,00 |
| United States | 1996 | 2,13219616 |  |  | 0,75146831 | 0,16 | 0,16492423 | 0,00 |
| United States | 1997 | 2,13219616 |  |  | 0,76358565 | 0,16 | 0,16492423 | 0,00 |
| United States | 1998 | 2,13219616 |  | 0,78 | 0,775703 | 0,18 | 0,17663522 | 0,00 |
| United States | 1999 | 2,13219616 |  | 0,79 | 0,79446672 | 0,18 | 0,17663522 | 0,00 |
| United States | 2000 | 2,13219616 |  | 0,82 | 0,81671837 | 0,18 | 0,17663522 | 0,00 |
| United States | 2001 | 2,13219616 |  | 0,82 | 0,82327525 | 0,22 | 0,21725561 | 0,00 |
| United States | 2002 | 2,13219616 |  | 0,83 | 0,82983142 | 0,22 | 0,21908902 | 0,00 |
| United States | 2003 | 2,13219616 |  | 0,86 | 0,86080581 | 0,22 | 0,22271057 | 0,00 |
| United States | 2004 | 2,13219616 |  | 0,87 | 0,87090569 | 0,23 | 0,22627417 | 0,00 |
| United States | 2005 | 2,13219616 |  | 0,88 | 0,88405445 | 0,19 | 0,18973666 | 0,00 |
| United States | 2006 | 2,13219616 |  | 0,89 | 0,8882783 | 0,19 | 0,19390719 | 0,00 |
| United States | 2007 | 2,13219616 |  | 0,88 | 0,88162818 | 0,20 | 0,2 | 0,00 |
| United States | 2008 | 2,13219616 |  | 0,91 | 0,91155167 | 0,21 | 0,20976177 | 0,00 |
| United States | 2009 | 2,13219616 |  | 0,93 | 0,93128338 | 0,21 | 0,20976177 | 0,00 |
| United States | 2010 | 2,13219616 |  | 0,90 | 0,90416482 | 0,22 | 0,21908902 | 0,00 |
| United States | 2011 |  |  | 0,91 | 0,91474789 | 0,22 | 0,21908902 | 0,00 |
| United States | 2012 |  |  | 0,93 | 0,92671409 | 0,22 | 0,21908902 | 0,00 |
| United States | 2013 |  |  | 0,95 | 0,95160323 | 0,22 | 0,21908902 | 0,00 |
| United States | 2014 |  |  | 0,91 | 0,91071429 | 0,22 | 0,21908902 | 0,00 |
| United States | 2015 |  |  | 0,92 | 0,92173913 | 0,23 | 0,22803509 | 0,00 |
| United States | 2016 |  |  | 0,92 | 0,91891892 | 0,22 | 0,22449944 | 0,00 |
| United States | 2017 |  |  | 0,88 | 0,87719298 | 0,22 | 0,22449944 | 0,00 |
| United States | 2018 |  |  | 0,89 | 0,89189189 | 0,22 | 0,22449944 | 0,00 |
| United States | 2019 |  |  |  | 0,89189189 |  | 0,22449944 |  |

**Bibliography**

Armingeon, K., Engler, S., & Leemann, L. (2021). *Comparative Political Data Set 1960-2019*. Zurich: Institute of Political Science, University of Zurich.

Brady, D., Huber, E., & Stephens, J. D. (2020). *Comparative Welfare States Data Set*. University of North Carolina and WZB Berlin Social Science Center.

Gauthier, A. H. (2011). *Comparative Family Policy Database, Version 3 [computer file]*. Netherlands Interdisciplinary Demographic Institute and Max Planck Institute for Demographic Research (Distributors).

OECD. (2021). *OECD Statistics*.

OECD and AIAS (2021), Institutional Characteristics of Trade Unions, Wage Setting, State Intervention and Social Pacts, OECD Publishing, Paris.

Skorge, O. S., & Rasmussen, M. B. (2021). Volte-face on the Welfare State: Social Partners,

Knowledge Economies, and the Expansion of Work-Family Policies. *Politics & Society*, 1–33.

Visser, J. (2019). *ICTWSS Data Base on Insititutional Charachteristics of Trade Unions Wage Setting State Intervention and Social Pacts. Version 6.1.*
